# Discovery and heterologous biosynthesis of glycosylated polyketide luteodienoside A reveals unprecedented glucinol-mediated product offloading by a fungal carnitine *O*-acyltransferase domain†

**DOI:** 10.1039/d3sc05008d

**Published:** 2024-01-29

**Authors:** Amr A. Arishi, Zhuo Shang, Ernest Lacey, Andrew Crombie, Daniel Vuong, Hang Li, Joe Bracegirdle, Peter Turner, William Lewis, Gavin R. Flematti, Andrew M. Piggott, Yit-Heng Chooi

**Affiliations:** a School of Molecular Sciences, The University of Western Australia Perth WA 6009 Australia yitheng.chooi@uwa.edu.au; b Department of Botany and Microbiology, College of Science, King Saud University Riyadh 11451 Saudi Arabia; c School of Pharmaceutical Sciences, Shandong University Jinan Shandong 250012 China; d School of Natural Sciences, Macquarie University Sydney NSW 2109 Australia andrew.piggott@mq.edu.au; e Microbial Screening Technologies Pty. Ltd Smithfield NSW 2164 Australia; f School of Pharmaceutical Sciences, Sun Yat-sen University Guangzhou 510006 China; g School of Chemistry, The University of Sydney NSW 2006 Australia

## Abstract

Luteodienoside A is a novel glycosylated polyketide produced by the Australian fungus *Aspergillus luteorubrus* MST-FP2246, consisting of an unusual 1-*O*-β-d-glucopyranosyl-*myo*-inositol (glucinol) ester of 3-hydroxy-2,2,4-trimethylocta-4,6-dienoic acid. Mining the genome of *A. luteorubrus* identified a putative gene cluster for luteodienoside A biosynthesis (*ltb*), harbouring a highly reducing polyketide synthase (HR-PKS, LtbA) fused at its C-terminus to a carnitine *O*-acyltransferase (cAT) domain. Heterologous pathway reconstitution in *Aspergillus nidulans*, substrate feeding assays and gene truncation confirmed the identity of the *ltb* cluster and demonstrated that the cAT domain is essential for offloading luteodienoside A from the upstream HR-PKS. Unlike previously characterised cAT domains, the LtbA cAT domain uses glucinol as an offloading substrate to release the product from the HR-PKS. Furthermore, the PKS methyltransferase (MT) domain is capable of catalysing *gem*-dimethylation of the 3-hydroxy-2,2,4-trimethylocta-4,6-dienoic acid intermediate, without requiring reversible product release and recapture by the cAT domain. This study expands the repertoire of polyketide modifications known to be catalysed by cAT domains and highlights the potential of mining fungal genomes for this subclass of fungal PKSs to discover new structurally diverse secondary metabolites.

## Abstract 2

Luteodienoside A – a glycosylated polyketide produced by the Australian fungus *Aspergillus luteorubrus* is biosynthesised by a polyketide synthase with a carnitine *O*-acyltransferase domain capable of adding glucinol to the polyketide product.

## Graphical abstract

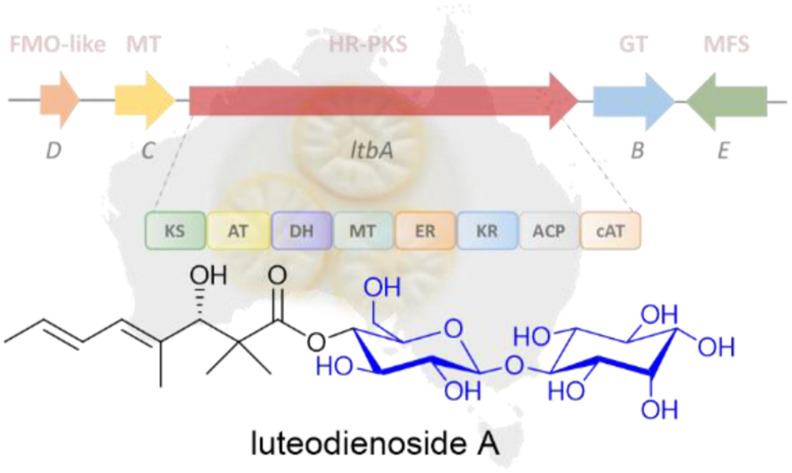

## Introduction

Fungal glycosides are a large and diverse class of secondary metabolites with significant biological activities and promising pharmaceutical applications.^1^ Among the fungal glycosides, glycosylated polyketides are particularly noteworthy for their broad spectrum of bioactivities. Some examples include the antifungal burnettramic acids from *Aspergillus burnettii*,^2^ the cytotoxic cladionol A from *Gliocladium* sp.,^3^ and the antibacterial epicoccamide from *Epicoccum purpurascens*.^4,5^ However, despite these diverse bioactivities, the biosynthesis of glycosylated polyketides is still not well understood in fungi.

Technological advances in next-generation DNA sequencing and bioinformatics tools, coupled with an expanding knowledge of fungal secondary metabolite biosynthetic pathways, have seen genome mining emerge as an effective approach for the discovery of novel bioactive molecules.^6^ It is well known that biosynthetic genes are clustered together in fungal genomes. Reconstitution of these biosynthetic gene clusters (BGCs) in heterologous expression hosts allows the function of each individual gene in a biosynthetic pathway to be interrogated, making it a powerful approach to link genes to biosynthetic steps and chemical structures.^7^ These genes can then serve as beacons to guide genome mining for structurally related molecules. Hence, we became interested in dissecting the genetic and biochemical basis for glycosylated polyketides in fungi, with a view to facilitating targeted genome mining for new members of this important group of metabolites.

As part of our ongoing chemotaxonomic exploration of endemic Australian Aspergilli,^2,8–10^ we isolated a novel species, *Aspergillus luteorubrus* MST-FP2246, from a soil sample collected at White Mountains National Park in northern Queensland, Australia.^11^ Herein, we report the discovery and structural characterisation of luteodienoside A (1), an abundant polar metabolite produced by *A. luteorubrus* featuring a novel glucinol ester of a *gem*-dimethylated polyketide. We employed heterologous pathway reconstitution, polyketide synthase (PKS) domain truncation, substrate feeding experiments and characterisation of pathway intermediates to elucidate the biosynthetic pathway to 1.

## Results and discussion

### Isolation and characterisation of luteodienoside A from *Aspergillus luteorubrus*

*A*. *luteorubrus* MST-FP2246 was cultivated on basmati rice (2.0 kg) for 21 days at 24 °C. The grains were then extracted with acetone and the extract partitioned between ethyl acetate and water. The aqueous partition was fractionated by both reversed phase and normal phase silica gel chromatography to give an enriched extract (1.9 g, ∼60% pure). A sub-sample of the extract (40 mg) was subjected to size exclusion chromatography on Sephadex LH-20, then reversed phase HPLC on C_18_ silica, yielding pure luteodienoside A (1) as a light-yellow solid (22 mg).

**Fig. 1:**
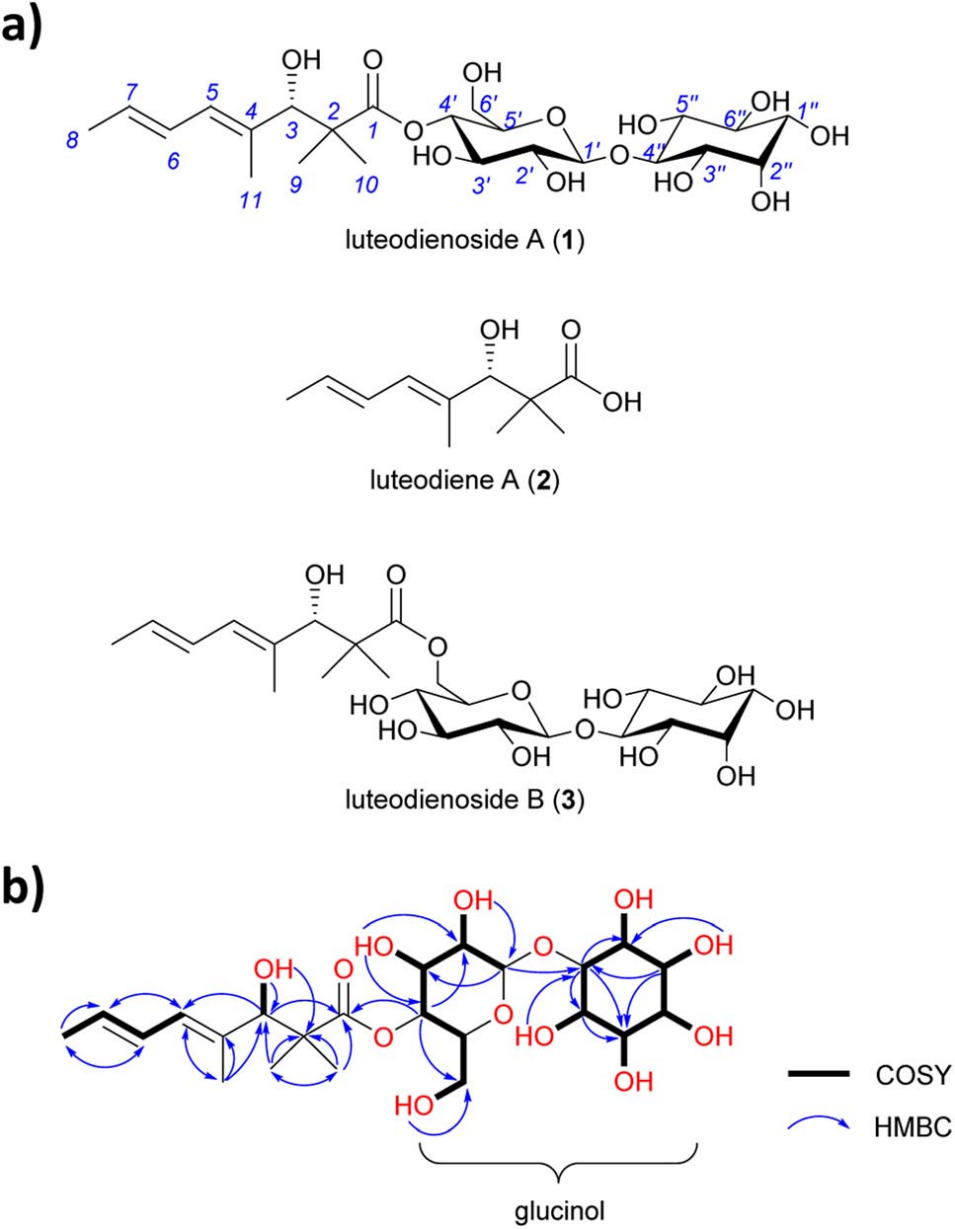
(a) Structures of luteodienoside A (1), luteodiene A (2) and luteodienoside B (3) reported in this study. (b) Diagnostic 2D NMR correlations for 1.

HRESI(+)MS analysis of 1 revealed a dehydrated protonated molecule at *m*/*z* 505.2274 [M − H_2_O + H]^+^, indicative of a molecular formula C_23_H_38_O_13_ requiring five double bond equivalents (DBEs). The UV-vis spectrum of 1 (Fig. S6†) showed a single *λ*_max_ at 240 nm, characteristic of an isolated diene. Analysis of the NMR data for 1 in DMSO-*d*_6_ (Table S5†) revealed resonances for four methyl groups (*δ*_C-8_ 18.2, *δ*_C-9_ 23.0, *δ*_C-10_ 19.4, *δ*_C-11_ 14.3), four olefinic carbons (*δ*_C-4_ 134.6, *δ*_C-5_ 127.5, *δ*_C-6_ 127.4, *δ*_C-7_ 128.8) and one carbonyl group (*δ*_C-1_ 175.7), along with twelve oxygenated methines. COSY correlations spanning from H-5 to H-8 and HMBC correlations from H-11 to C-3 and C-5, and from H_3_-9/10 to C-1, C-2 and C-3, permitted assignment of a 3-hydroxy-2,2,4-trimethylpent-4-enoyl moiety (Fig. 1b). A large ^3^*J*_H-6,7_ coupling constant (14.5 Hz) and a ROESY correlation between H-6 and H-11 indicated a 4*E*,6*E* geometry for the diene. Based on the chemical shifts of the remaining 12 carbons (*δ*_C_ 60–100) and two remaining DBEs, the presence of two hexose moieties in the structure of 1 was hypothesised. COSY correlations spanning H-1′ (*δ*_H_ 4.41) to H_2_-6′ (*δ*_H_ 3.28/3.47) defined the first hexose moiety, with an HMBC correlation from H-4′ (*δ*_H_ 4.52) to C-1 (*δ*_C_ 175.7) confirming an ester linkage between C-1 and C-4′. COSY correlations spanning H-1′′ to H-6′′ defined the second hexose moiety, with the absence of an anomeric centre and methylene group indicative of a carbocyclic sugar (inositol). An HMBC correlation from H-1′ (*δ*_H_ 4.41) to C-4′′ (*δ*_C_ 85.3) confirmed a 1,4-glycosidic linkage between the two sugars, thus completing the planar structure of 1. Finally, single crystal X-ray diffraction analysis (Fig. S1†) confirmed the absolute stereostructure of 1 as being the 1-*O*-β-d-glucopyranosyl-*myo*-inositol (glucinol) ester of 3*S*-hydroxy-2,2,4-trimethylocta-4,6-dienoic acid (Fig. 1a).

### Identification of luteodienoside A BGC featuring an HR-PKS harbouring a carnitine *O*-acyltransferase (cAT) domain

The unprecedented conjugation between glucinol and a polyketide chain in 1 prompted us to investigate the molecular basis for its biosynthesis. Retrobiosynthetic analysis of 1 suggested that the trimethylated polyketide chain is derived from acetyl-CoA, malonyl-CoA and *S*-adenosylmethionine (SAM), assembled by a highly reducing polyketide synthase (HR-PKS) containing a *C*-methyltransferase domain (MT). Additionally, the presence of glucinol suggested a requirement for one or more glycosyltransferase-encoding genes within the luteodienoside A BGC.

**Fig. 2:**
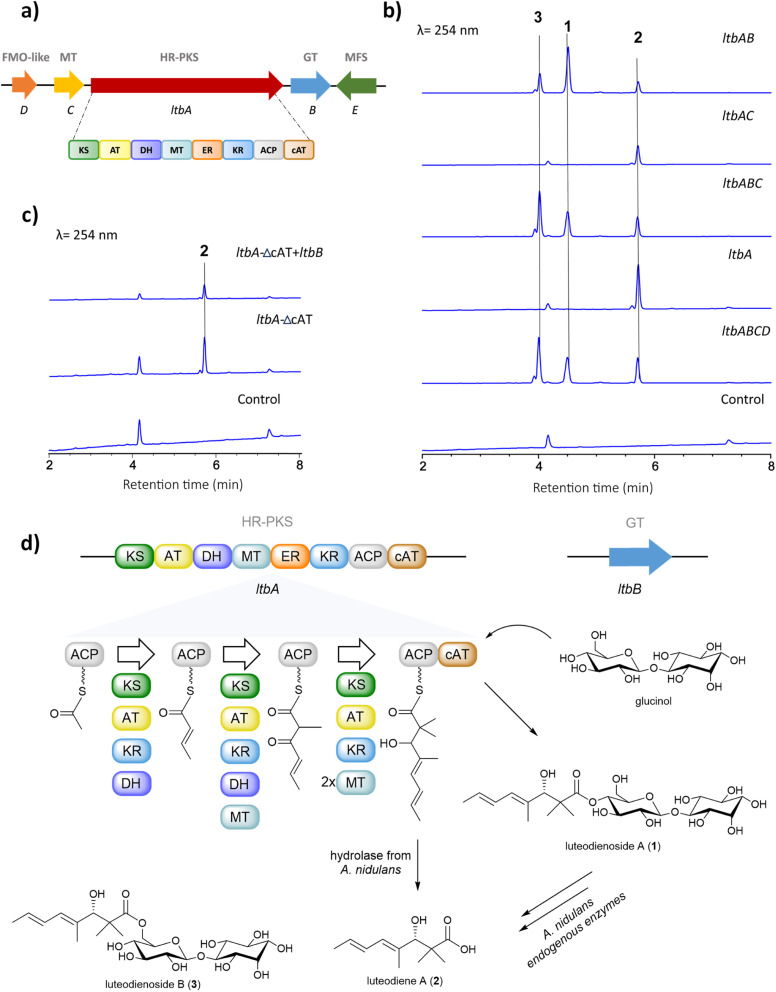
(a) Putative *ltb* BGC for the biosynthesis of 1. Abbreviations: HR-PKS, highly reducing polyketide synthase: MT, methyltransferase; FMO-like, flavin-dependent monooxygenase-like protein; GT, glycosyltransferase; MFS transporter, major facilitator superfamily. The domains of the HR-PKS: KS, ketosynthase; AT, acyltransferase; DH, dehydratase; MT, *C*-methyltransferase; ER, enoylreductase; KR, ketoreductase; ACP, acyl carrier protein; cAT, carnitine *O*-acyltransferase domains. (b) HPLC-DAD-MS profiling of *A*. *nidulans* LO8030 expressing various combinations of *ltb* genes; (c) HPLC-DAD-MS profiling of *A*. *nidulans* LO8030 expressing *ltbA*-ΔcAT and *ltbA*-ΔcAT + *ltbB*; (d) Proposed biosynthetic pathway to luteodienoside A (1) and pathway-specific products.

To identify the BGC for the biosynthesis of 1, we sequenced the genome of *A. luteorubrus* using Illumina HiSeq 2500. Subsequently, genome assembly and gene annotation were performed using the automatic assembly for the fungi (AAFTF) pipeline and the funannotate pipeline, respectively.^12,13^ The antibiotics and secondary metabolite analysis shell (antiSMASH) was used to predict BGCs within the genome of *A*. *luteorubrus*.^14^ The analysis identified two BGCs harbouring co-localised HR-PKS and glycosyltransferase genes in scaffolds 4.1 and 9.1. Notably, the BGC located on scaffold 4.1 encodes a HR-PKS (denoted LtbA) with a domain architecture containing ketosynthase (KS), acyltransferase (AT), dehydratase (DH), *C*-methyltransferase (MT), enoylreductase (ER), ketoreductase (KR), acyl-carrier protein (ACP) and carnitine *O*-acyltransferase (cAT) domains (Fig. 2a). HR-PKSs with C-terminal cAT domain fusions have been previously shown to produce polyketide chains with α,α-*gem*-dimethyl moieties,^15^ as observed in the polyketide chain of 1. This prompted us to focus on this BGC as the putative luteodienoside A BGC (*ltb*). In addition to LtbA, the enzymes encoded by the other genes within the *ltb* BGC include a glycosyltransferase-like family-2 protein (LtbB), a methyltransferase (LtbC), a flavin-dependent monooxygenase-like protein (LtbD) and a major facilitator superfamily (MFS) transporter (LtbE). The NCBI GenBank accession number for the *ltb* cluster is OR597289.

### Deciphering the biosynthetic pathway to luteodienoside A using heterologous expression reveals an unprecedented product offloading mechanism

After locating the candidate *ltb* BGC, we next sought to confirm its involvement in the biosynthesis of 1. To this end, we reconstructed the whole BGC in the heterologous host *Aspergillus nidulans* LO8030, which has been genetically modified to minimise endogenous metabolite background.^16^ The heterologous expression of the *ltb* BGC in *A*. *nidulans* was facilitated by the hybrid yeast-fungal artificial chromosome (pYFAC) expression system, which allows episomal expression of secondary metabolites in *A*. *nidulans*.^17,18^ We first cloned the *ltbA* gene into the vector pYFAC-*pyrG* using yeast transformation-associated recombination in *Saccharomyces cerevisiae*. Subsequently, *ltbB*, *ltbC* and *ltbD*, both together and individually, were cloned into the vector pYFAC-*ribO*. Next, *A*. *nidulans* LO8030 protoplasts were introduced with pYFAC plasmids carrying the gene combination *ltbABCD* to express the entire *ltb* BGC. LC-MS analysis of the *n*-butanol extract from the transformant confirmed the production of 1, along with two closely related compounds (2 and 3) that exhibited the same diene UV-vis profile as 1 (Fig. 2b and S6†). HRMS (Fig. S8†) and NMR (Table S6†) analysis of 2 identified the compound as the polyketide aglycone of 1, which we named luteodiene A. Similarly, HRMS (Fig. S8†) and NMR (Table S5†) analysis of 3 identified the compound as a regioisomer of 1, with the ester linkage between C-1 and C-6′ rather than between C-1 and C-4′. Collectively, these results demonstrated that the proposed *ltb* BGC is responsible for the biosynthesis of 1.

To characterise the biosynthetic logic of 1, various gene combinations were heterologously expressed in *A*. *nidulans* (Fig. 2b). Expression of *ltbA* alone resulted in the detection of 2, confirming it is the product of LtbA. Further EIC (+) *m*/*z* 505 analysis revealed that trace amount of 1 and 3 were present, but below the limit detectable by DAD (Fig. S9†). When *ltbABC* was expressed, peaks corresponding to 1, 2 and 3 were observed with no significant difference in metabolite production levels compared to *ltbABCD*, suggesting that *ltbD*, encoding a truncated flavin-dependent monooxygenase-like protein (Fig. S23 and S24†), is not essential for the biosynthesis of 1. To determine the roles of *ltbB* and *ltb*C, each gene was cloned both alone and co-expressed with *ltbA*. Expression of a transformant containing *ltbAC* resulted in the detection of carboxylic acid 2 by LC-MS analysis of a small-scale culture extract. Conversely, when the transformant harbouring *ltbAB* was heterologously expressed, 1, 2 and 3 were observed in the *n*-butanol extract. These results indicate that *ltbC*, despite encoding a methyltransferase containing the key active site residues (Fig. S22†), is not essential in the biosynthesis of 1, and that LtbA and LtbB are minimally required for the biosynthesis of 1 (Fig. 2b).

Interestingly, 2 and 3 were not observed in cultures of the native producer *A. luteorubrus*. To investigate possible influence of the heterologous host *A*. *nidulans* on the biosynthesis of 1, 2 and 3, compound 1 was fed to the *A*. *nidulans* LO8030 host at a concentration of 10 mg L^−1^. Interestingly, after 12 h of incubation, 2 and 3 were both observed in the media, suggesting that *A. nidulans* produces endogenous enzymes capable of hydrolysing 1 to 2 and isomerising 1 to 3 (Fig. S3†). To corroborate these results, we conducted co-expression of intron-free *ltbA* and *ltbB* in *S. cerevisiae* BJ5464-NpgA. However, this attempt did not result in the detection of 1 or any pathway-specific products, possibly due to LtbA and/or LtbB not being functionally expressed in *S. cerevisiae*.

### Generation of a cAT-truncated version of *ltbA* identifies glucinol as an offloading substrate for the LtbA cAT domain

The biosynthesis of α,α-*gem*-dimethylated polyketides has previously been shown to involve HR-PKSs with C-terminal-fused cAT domains. In *Trichoderma virens*, the Tv6-931 cAT domain can release the immature monomethylated products then recapture them back on the ACP to the MT domain to perform a second methylation cycle to generate α,α-*gem*-dimethyl-containing products.^15^ In contrast, the cAT domain of a HR-PKS from *Metarhizium anisopliae* (MaPKS17a) can catalyse the simple hydrolytic release of polyunsaturated polyketide products from the PKS.^19^ Other cAT domains have been previously identified in the BGCs of AF-toxin and sordarin, but their functions remain unclear.^20,21^ Although the final product of the *T*. *virens* BGC remains unknown, the cAT domain of the HR-PKS (Tv6-931) has been shown to utilise polyol nucleophiles to release the polyketide product as a polyol ester *in vitro*.^15^

To gain insights into the evolution and function of the LtbA-cAT domain, a phylogenetic analysis was constructed using amino acid sequences of 79 cAT domains extracted from various fungal HR-PKSs. The analysis revealed that the LtbA-cAT domain is distantly related to the known cAT domains (Fig. S2†), suggesting that it may have evolved a distinct function. We hypothesised that the LtbA-cAT domain may utilise glucinol as an offloading substrate to release the polyketide chain as a glucinol ester 1. To test this hypothesis, we generated a truncated version of LtbA (KS-AT-DH-MT-ER-KR-ACP) lacking the region encoding the cAT domain. This modified *ltbA* construct was heterologously expressed both alone and in combination with *ltbB* in *A*. *nidulans*. When expressing *ltbA*-ΔcAT alone, a peak corresponding to 2 was detected (Fig. 2c), suggesting that PKS product 2 could be released from the LtbA-ACP by endogenous hydrolases of *A*. *nidulans* or hydrolysed non-enzymatically. Likewise, when co-expressing *ltbA*-ΔcAT with *ltbB*, the transformant only accumulates 2 and not 1. These results suggest that (i) the LtbA-cAT domain is distinct from the previously characterised Tv6-931 cAT domain in that it is not involved in recapturing the elongated polyketide acyl chain for dimethylation and (ii) the cAT domain is involved in offloading the α,α-*gem*-dimethylated polyketide acyl chain from the ACP domain, likely using glucinol as a nucleophile, to yield the glucinol ester 1 (Fig. 2c). Examination of the active site residues in LtbA cAT, Tv6-931 cAT, and MaPKS17a cAT domains, alongside the authentic cAT, revealed conservation of the catalytic residue histidine and the serine residue involved in coordinating water molecule within the active site (Fig. S17†).

The presence of *gem*-dimethyl groups is relatively rare in fungal polyketides, with only one assigned MT domain from Tv6-931 having been identified to date. The α,α-*gem*-dimethylation catalysed by the MT domain of Tv6-931 requires the cAT domain to recapture the immature α-monomethyl polyketide acyl polyol ester chain for a second methylation cycle at the α position then release it again as the α,α-*gem*-dimethyl polyketide polyol ester. In contrast, the *M*. *anisopliae* MaPKS17a only produces monomethylated products, indicating that the MT domain lacks the ability to perform dimethylation. In our study, the product of *ltbA*-ΔcAT (*i.e.*2) has already undergone α,α-*gem*-dimethylation by the MT domain (Fig. S8†). This is significant as the absence of the cAT domain did not affect *gem*-dimethylation, indicating that the LtbA-MT domain can doubly methylate the α positions of the fully elongated polyketide acyl chain tethered on the ACP prior to its release, without being recaptured by the cAT domain (Fig. S16†).

To the best of our knowledge, this is the first report of a fungal cAT domain utilising a glycoside (glucinol) as the offloading substrate. This is in contrast to Tv6-931 cAT domain, which was shown to use short polyols, such as 1,1,1-tris(hydroxymethyl)ethane, pentaerythritol and glycerol, as releasing substrates.^15^ To validate the proposed function of the LtbA-cAT domain *in vitro*, we expressed the N-terminal His-tagged cAT domain in *Escherichia coli* Rosetta(DE3) (Fig. S12†). Additionally, we synthesised 3-hydroxy-2,2,4-trimethylocta-4,6-dienoic acid *N*-acetylcysteamine thioester (3-hydroxy-SNAC) as a surrogate substrate mimicking the ACP-*S*-phosphopantetheinyl (Ppant)-tethered 2. As glucinol was not readily available, we initially attempted to use its diastereomer, galactinol, as a substitute for the reaction (Fig. S7†). Various concentrations (10–50 μM) of the recombinant standalone cAT domain protein were incubated with 2 mM of galactinol and 2. However, the expected glycosylated product was not detected, possibly due to stringent substrate specificity of the LtbA-cAT domain. We next obtained authentic glucinol by hydrolysing 1 with 10 mM NaOH and repeated the reaction using the same conditions as for galactinol. However, the expected product was also not detected. This could be due to the cAT domain being unable to recognise the SNAC-tethered substrate. Thus, we attempted to assay the reverse transesterification activity of the *apo*-ACP-cAT domain of LtbA using an equimolar amount of 1 and free CoA or free SNAC as a mimic of the phosphopantetheinyl arm tethered on ACP (Fig. S13†). However, we could not detect the expected acyl-CoA or acyl-SNAC. This may suggest that unlike Tv6-931 PKS, which can catalyse reversible product release and recapture for double methylation, the *gem*-dimethyl moiety on 1 is produced by LtbA without recapture of the monomethylated polyketide product. Hence, the ability to catalyse the reverse reaction might not be inherent to LtbA.

Next, we performed a chemical complementation assay by feeding glucinol to a culture of *A*. *nidulans* expressing LtbA. After 24 h, the culture was extracted with *n*-butanol and analysed by LCMS and HRESI(+)MS (Fig. S14 and S15†). An extracted ion chromatogram (*m*/*z* 505) of the glucinol-fed culture revealed 7-fold enhancement of peaks corresponding to 1 and 3 as compared to a control culture that was not fed glucinol, providing further support for the hypothesis that LtbA-cAT uses glucinol as offloading substrate.

The glucinol moiety featured in 1 is unusual, hence we sought to characterise the proposed function LtbB. Amino acid sequence analysis of LtbB suggested that it is a transmembrane protein (Fig. S19 and S20†), so unsurprisingly, expression of the intron-free LtbB in *E*. *coli* yielded insoluble protein. Thus, cell-free lysate was prepared from *A. nidulans* expressing LtbB for the glycosylation assay. We incubated 2 mM UDP-glucose and 2 mM *myo*-inositol with the cell-free lysate in the presence of either Mg^2+^ or Mn^2+^. Subsequently, the reaction was analysed using gas chromatography-mass spectrometry. However, we were not able to detect the expected product, which could be due to the lack of the correct substrate for the LtbB.

Based on all the results above, we propose a pathway for the biosynthesis of 1 (Fig. 2d). The biosynthesis starts by loading of acetyl-CoA to the ACP domain. The KS domain then catalyses the polyketide extension with malonyl-CoA loaded by the AT domain, followed by ketoreduction and dehydration by the KR and DH domains, respectively, to generate the crotonyl diketide intermediate. When the triketide intermediate is formed, a methyl group is added by the MT domain. After another elongation cycle, the tetraketide intermediate undergoes two methylations to yield the mature *gem*-dimethylated polyketide chain tethered on the ACP. In parallel, LtbB uses nucleoside diphosphate (NDP)-glucose as a substrate to transfer d-glucose to *myo*-inositol to generate glucinol. Finally, the cAT domain releases the polyketide chain from ACP, using glucinol supplied by LtbB as a nucleophile to produce 1 (Fig. S18†).

### Comparative genomic analysis reveals wide distribution of *ltb* BGC homologues in the fungal kingdom

**Fig. 3:**
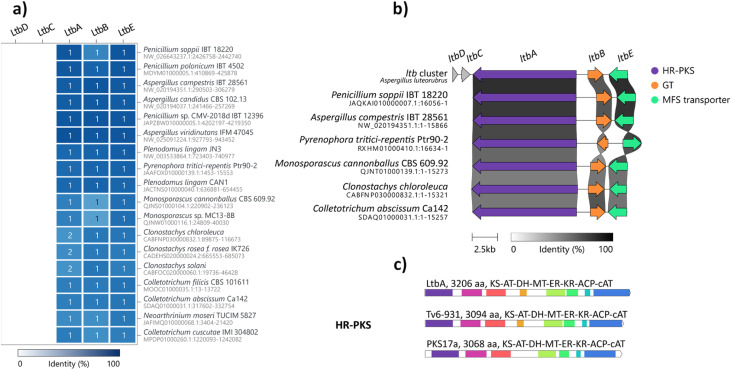
Bioinformatic analysis of the *ltb* BGC. (a) Representative homologous BGCs found in other fungi using cblaster with high conservation (≥48% identity across all genes). (b) BGCs homologous to the *ltb* BGC detected in other fungi aligned using clinker. (c) The domain architectures of the cAT domain-containing fungal HR-PKS, LtbA in *A*. *luteorubrus*, Tv6-931 in *T*. *virens* and PKS17a in *M*. *anisopliae*; KS, ketosynthase; AT, malonyl-CoA acyl transferase; DH, dehydratase; MT, methyltransferase; KR, ketoreductase; ACP, acyl carrier protein; cAT, carnitine *O*-acyltransferase.

Glycosylation is important in secondary metabolite biosynthesis, and glycosylated polyketides produced by fungi have a wide array of pharmaceutical applications. Numerous examples of fungal glycosylated polyketides have been review by Hussain and co-workers.^1^ In our study, the presence of a glucinol moiety in 1, along with its unique release mechanism, sparked our interest in exploring homologues of the *ltb* cluster in the fungal kingdom. A search of the NCBI GenBank database using the cblaster tool^22^ revealed homologues of the *ltb* BGC in various classes of fungi, including Eurotiomycetes, Sordariomycetes and Dothideomycetes (Fig. 3a and S21†). Selected homologous BGCs were aligned using clinker tool,^23^ revealing uncharacterised homologues of the *ltb* BGC in several other Aspergilli, including *Aspergillus candidus*, *Aspergillus campestris* and *Aspergillus viridinutans* (Fig. 3b). Of the five genes encoded in the *ltb* cluster, we found three genes to be conserved in almost all of the homologous BGCs, including genes encoding the HR-PKS LtbA, the NDP-glycosyltransferase LtbB and the transporter LtbE.

Interestingly, a homologue of the *ltb* BGC appears to be conserved in the plant pathogenic fungus *Pyrenophora tritici-repentis*. As we have access to a strain of *P. tritici-repentis* (WAC13651), we selected this BGC for heterologous expression to confirm the functions of these genes. Co-expression of the LtbA HR-PKS homologue (PtrM4_047710) and LtbB glycosyl-transferase homologue (PtrM4_047720) genes from *P. tritici-repentis* in *A*. *nidulans* resulted in the production of the same metabolites (*i.e.*1, 2 and 3) as those encoded by the *A. luteorubrus ltb* BGC (Fig. S5†). This confirmed that *P. tritici-repentis*, which belongs to a different class of fungi (Dothideomycetes) to *A. luteorubrus* (Eurotiomycetes), is capable of producing luteodienosides.

### Biological activity testing of metabolites

We screened 1, 2 and 3 for biological activity against the bacteria *Bacillus cereus* and *Staphylococcus aureus*, the fungi *Candida albicans* and *Saccharomyces cerevisiae*, and the plant *Eragrostis tef* (teff). However, no activity was observed for any of the metabolites up to 100 μg mL^−1^ (Table S11†).

## Conclusions

In conclusion, we have discovered a novel glycosylated polyketide luteodienoside A (1) and uncovered the molecular basis for its biosynthesis. Heterologous pathway reconstitution in *A. nidulans* led to 1 along with two additional products, luteodiene A (2) and luteodienoside B (3), not observed in the original fungus. In the heterologous *A. nidulans* host, the mature polyketide chain tethered on the ACP of LtbA can be hydrolysed to yield 2, while 1 can be regioisomerised to 3.

We have demonstrated that biosynthesis of the polyketide portion of 1 involves a novel HR-PKS LtbA capable of catalysing direct α,α-*gem*-dimethylation of the polyketide chain without requiring a product recapture step, in contrast to the previously characterised *T. virens* Tv6-931.^15^ This is supported by phylogenetic analysis, which showed that the LtbA-cAT domain formed separate clades to other characterised cAT domains, including *T*. *virens* Tv6-931. Significantly, by performing PKS truncation and chemical complementation, we have provided genetic and chemical evidence that the Ltb-cAT domain utilises glucinol as a substrate to offload and decorate the polyketide chain to yield 1. This represents the first report of a carbocyclic sugar-containing glycoside being used as an offloading nucleophile by a cAT domain of a fungal HR-PKS, thus expanding our understanding of the catalytic diversity of fungal HR-PKSs.

Additionally, we have identified an NDP-glycosyltransferase (LtbB) that is essential for the synthesis of the glucinol moiety present in 1. Although we were unable to obtain a functional recombinant enzyme to characterise its function *in vitro*, LtbB likely serves as a glucinol synthase by transferring d-glucose to *myo*-inositol using NDP-glucose as a substrate. While galactinol is widespread in plants and is known to serve as an intermediary galactosyl donor for the synthesis of raffinose-containing osmoprotectants,^24^ there are surprisingly few studies on its diasteromer, glucinol. One such study reported the synthesis of glucinol using enzyme extracts from the basidiomycete yeast *Sporobolomyces singularis*,^25^ while another study reported its isolation from the plants *Solanum tuberosum* and *Aegopodium podagraria*.^26^ A series of glucinol-derived glycolipids have also been isolated from *Solanum lanceolatum*, which were shown to exhibit anti-inflammatory activities.^27^

Genome mining revealed that homologues of the *ltb* BGC are conserved across multiple different classes of fungi, including many plant pathogens as well as mycoparasites. We showed that the homologous *ltb* BGC in the major wheat pathogen *P. tritici-repentis* also encodes the biosynthesis of 1. This wide distribution and the unusually high levels of production by *A*. *luteorubrus* (∼500 mg kg^−1^ grain) suggest an important ecological role for 1. Given the absence of any measurable antibacterial, antifungal or herbicidal activities for 1–3, this ecological role clearly warrants further investigation.

## Data availability

Additional experimental and characterisation data are available in the ESI.† Crystallographic data for compound 1 have been deposited at the Cambridge Crystallographic Data Centre (CCDC) under deposition number 2295166. The DNA sequence of the *ltb* cluster has been deposited NCBI GenBank with the accession number OR597289.

## Author contributions

A. A. A. performed the experiments and analysed the data. Z. S., H. L. and J. B. synthesised the substrate and revised the manuscript. A. C. and D. V. purified the compounds from the native strain and performed the bioassays. A. M. P. and Z. S. acquired NMR data and elucidated the structures. P. T. and W. L. obtained the crystal structure. G. R. F. acquired the experimental data from GCMS. Y. H. C., A. M. P. and E. L. supervised the study, acquired funding, and revised the manuscript. A. A. A. wrote the manuscript with input from all authors. All the authors have given approval to the final version of the manuscript.

## Conflicts of interest

There are no conflicts to declare.

## Supplementary Material

SC-015-D3SC05008D-s001

SC-015-D3SC05008D-s002
