# Supplementary material for: Discovery and heterologous biosynthesis of glycosylated polyketide luteodienoside A reveals unprecedented glucinol-mediated product offloading by a fungal carnitine O-acyltransferase domain
Source: Chem Sci. 2024 Jan 29;15(9):3349–56. doi: 10.1039/d3sc05008d (PMC10901484; doi:10.1039/d3sc05008d)
Supplement: SC-015-D3SC05008D-s001 [file SC-015-D3SC05008D-s001.pdf]

Electronic Supplementary Material (ESI) for Chemical Science.
This journal is © The Royal Society of Chemistry 2024

SUPPORTING INFORMATION

Discovery and heterologous biosynthesis of glycosylated polyketide luteodienoside A reveals unprecedented glucinol-mediated product offloading by a fungal carnitine *O*-acyltransferase domain

Amr A. Arishi [a,b], Zhuo Shang [a,c], Ernest Lacey [d,e], Andrew Crombie [e], Daniel Vuong [e], Hang Li [a,f], Joe Bracegirdle [a], Peter Turner [g], William Lewis [g], Gavin R. Flematti [a], Andrew M. Piggott [d *], Yit-Heng Chooi [a *]

[a.] *School of Molecular Sciences, The University of Western Australia, Perth, WA 6009, Australia*

[b.] *Department of Botany and Microbiology, College of Science, King Saud University, Riyadh 11451, Saudi Arabia*

[c.] *School of Pharmaceutical Sciences Shandong University Jinan, Shandong 250012, China*

[d.] *School of Natural Sciences, Macquarie University, Sydney, NSW 2109, Australia*

[e.] *Microbial Screening Technologies Pty. Ltd., Smithfield, NSW 2164, Australia*

[f.] *School of Pharmaceutical Sciences, Sun Yat-sen University, Guangzhou 510006, China*

[g.] *School of Chemistry, The University of Sydney, NSW 2006, Australia*

*Corresponding Authors:

Yit-Heng Chooi yitheng.chooi@uwa.edu.au

Andrew M. Piggott andrew.piggott@mq.edu.au

Table of Contents

Supplementary Experimental Procedures 3

1. Strains used in the study 4

2. Media and growth conditions 4

3. Luteodienoside A isolation from *Aspergillus luteorubrus* MST-FP2246 5

4. Small-scale metabolic profiling using LC-DAD-MS 6

5. Compounds isolation and structure elucidation 6

6. Structural elucidation of luteodiene A (**2**) and luteodienoside B (**3**) 6

7. Gas chromatography-mass spectrometry 7

8. X-ray crystallographic analysis 7

9. Primer design and *ltb* cluster sequence analysis 8

10. Phylogenetic analysis for fungal cAT domain 8

11. Phylogenetic analysis for LtbA-MT domain 8

12. Plasmids construction for heterologous expression of the *ltb* cluster 8

13. *A. nidulans* feeding experiment 9

14. Cell-free lysate assay of *A. nidulans ltbB* 9

15. RNA isolation and cDNA synthesis 10

16. Expression of *ltbA* and *ltbB* in *S. cerevisiae* BJ5464-NpgA 10

17. Overexpression of the standalone cAT domain and the ACP-cAT in *Escherichia coli* 11

18. Synthesis of luteodiene A-SNAC 12

19. Base-catalysed hydrolysis of luteodienoside A (**1**) to afford glucinol 12

20. cAT domain product offloading assay (SNAC) 13

21. Chemical complimentation assay 13

22. Bioactivity Screening 13

Supplementary Tables 15

Table S1. A list of primers used in the study 15

Table S2. DNA plasmids constructed in the study 16

Table S3. Expression strains used in the study 16

Table S4. Annotation of genes in the *ltb* cluster 17

Table S5. ^{1}H (600 MHz) and ^{13}C (150 MHz) NMR data for luteodienoside A (**1**) and luteodienoside B (**3**) in DMSO-d_6 18

Table S6. ^{1}H (600 MHz) and ^{13}C (150 MHz) NMR data for luteodiene A (**2**) in CD_3OD 19

Table S7. ^{1}H (600 MHz), ^{13}C (150 MHz) and 2D NMR data for glucinol in D_2O. 20

Table S8. Crystal data and structure refinement for luteodienoside A (**1**) 21

Table S9. Fractional atomic coordinates (×10^4) and equivalent isotropic displacement parameters (Å^2×10^3) for luteodienoside A (**1**). U_{eq} is defined as 1/3 of the trace of the orthogonalised U_{IJ} tensor. 22

Table S10. Anisotropic displacement parameters (Å^2×10^3) for luteodienoside A (**1**). The anisotropic displacement factor exponent takes the form: $-2\pi^2[h^2a^{*2}U_{11}+2hka^*b^*U_{12}+]$. 23

Table S11. Biological testing of compounds **1**–**3**. 24

Supplementary Figures 25

Figure S1. Olex2 depiction of luteodienoside A (**1**) with 50% displacement ellipsoids. 25

Figure S2. Phylogenetic tree for the cAT domains and the products of the corresponding HR-PKSs 26

Figure S3. LC-DAD-MS analysis of the *n*-butanol extract of *A. nidulans* LO8030 fed with 10 mg/L of luteodienoside A (**1**). 27

Figure S4. LC-DAD-MS analysis of the *n*-butanol extract of *S. cerevisiae* BJ5464-NpgA 28

Figure S5. LC-DAD-MS analysis of the *n*-butanol extract of *A. nidulans* expressing ltb cluster from *A. luteorubrus* and *Pyrenophora tritici-repentis* 29

Figure S6. UV-vis spectra of **1**–**3** obtained from LC−DAD analysis (5–95% MeCN/H_2O + 0.1% formic acid) 30

Figure S7. Structures of the sugar substrates used for *in vitro* experiments 31

Figure S8. High resolution mass spectrometry analysis (LC−OrbiTrap) of **1**–**3** ($^+$p ESI) of the molecules purified from *A. nidulans* 32

Figure S9. LCMS EIC(+) *m/z* 505 analysis of *A. nidulans* expressing *ltbA* showing the presence of traces for luteodienoside A (**1**) and luteodienoside B (**3**). 33

Figure S10. Schematic diagrams of constructed plasmids for the induced overexpression of *ltb* genes in the *A. nidulans* heterologous host 34

Figure S11. Protein sequences of the cAT. 35

Figure S12. SDS−PAGE (10%) of the semi-purified standalone cAT domain. 36

Figure S13. SDS−PAGE (10%) of the semi-purified ACP−cAT. 36

Figure S14. LCMS EIC(+) *m/z* 505 analysis of *A. nidulans* expressing *ltbA* fed with glucinol showing the presence of luteodienoside A (**1**) and luteodienoside B (**3**). 37

Figure S15. High resolution mass spectrometry analysis (LC−OrbiTrap) of *A. nidulans* strain expressing *ltbA* fed with glucinol showing the mass ($^{+}$p ESI) and the calculated molecular formulae of **1**. 38

Figure S16. Phylogenetic tree for the MT domains extracted from various HR-PKSs of fungi. The tree was constructed using Neighbour Joining method and 1000 bootstrap in MEGA-11. 39

Figure S17. Amino acid sequence alignment of the LtbA cAT domain with other fungi. 41

Figure S18. Proposed product offloading mechnaim by the LtbA-cAT domain. 43

Figure S19. 3D structural modelling of LtbB using AlphaFold. 44

Figure S20. 3D structure of LtbB by Phyre2 modelling and the top 4 homologous template proteins. 45

Figure S21. *ltb* cluster homologous BGCs found in NCBI database via cblaster search showing the upstream and the downstream boundary of the cluster.. 46

Figure S22. The result of the conserved domain search for the LtbC showing the catalytic residues of the enzyme. 47

Figure S23. The conserved domain analysis of the LtbD homologue in *A. brasiliensis* CBS 101740 (OJJ66157.1) showing the predicted function of the enzyme to be NAD(P)/FAD-dependent oxidoreductase 48

Figure S24. Pairwise protein alignment analysis of the LtbD and the homologue in *A. brasiliensis* CBS 101740 (OJJ66157.1). 48

Figure S25. ^{1}H NMR (600 MHz, DMSO-d_6) spectrum of luteodienoside A (**1**) 49

Figure S26. ^{13}C NMR (150 MHz, DMSO-d_6) spectrum of luteodienoside A (**1**) 49

Figure S27. HSQC NMR (600 MHz, DMSO-d_6) spectrum of luteodienoside A (**1**). 50

Figure S28. HMBC NMR (600 MHz, DMSO-d_6) spectrum of luteodienoside A (**1**) 50

Figure S29. ^{1}H-^{1}H COSY NMR (600 MHz, DMSO-d_6) spectrum of luteodienoside A (**1**) 51

Figure S30. ^{1}H-^{1}H ROESY NMR (600 MHz, DMSO-d_6) spectrum of luteodienoside A (**1**). 51

Figure S31. ^{1}H NMR (600 MHz, CD_3OD) spectrum of luteodiene A (**2**) 52

Figure S32. ^{13}C NMR (150 MHz, CD_3OD) spectrum of luteodiene A (**2**) 52

Figure S33. HSQC NMR (600 MHz, CD_3OD) spectrum of luteodiene A (**2**) 53

Figure S34. HMBC NMR (600 MHz, CD_3OD) spectrum of luteodiene A (**2**) 53

Figure S35. ^{1}H-^{1}H COSY NMR (600 MHz, CD_3OD) spectrum of luteodiene A (**2**) 54

Figure S36. ^{1}H NMR (600 MHz, DMSO-d_6) spectrum of luteodienoside B (**3**) 54

Figure S37. ^{13}C NMR (150 MHz, DMSO-d_6) spectrum of luteodienoside B (**3**) 55

Figure S38. HSQC NMR (600 MHz, DMSO-d_6) spectrum of luteodienoside B (**3**). 55

Figure S39. HMBC NMR (600 MHz, DMSO-d_6) spectrum of luteodienoside B (**3**). 56

Figure S40. ^{1}H-^{1}H COSY NMR (600 MHz, DMSO-d_6) spectrum of luteodienoside B (**3**) 56

Figure S41. ^{1}H NMR spectrum of glucinol in D_2O. 57

Figure S42. ^{1}H-^{1}H COSY NMR spectrum of glucinol in D_2O. 57

Figure S43. HSQC NMR spectrum of glucinol in D_2O. 58

Figure S44. HMBC NMR spectrum of glucinol in D_2O. 58

References 59

Supplementary Experimental Procedures

1. Strains used in the study

Aspergillus luteorubrus MST-FP2246 was isolated from a soil sample collected at White Mountains National Park in northern Queensland, Australia.[1] The heterologous expression host, *A. nidulans* strain LO8030 (a gift from Prof. Berl Oakley, University of Kansas) was used to express the *ltb* cluster. The characteristics of *A. nidulans* LO8030 have been described elsewhere.[2] *Pyrenophora tritici-repentis* strain WAC13651 was obtained from the Department of Agriculture and Food Western Australia (DAFWA). *Saccharomyces cerevisiae* strain BJ5464-NpgA (*MATα ura3-52 his3-Δ200 leu2-Δ1trp1 pep4::HIS3 prb1 Δ1.6R can1 GAL*) from Prof. Yi Tang, University of California, Los Angeles and JHY702 were used for plasmid construction and metabolites expression in yeast.[3,4] *Escherichia coli* NEB10-beta® electrocompetent cells were used to store and propagate the plasmids. *E. coli* Rosetta(DE3) (Novagen) was used for protein expression. All the strains were preserved in 25% (v/v) glycerol solution at −80 °C.

2. Media and growth conditions

For the growth of *A. luteorubrus* (MST-FP2246), recovery was made onto malt extract agar plates (MA) and grown for 7 days. The components of the MA plates are malt extract (20 g), glucose (20 g), bacteriological peptone (1 g) and bacteriological agar (20 g) in 1 L of H_2O with pH adjusted to 5.5 and sterilised at 121 °C for 30 min. Metabolite production was achieved by using agar squares from 7-day-old recovery plates to inoculate 40 × 250 mL Erlenmeyer flasks each containing 50 g of sterile basmati rice (40 mL water; 121 °C for 40 min). The flasks were incubated at 24 °C for 21 d.

For the growth of *A. nidulans*, glucose minimal medium (GMM) and stabilising medium (SMM) were used. The components of the GMM are glucose (10 g), $FeSO_4{\cdot}7H_2O$ (1.6 mg), $NaNO_3$ (6 g), $ZnSO_4{\cdot}7H_2O$ (22 mg), $CuSO_4{\cdot}5H_2O$ (1.6 mg), H_3BO_3 (11 mg), KCl (0.52 g), $MnCl_2{\cdot}4H_2O$ (5 mg), $MgSO_4{\cdot}7H_2O$ (0.52 g), $(NH_4)_6Mo_7O_{24}{\cdot}4H_2O$ (1.1 mg), $CoCl_2{\cdot}5H_2O$ (1.6 mg), K_2HPO_4 (1.52 g), Na_4EDTA (50 mg) in 1 L of H_2O and the pH was adjusted at 6.5. Other media components such as uracil/uridine, riboflavin and pyridoxine with suggested concentrations were supplemented if necessary.[5] SMM media was used for the growth of *A. nidulans* protoplast following transformation of the correct plasmids. The components of SMM media are the same as GMM media with the addition of 1.2 M sorbitol to stabilise the protoplast. *A. nidulans* was cultured at 37 °C unless stated.

For the growth of *S. cerevisiae*, yeast extract peptone dextrose (YPD) media was used. The components for YPD media are peptone (20 g), dextrose (20 g), yeast extract (10 g) and distilled water (1 L). After yeast transformation, *S. cerevisiae* was grown on drop-out media (Ura-). The components of the drop-out media are glucose (20 g), Bacto-yeast nitrogen base without amino acids (6.7 g), drop-out mix (1.4

g), His (76 mg), Trp (76 mg), Leu (380 mg), agar (20 g) and H_2O (1 L). Then, *S. cerevisiae* was incubated at 30 °C for 2–3 d and colonies were picked for validation.

For the growth of *E. coli* NEB10-beta ®, the lysogeny broth (LB) medium was used. The components of LB media are yeast extract (5 g), NaCl (10 g), tryptone (10 g), ampicillin (0.05 g/L) and deionised water (1 L). The incubation condition for *E. coli* was at 37 °C for overnight.

3. Luteodienoside A isolation from *Aspergillus luteorubrus* MST-FP2246

Agar squares taken from 7-day-old Petri plates of MST-FP2246 were used to inoculate 40 × 250 mL Erlenmeyer flasks each containing 50 g of sterile basmati rice (40 mL water; 121 °C for 40 min). The flasks were incubated at 24 °C for 21 d, after which the grains were pooled and extracted with acetone (5 L) and dried *in vacuo* to give an aqueous residue (600 mL). The residue was then partitioned against ethyl acetate (2 × 1 L) and dried *in vacuo* to give an organic extract (6.7 g) and an aqueous fraction (600 mL). The aqueous fraction was loaded onto C_{18} resin (Hypersil, 100 g) and washed sequentially with 300 mL volumes of H_2O, 25% aqueous MeOH, 50% aqueous MeOH and 100% MeOH. The 100% MeOH fraction was dried *in vacuo* to give an enriched extract (3.0 g), which was adsorbed onto silica gel (10 g) and loaded onto a silica gel column (100 g, 300 × 50 mm). The column was washed once with hexane, then eluted with 50% hexane/$CHCl_3$, 25% hexane/$CHCl_3$ and 100% $CHCl_3$, followed by a stepwise gradient of 1, 2, 4, 8, 16, 32 and 100% MeOH/$CHCl_3$ (500 mL each step), to yield 11 fractions (Fr 1–11).

A sub-sample of Fr 11 (100 mg) was dissolved in MeOH (2 mL) and fractionated using a size exclusion chromatography column (25 × 300 mm) of Sephadex LH-20 resin (Davisil, Grace Discovery) in MeOH with fractions (4 mL) collected at a flow rate of 4 mL min^{-1}. The fractions were sub-sampled and analysed by C_{18} analytical HPLC. Fractions 7–14 were pooled and evaporated to give an enriched fraction (79.0 mg). The enriched fraction was purified by isocratic preparative HPLC (Alltima C_{18}, isocratic 25%/25%/50% MeCN/MeOH/H_2O, 20 mL min^{-1}) to yield luteodienoside A (**1**) (t_R 8.02 min; 22.1 mg).

Analytical HPLC was performed on a gradient Agilent 1260 Infinity quaternary HPLC system. The column was an Agilent Zorbax SB-C_{18} (2.1 × 50 mm; 1.8 μm) eluted with a 0.6 mL min^{-1} gradient of 10–100% MeCN/water (0.01% TFA) over 11 min. Preparative HPLC was performed on a gradient Shimadzu HPLC system comprising two LC-8A preparative liquid pumps with static mixer, SPD-M10AVP diode array detector and SCL-10AVP system controller with standard Rheodyne injection port. The column used in the purification of the metabolites was an Alltima C_{18} column (22 × 250 mm; 5 µm; Grace Discovery) eluted isocratically at 20 mL min^{-1}, eluted isocratically with 25%/25%/50% MeCN/MeOH/H_2O.

4. Small-scale metabolic profiling using LC-DAD-MS

Secondary metabolites analysis was performed for the *A. nidulans* containing the *ltb* cluster using Agilent Infinity 1260 liquid chromatography system incorporated with a diode array detector and an Agilent 6130 single quadrupole system (LC-DAD-MS) alongside ESI source and Kinetex C_{18} column (100 Å; 100 mm × 2.1 mm). For small-scale analysis, approximately 10^8/L of the spores were harvested from 4-days-old fungal culture and inoculated onto 50 mL of GMM media. The culture was grown for 18 h at 37 °C/180 rpm, then supplemented with cyclopentanone (2.5 mL/L) to induce the expression of the alcohol-inducible promoters regions.[6] Following this, the culture was incubated for another four days at 25 °C/180 rpm to accumulate the metabolites. Acetone was used to extract the mycelia, and *n*-butanol was used to extract the liquid media. The crude extracts were dried down and redissolved in 500 µL of MeOH for the LC-DAD-MS analysis. For chromatographic analysis, a gradient of 5–95% MeCN/H_2O (0.1% formic acid) was used for 10 min at a flow rate of 0.6 mL min^{-1}. The data generated by MS were collected at a positive mode range of *m/z* 100–1000.

5. Compounds isolation and structure elucidation

For large-scale production of the secondary metabolites, the *A. nidulans* containing the *ltb* cluster was grown in 8 L of GMM media in shake-flask culture (with appropriate supplements) under the same condition described above. Then, a Reveleris flash chromatography system (Grace) was used to fractionate the crude extract using MeOH/H_2O gradients (20% MeOH/H_2O to 100% MeOH with 0.1% formic acid, 40 mL min^{-1}) on a Reveleris C_{18} flash cartridge. The system uses a UV detector alongside with evaporative light scattering detector. LC-DAD-MS was used to identify the fractions that contains the target compound, which was combined for further purification using semi-prep HPLC with a C_{18} column (Phenomenex; 5 µm; 21.2 × 150 mm). After purification, 300 mg, 36 mg and 25 mg of compounds **1**, **2** and **3**, respectively, were obtained. ^{1}H, ^{13}C and 2D nuclear magnetic resonance (NMR) spectra were acquired using a Bruker AV 600 spectrometer.

6. Structural elucidation of luteodiene A (2) and luteodienoside B (3)

HRESI(+)MS analysis of **2** revealed a dehydrated protonated ion at *m/z* 181.1220 $[M - H_2O + H]^+$, indicative of a molecular formula $C_{11}H_{18}O_3$ (Δmmu −0.3) requiring three double bond equivalents (DBEs). Analysis of 1D NMR (CD_3OD) data of **2** revealed four sets of methyl protons ($\delta_{H\text{-}8}$ 1.77, $\delta_{H\text{-}9}$ 1.12, $\delta_{H\text{-}10}$ 1.13, $\delta_{H\text{-}11}$ 1.73), three olefinic protons ($\delta_{H\text{-}5}$ 5.98, $\delta_{H\text{-}6}$ 6.29, $\delta_{H\text{-}7}$ 5.68), one oxygenated methine ($\delta_{H\text{-}3}$ 4.22), and one carbonyl group ($\delta_{C\text{-}1}$ 181.2). A substructure CH_3-CH=CH=CH contributing two DBEs was established based on the sequential ^{1}H-^{1}H COSY correlations. Moreover, the large coupling constant $^3J_{H\text{-}6/H\text{-}7}$ (14.0 Hz) and a ROESY correlation between H-11 and H-6 suggested an *E* configuration for the alkene. The key HMBC correlations from H-11 to C-3 and C-5, and from H-9/H-10 to C-1, C-2 and C-3 permitted the assignment of a substructure 3-hydroxy-2,2,4-trimethylpent-4-enoic acid as shown in (**Table S6**). The presence of a carboxylic group ($\delta_{C\text{-}1}$ 181.2) in **2** was deduced

by the analysis of HRMS and ^{13}C NMR data, which contributed the remaining DBE. Thus, **2** was identified as (4*E*,6*E*)-3-hydroxy-2,2,4-trimethylocta-4,6-dienoic acid, which we named luteodiene A.

HRESI(+)MS analysis of **3** revealed a dehydrated protonated ion at *m/z* 505.2274 $[M - H_2O + H]^+$, indicative of a molecular formula $C_{23}H_{38}O_{13}$ (Δmmu −0.6), which is isomeric with **1**. The 1D NMR (DMSO-d_6) data for **3** are very similar to those for **1**, with the only significant differences being the downfield shift of C-6′ ($\Delta\delta_C$ +2.7) and upfield shift of H-4′ ($\Delta\delta_H$ −1.43). HMBC analysis of **3** indicated a C-1−O−C-6′ connection between the olefinic chain and the *β*-D-glucose moiety instead of C-1−O−C-4′ bridge in **1** (**Table S5**). The absolute configurations of both hexose moieties were tentatively assigned to be the same as those in **1** based on the biosynthetic considerations. Thus, **3** was elucidated to be a regioisomer of luteodienoside A, which we named luteodienoside B.

7. Gas chromatography-mass spectrometry

To 0.2 mg of the dried reaction mixture, dry pyridine (100 µL; distilled from CaH_2 and stored over anhydrous KOH) and *N*,*O*-bis(trimethylsilyl)trifluoroacetamide with 1% trimethylchlorosilane (100 µL; Merck, p/n 15238) were added to the vials. The vials were then placed on a heating block at 60 °C for 30 min. The resulting samples were analysed directly using gas chromatography-mass spectrometry (GC-MS). GC-MS was conducted using an Agilent 6890 GC connected to an Agilent 5973 mass-selective detector (Agilent Technologies, USA). Separation was achieved using a BPX-5 column (5% phenyl polysilphenylene– siloxane, 30 m × 0.25 mm internal diameter × 0.25 mm film thickness, SGE analytical science), with ultra-high purity helium as the carrier gas at a constant flow rate of 1 mL/min. One µL of samples were injected in splitless mode with the inlet temperature set to 280 °C. The initial oven temperature was set at 50 °C and then ramped at 10 °C/min to 280 °C and held for 10 min. The ion source was set at 200 °C, and the spectrometer was set to record between 45 and 600 amu following a 10 min solvent delay.

8. X-ray crystallographic analysis

Single crystals of luteodienoside A (**1**) were obtained by slow evaporation of a solution of **1** in MeOH. A suitable crystal was selected and attached to a nylon loop with Exxon Paratone N, then quenched in a cold nitrogen gas stream from an Oxford Cryosystems Cryostream. Data were collected on a SuperNova, Dual, Cu at home/near, Atlas diffractometer. The crystal was kept at 100(2) K during data collection. Using Olex2,[7] the structure was solved with the olex2.solve [8] structure solution program using Charge Flipping and refined with the SHELXL[9] refinement package using Least Squares minimisation. Crystallographic data (summarised in Tables S8–S10) have been deposited in the CCDC and are available with deposition number 2295166.

Crystal Data for $C_{23}H_{46}O_{17}$ (M =594.60 g/mol): orthorhombic, space group $P2_12_12_1$ (no. 19), a = 6.60006(18) Å, b = 7.7434(3) Å, c = 55.3300(13) Å, V = 2827.75(16) Å^3, Z = 4, T = 100(2) K, μ(Cu Kα) = 1.028 mm^{-1}, $Dcalc$ = 1.397 g/cm^3, 53625 reflections measured (9.59° ≤ 2Θ ≤ 155.984°), 5946 unique (R_{int} = 0.0667, R_{sigma} = 0.0303) which were used in all calculations. The final R_1 was 0.0528 (I > 2σ(I)) and wR_2 was 0.1013 (all data).

9. Primer design and *ltb* cluster sequence analysis

Geneious Prime® (version 2021) was used for primer design and sequence data analysis (http://www.geneious.com). The cblaster tool was used to search for co-localised genes on the NCBI database to identify homologous gene clusters in other fungi and to extract the nucleotide sequences of the detected clusters.[10] clinker was used to generate a gene cluster comparison plot to allow a comparison of the detected clusters.[11]

10. Phylogenetic analysis for fungal cAT domain

To investigate whether the HR-PKS cAT domain of the *ltb* cluster differs from previously examined cAT domains, we used the fungal strains from the phylogeny proposed by Hang et al.[12] The amino acid sequences of the fungal cAT domains were identified and extracted from HR-PKSs using BLASTp searches of homologous protein sequences,[13] and the Conserved Domain Database.[14] MEGA-11 was used to construct a Neighbour Joining tree.[15] The sequences of the cAT domains were aligned using ClustalW alignment tool, which is built-in MEGA-11 software. The parameters of the phylogenetic analysis were similar to that used by Hang et al.,[12] including the gap opening penalty = 10 and gap extension penalty = 0.1. To ensure the reproducibility of the phylogeny, 1000 bootstrap replicates were used and *p-distance* Model.

11. Phylogenetic analysis for LtbA-MT domain

To perform phylogenetic analysis, we extracted the amino acid sequence of the LtbA-MT domain and performed a BLASTp search to find homologous protein sequences and the Conserved Domain Database.[13,14] MEGA-11 was used to construct a Neighbour Joining tree.[15] The same parameters described above were used to construct the phylogenetic tree the LtbA-MT domain.

12. Plasmids construction for heterologous expression of the *ltb* cluster

Two yeast-fungal artificial chromosomes (pYFAC) containing the auxotrophic marker for uracil/uridine (*pyrG*) and riboflavin (*riboB*) were used in the study. PacI, NotI and AsiSI endonucleases were used to digest the plasmids for cloning, as recommended by the manufacturer. Gene components of the *ltb* cluster, *ltbA*, *ltbB*, *ltbC* and *ltbD*, were amplified from the genomic DNA of *Aspergillus luteorubrus* MST-FP2246 using Q5 high-fidelity DNA polymerase (New England Biolabs, MA, USA)

with 30–40 nucleotide overhang regions homologous to the ends of the cloning sites of the vector using the primers listed in Table S1, then introduced under the control of alcohol-inducible promoters: *alcA*, *alcS*/*M*, *aldA* via yeast-mediated recombination using the overlapping DNA fragments.

The *ltbA* gene was amplified using three sets of primers listed in Table S1 and cloned into PacI-digested pYFAC-*pyrG* to generate pYFAC-CL50. Other genes, *ltbB*, *ltbC* and *ltbD*, were amplified with overhangs and cloned into PacI, NotI, AsisI sites of pYFAC-*riboB*, to generate pYFAC-CL51-54 (as listed in Table S2). Diagnostic PCR was used to screen for the correct yeast transformants. A single correct yeast colony was cultivated in yeast drop-out media (3 mL) at 30 °C, 200 rpm for 2 days. Then, plasmid extraction from yeast was performed with Zymoprep™ Yeast Plasmid Miniprep I Kit. 1 μL of the yeast miniprep was used for *E. coli* NEB10-beta® electroporation. Plasmids were screened using diagnostic PCR followed by digestion analysis. Correct plasmids were transferred to *A. nidulans* protoplast using polyethylene glycol-mediated transformation (PEG) as described previously.[5] Correct colonies were selected on SMM agar supplemented with appropriate growth factors (uracil/uridine, riboflavin and pyridoxine) as required. Transformants with different combinations were constructed and selected for small-scale heterologous expression as shown in Table S3. Empty plasmids were transferred to *A. nidulans* LO8030 and used as a control.

13. *A. nidulans* feeding experiment

A feeding experiment was performed using luteodienoside A (**1**) as a substrate. A non-transformant *A. nidulans* was streaked on GMM agar plates with the required supplements, then the fungus was inoculated into 50 mL of GMM broth media and incubated for 18 h under the same conditions described above to accumulate biomass. Subsequently, **1** was dissolved in MeOH and fed to the *A. nidulans* at a concentration of 10 mg/L. As a control, **1** was incubated in GMM media under the same conditions. After two days of incubation, the media were extracted with 50 mL of *n*-butanol, dried under N_2 gas and dissolved in MeOH for LC-DAD-MS analysis.

14. Cell-free lysate assay of *A. nidulans ltbB*

To prepare cell-free lysate, a plasmid harbouring *ltbB* was introduced to *A. nidulans*. Then, the transformant was inoculated into 50 mL GMM broth media with the required supplements. Subsequently, the culture was incubated for 18 h under the same conditions described above and induced with cyclopentanone to allow the expression of LtbB, following by incubation for 3 days at 25 °C. Mycelia were harvested, frozen with liquid nitrogen and ground into fine powder with a mortar and pestle. The powder was resuspended in lysis and equilibration buffer (50 mM NaH_2PO_4, 300 mM NaCl, 10 mM imidazole) supplemented with 2 mM DTT, and lysed by sonication on ice. Subsequently, the cell lysate was centrifuged at 17000 rpm, 4 °C for 40 min to remove the cellular debris and the

supernatant was used for the assay. Cells-free lysate was also prepared using *A. nidulans* transformed with empty plasmid and used as control.

The assay was performed in a 200 µL system. For the reaction, 150 µL of the cell-free lysate was incubated with 2 mM of UDP-glucose and 2 mM *myo*-inositol in the presence of Mg^{2+} or Mn^{2+} at 28 °C overnight. Subsequently, the reaction tube was dried under a stream of nitrogen and prepared for the analysis by gas chromatography-mass spectrometry (GCMS). A non-transformant *A. nidulans* was used as control for the experiment.

15. RNA isolation and cDNA synthesis

Total RNA was extracted from *ltbAB*-overexpressing *A. nidulans* and used as a template for cDNA synthesis using mini hot phenol extraction method described previously.[16] Briefly, the mycelium of *A. nidulans ltbAB* was grown on 50 mL at 37 °C for 18 h, then cyclopentanone (2.5 mL/L) was added to induce the expression of *ltbAB*. The mycelia were incubated at 25 °C for one day, then harvested, frozen with liquid nitrogen and ground to fine powder with a mortar and pestle. The powder was transferred to hot phenol buffer consisting of 250 µL of saturated phenol, 500 µL of homogenisation buffer (100 mM Tris-HCl, 5 mM EDTA, 100 mM NaCl, 0.5% SDS, pH 7.5), and 5 µL of *β*-mercaptoethanol, followed by agitation for 15 min. Chloroform (250 µL) was added, and the sample was shaken for another 15 min, followed by centrifugation (4000 × *g*) at room temperature for 10 min. Then, 550 µL of phenol-chloroform (1:1) mixture was used to extract the aqueous phase twice. Subsequently, 3 M sodium acetate (0.1 volumes) and isopropanol (0.8 volumes) were added to the aqueous phase, followed by incubation for 15 min at −20 °C to allow the precipitation of the RNA. The precipitate was pelleted by centrifuging the sample for 30 min at 4 °C. The pellet was air dried then suspended in 500 μL RNase-free water. An equal volume of 4 M LiCl was added to the sample followed by overnight incubation at 4 °C. During the incubation, a precipitate started to form, which was pelleted by centrifugation (4000 × *g*) at 4 °C for 30 min. The pellet was washed with 1 mL aqueous ethanol (80%), dried and resuspended in 50 μL RNase-free water.

To clean the extracted RNA from contamination with genomic DNA, the sample was treated with deoxyribonuclease. Briefly, 5 µL of extracted RNA was incubated with 1 µL of deoxyribonuclease I (DNase I, 1 unit/µL, Thermo Fisher) and 1 µL of the reaction buffer (200 mM Tris-HCl, 20 mM $MgCl_2$, pH 8.3) and at room temperature for 15 min. To terminate the reaction, 1 µL of stop solution (50 mM EDTA) was added and heat at 70 °C for 10 min. Following this, 4 µL of the treated RNA was directly used as the template for cDNA synthesis using LunaScript RT SuperMix Kit (NEB) as per the manufacturer's instructions.

16. Expression of *ltbA* and *ltbB* in *S. cerevisiae* BJ5464-NpgA

The intron-free *ltbA* and *ltbB* were amplified from cDNA using the primers listed in Table S1. The 2µ-based yeast expression vector pXW55 and pXP7418 were used to express *ltbA* and *ltbB.* To prepare the

plasmid for cloning, the pXW55 vector was digested with the two endonucleases SpeI and PmlI and used to as a vector for *ltbA* expression. Similarly, pXP7418 was digested with the two endonucleases SpeI and XhoI and used as a vector for *ltbB* expression. Subsequently, *S. cerevisiae* strain BJ5464-NpgA was used to clone the PCR products to the linear vector to generate pXW55-*ltbA* and pXP741-*ltbB*, respectively. Yeast colonies were screened, and correct colonies were grown on yeast drop-out media (3 mL) at 30 °C. Correct plasmids were extracted and transferred to *E. coli* NEB10-beta® for plasmid propagation. Plasmids were validated by digestion analysis then sequenced using Illumina MiSeq sequencing.

After plasmid validation, the two plasmids, pXW55-*ltbA* and pXP741-*ltbB*, were transferred to *S. cerevisiae* BJ5464-NpgA to screen for metabolite production. Firstly, pXW55-*ltbA* was transferred alone to *S. cerevisiae* BJ5464-NpgA (*S. cerevisiae ltbA*) to check for the production of **2**. A seed culture of the *ltbA*-expressing yeast was inoculated into a yeast drop-out media (3 mL) and incubated at 30 °C/100 rpm for two days, then used to inoculate 2 L of YPD culture and incubated at 30 °C/180 rpm for four days. In the same manner, pXW55-*ltbA* and pXP741-*ltbB* were transferred to *S. cerevisiae* BJ5464-NpgA together (*S. cerevisiae ltbAB*) to check for the production of **1**. A seed culture of the transformant yeast was inoculated into 2 L of YPD culture and incubated under the same condition described above. Acetone was used to extract the cells, and *n*-butanol was used to extract the liquid media. The crude extracts were dried down under N_2 gas and redissolved in 500 µL of MeOH for the LC-DAD-MS analysis.

17. Overexpression of the standalone cAT domain and the ACP-cAT in *Escherichia coli*

The corresponding intron-free cAT domain and the ACP-cAT were amplified from the cDNA using the primers listed in Table S1. The PCR products of the cAT domain and the ACP-cAT were assembled to the digested vector pET-28a (digested with NdeI and NheI) using Gibson assembly. Subsequently, 1 µL of the resulted recombinant plasmids were transferred to *E. coli* NEB10-beta® for plasmid propagation. Correct colonies were confirmed by sequencing and transferred to *E. coli* -Rosetta(DE3) competent cells for protein expression. A seed culture of the transformant *E. coli* was grown in 3 mL of LB media supplemented with kanamycin (50 µg/mL) for overnight at 37 °C/ 100 rpm. Then, 1% of the seed culture was inoculated into 1 L of LB media supplemented with kanamycin (50 µg/mL) at 37 °C to an OD_{600} of 0.6–0.8. To induce the protein expression, isopropyl-β-D-thiogalactopyranoside (IPTG) was add to a final concentration of 0.2 mM. The induced culture was shaken further for 19 h at 16 °C/100 rpm. The culture was harvested by centrifugation at 5000 × *g* for 15 min and resuspended in binding buffer (20 mM Tris-HCL, 100 mM NaCl, 20 mM imidazole, pH 7.8). The harvested cells were lysed by sonication on ice, followed by centrifugation at 14000 rpm at 4 °C for 1 h to remove the cellular debris. To check protein solubility, the supernatant was analysed using 10% sodium dodecyl sulfate–polyacrylamide gel electrophoresis (SDS-PAGE). Spectra™ Multicolor Broad Range Protein Ladder

was used as a protein marker, subsequently the SDS-PAGE was stained using InstantBlue® Coomassie Protein Stain (ISB1L).

To purify the overexpressed enzymes, the lysates with the soluble proteins were filtered with 0.45 µm filters and loaded into a HisPur™ Ni–NTA affinity column (Thermo Scientifc, Rockford, USA) following the manufacturer protocol. After loading the filtered lysates into the column, the recombinant proteins were eluted using elusion buffer (20 mM Tris-HCL, 100 mM NaCl, pH 7.8) with different concentrations of imidazole (20 mM, 50 mM, 100 mM, 200 mM, 300 mM). The protein fractions were validated using SDS-PAGE. The proteins were eluted at the concentration of 100 mM and 200 mM of imidazole). For each protein, the corresponding fractions were pooled together. The pooled fractions were concentrated and exchanged into storage buffer (20 mM Tris-HCl, 100 mM NaCl with pH 7.8) with a 10 K Macrosep Advance Centrifugal Device (Pall Corporation, New York, USA). The concentration of the protein was measured with a Thermo Scientific NanoDrop 2000 Spectrophotometer. The protein concentrations were 59.5 mg/mL and 10.2 mg/mL for the cAT domain and ACP-cAT, respectively. The enzymes were stored in 10% (v/v) glycerol at −80 °C until the time of the reaction. All protein purification steps were performed at 4°C.

18. Synthesis of luteodiene A-SNAC

The luteodiene A-SNAC was synthesised using a method described previously.[17] Briefly, a solution of luteodiene A (**2**; 10 mg, 50.5 µmol), diisopropylethylamine (DIPEA; 10.6 µL, 60.6 µmol), *N*-(3-dimethylaminopropyl)-*N*-ethylcarbodiimide hydrochloride (EDC; 11.6 mg, 60.6 µmol), hydroxybenzotriazole (HOBt; 8.2 mg, 60.6 µmol) and *N*-acetylcysteamine (6.5 µL, 60.6 µmol) in CH_2Cl_2 (5 mL) was stirred at room temperature for 12 h, then dried *in vacuo*. The resulting residue was dissolved in MeOH (1 mL) and purified by semi-preparative HPLC (Grace Hypersil C_{18} column, 5 µm, 250 × 10 mm; 4.0 mL/min; isocratic 55% H_2O/MeCN with 0.05% TFA over 25 min), yielding luteodiene A-SNAC (t_R 6.13 min; 0.8 mg isolable yield). HRESI(+)MS *m/z* 282.1518 $[M - H_2O + H]^+$, calcd. for $C_{15}H_{24}NO_2S^+$ 282.1522 $[M - H_2O + H]^+$.

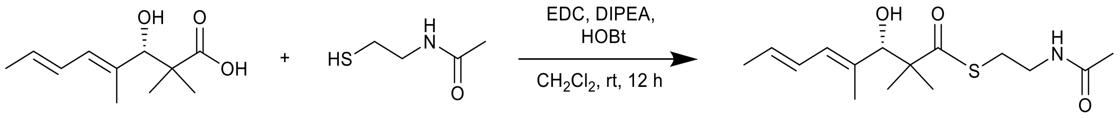

19. Base-catalysed hydrolysis of luteodienoside A (1) to afford glucinol

Luteodienoside A (**1**; 10 mg) was dissolved in 5% MeOH in H_2O (0.5 mL) and aqueous NaOH (2 M; 0.5 mL) was added. The reaction mixture was incubated for 16 h at 40 °C, then neutralised by addition of HCl (2 M; 1 mL). The mixture was then extracted twice with same volume of ethyl acetate, dried under N_2 gas, resuspended in 5% MeOH in H_2O (1 mL) and passed through a C_{18} SPE column multiple

times following dilution of the eluate 1:1 with H_2O. Finally, the SPE column was eluted with 5% MeOH in H_2O (5 mL) and the eluate was dried using N_2 gas, yielding approximately 3 mg of glucinol.

20. cAT domain product offloading assay (SNAC)

The enzymatic assay was performed using various concentrations of the standalone cAT domain (10-50 µM) enzyme. The reaction mixture consisted of 2 mM of galactinol or glucinol and 2 mM SNAC of **2** in 20 mM Tris-HCl or 10 mM NaH_2PO_4 buffer (pH 7.8). Total reaction volume used for the assay was 200 µL. The reaction mixture was incubated at 28 °C for 24 h then diluted with three volumes of MeCN and centrifuged at maximum speed for 8 min. The supernatant was analysed using LC-DAD-MS.

21. Chemical complimentation assay

To perform the chemical complementation assay, *ltbA*-expressing *A. nidulans* was inoculated into 50 mL culture medium and the culture was incubated for 18 h at 37 °C. Cyclopentanone (2.5 mL/L) was added to induce expression of *ltbA* and the culture was incubated for 8 h at 25 °C. Glucinol (2 mg) was dissolved in 200 µL sterilised water and added to the culture and incubation was continued for 24 h at 25 °C. The culture was extracted with 50 mL of *n*-butanol and dried under N_2 gas. The residue was redissolved in MeOH (200 µL) and analysed by LC-DAD-MS.

22. Bioactivity Screening

Purified metabolites were dissolved in DMSO to provide stock solution of 10,000 µg/mL. An aliquot of each stock solution was transferred to the first lane of Rows B to G in a 96-well microtitre plate and two-fold serially diluted with DMSO across the 12 lanes of the plate to provide a 2,048-fold concentration gradient. Bioassay medium was added to an aliquot of each test solution to provide a 100-fold dilution into the final bioassay, thus yielding a test range of 100 to 0.05 µg/mL in 1% DMSO for majority of our test organisms, and 200 to 0.1 µg/mL in 2% DMSO for yeasts. Row A contained no test compound (as a reference for no inhibition) and Row H was uninoculated (as a reference for complete inhibition).

ProTOX is a bioassay platform for antibiotic discovery. In the present study *Bacillus subtilis* (ATCC 6633) and *Staphylococcus aureus* (ATCC 25923) were used as indicative species for antibacterial activity, respectively. A bacterial suspension (50 mL in 250 mL flask) was prepared in nutrient media by cultivation for 24 h at 250 rpm, 28 °C. The suspension was diluted to an absorbance of 0.01 absorbance units per mL, and 10 µL aliquots were added to the wells of a 96-well microtitre plate, which contained the test compounds dispersed in 190 µL nutrient broth (Amyl) with 10 µL resazurin (12.5 µg/mL). The plates were incubated at 28 °C for 48 h during which time the positive control wells change colour from a blue to light pink colour. MIC end points were determined visually. The

absorbance was measured using Multiskan Skyhigh Microplate Spectrophotometer (Thermofisher Scientific) at 605 nm.

EuTOX is a bioassay platform for antifungal discovery. In the present study, the yeasts *Candida albicans* (ATCC 10231) and *Saccharomyces cerevisiae* (ATCC 9763) were used as indicative species for antifungal activity. A yeast suspension (50 mL in 250 mL flask) was prepared in 1% malt extract broth by cultivation for 24 h at 250 rpm, 24 °C. The suspension was diluted to an absorbance of 0.005 and 0.03 absorbance units per mL for *C. albicans* and *S. cerevisiae*, respectively. Aliquots (20 µL and 30 µL) of *C. albicans* and *S. cerevisiae*, respectively were applied to the wells of a 96-well microtitre plate, which contained the test compounds dispersed in 100 µL malt extract agar containing bromocresol green (50 µg/mL). The plates were incubated at 28 °C for 48 h during which time the positive control wells change colour from a blue to yellow colour. MIC end points were determined visually. The absorbance was measured using Multiskan Skyhigh Microplate Spectrophotometer (Thermofisher Scientific) at 605 nm.

Phytox is a generic bioassay platform for herbicidal discovery. In the present study, *Eragrostis tef* (teff) seed was used as indicative monocotyledon species for herbicidal discovery. Teff seeds (10 to 15) were dispensed using a LabTIE seed dispenser into the wells of a 96-well microtitre plate containing the test compounds dispersed in 200 µL of agar (1% w/v) per well. The plates were placed in a tray wrapped with semi-opaque bag, exposed to 1600 lux (inside the bag) using Power-Glo (20 W) and Sun-Glo (20 W) tubes, and incubated for 72 h at 24 °C.

Supplementary Tables

Table S1. A list of primers used in the study

Name of primers	Sequence (5′ → 3′)
pyrG_*ltbA*-F1_F	CGTTAATTAGAACTCTTCCAATCCTATCACTCGCCTTAATATGTCTGCAACACCAGCTCC
pyrG_*ltbA*-F1_R	GACTTCGTCATGTCCGTGT
pyrG_*ltbA*-F2_F	AGATCACGGTCGAGTACGAGA
pyrG_*ltbA*-F2_R	ACGTAGGTACCATCCTTGC
pyrG_ltbA-F3_F	CATTGATGAGATCGAGGACG
pyrG_*ltbA*-F3_R	AACACAGTGGAGGACATACCCGTAATTTTCTGGGCTTAATTTATTCCTGACCAGCCTGAA
pyrG_*ltbA*△-cAT F3_R	AACACAGTGGAGGACATACCCGTAATTTTCTGGGCTTAATTCCTTATTCTCTGCCTGAGT
ribO-*ltbC*-F	AGTTAATTAGAACTCTTCCAATCCTATCACCTCGCCTTAATATGGCAAGCACCGGTCAAA
ribO-*ltbC*-R	GCGCTCCACGGGGACTCGCTTCAATTTGTTCCGCTTAATCTACTTCCACGCGAGCATATC
ribO-*ltbB*-F	AGGTCTACAATCAATTCAGGCCGTATTCAGGGCCTAATCTTCAATATCTTTTGTGTCCGC
ribO-*ltbB*-R	TTTGAGATACCAAAGCATTGAGCCCAGAAACAGCAGAAGCATGGCAGTGCCTTTAGGAGC
ribO-*ltbD*-F	CTTTTCCTTATTAAACCATATATCTCCAGCAGAGCGATATGTTCTTAGGTCCTAACGGCC
ribO-*ltbD*-R	AAGCCAACTTCGATCTCAACTAAATTATCTCCTCCCGCGATCTATATGGCCCTGTCGGCC
ltbA-xw55-F	CATATGGCTAGCGATTATAAGGATGATGATGATAAGACTAGTATGTCTGCAACACCAGCT
ltbA-xw55-R	CAAATTTGTCATTTAAATTAGTGATGGTGATGGTGATGCACTTCCTGACCAGCCTGAATA
ltbB- pXP741-F	TCAACTATCAACTATTAACTATATCGTAATACACAACTAGATGGCAGTGCCTTTAGGA
ltbB- pXP741-R	TGTAAGCGTGACATAACTAATTACATGACTCGACTAATCTTCAATATCTTTTGTGTCCGC
cAT standalone F	GGCCTGGTGCCGCGCGGCAGCCATCTACCTAGGCACCCGC
cAT standalone R	ATTTGCTGTCCACCAGTCATGCTAGCTTATTCCTGACCAGCCTGAATAA
ACP-cAT F	GGCCTGGTGCCGCGCGGCAGCCATGAAACGATGGAGGAGGCC

Table S2. DNA plasmids constructed in the study

Plasmid Name	Plasmid Backbone	Description of the plasmid
pYFAC-CL50	pYFAC-*pyrG*	*A. nidulans* expression vector containing *ltbA* gene under the *alcA* promoter
pYFAC-CL51	pYFAC-*ribo*	*A. nidulans* expression vector containing *ltbB* gene under *alcS* promoter, *ltbC* gene under *alcA* promoter and *ltbD* gene under *alcM* promoter
pYFAC- CL52	pYFAC-*ribo*	*A. nidulans* expression vector containing *ltbB* gene under *alcS* promoter and *ltbC* gene under *alcA* promoter
pYFAC- CL53	pYFAC-*ribo*	*A. nidulans* expression vector containing *ltbC* gene under *alcA* promoter
pYFAC- CL54	pYFAC-*ribo*	*A. nidulans* expression vector containing *ltbB* gene under *alcS* promoter
pXW55-*ltbA*	pXW55	2µ *S. cerevisiae* expression vector containing gene *ltbA* under the ADH2 promoter
pXP741-*ltbB*	pXP741	2µ *S. cerevisiae* expression vector containing gene *ltbB* under the ADH2 promoter
pET-28a-cAT	pET-28a	pET plasmid with *N*-6xHis containing the intron-free *ltbA*-cAT domain under the T7 promoter
pET-28a-ACP-cAT	pET-28a	pET plasmid with *N*-6xHis containing the intron-free *ltbA*-ACP-cAT domain under the T7 promoter

Table S3. Expression strains used in the study

Strain name	Organism	Description of the strain
A. nidulans_ltbABCD	*A. nidulans* LO8030	*A. nidulans* LO8030 expressing pYFAC-CL50 and pYFAC-CL51
A. nidulans_ltbA	*A. nidulans* LO8030	*A. nidulans* LO8030 expressing pYFAC-CL50
A. nidulans_ltbABC	*A. nidulans* LO8030	*A. nidulans* LO8030 expressing pYFAC-CL50 and pYFAC- CL52
A. nidulans_ltbAC	*A. nidulans* LO8030	*A. nidulans* LO8030 expressing pYFAC-CL50 and pYFAC- CL53
A. nidulans_ltbAB	*A. nidulans* LO8030	*A. nidulans* LO8030 expressing pYFAC-CL50 and pYFAC- CL54
S. cerevisiae ltbA	*S. cerevisiae* BJ5464-npgA	*S. cerevisiae* BJ5464-npgA expressing pXW55-*ltbA*
S. cerevisiae ltbAB	*S. cerevisiae* JHY702	*S. cerevisiae* JHY702 expressing pXW55-*ltbA* and pXP741-*ltbB*
E. coli-cAT domain	*E. coli Rosetta*(DE3)	*E. coli* Rosetta(DE3) overexpressing pET-28a-cAT
E. coli-ACP-cAT	*E. coli Rosetta*(DE3)	*E. coli* Rosetta(DE3) overexpressing pET-28a-ACP-cAT

Table S4. Annotation of genes in the *ltb* cluster

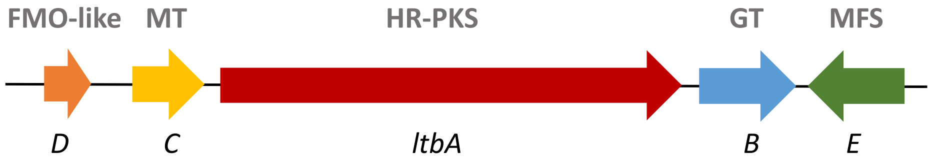

Gene	Locus tag	Predicted function	Closest BLAST Homologue	Identity/coverage (%)
ltbA	ALUT_005489	polyketide synthase	*Penicillium* sp. CMV-2018d (KAJ5406191.1)	84.88/99
ltbB	ALUT_005490	glycosyltransferase	*Penicillium* sp. CMV-2018d (KAJ5406192.1)	85.33/100
ltbC	ALUT_005488	methyltransferase	*Penicillium odoratum* (XP_056996551.1)	88.42/100
ltbD	ALUT_005487	flavin-dependent monooxygenase	*Aspergillus brasiliensis* CBS 101740 (OJJ66157.1)	84.52/100
ltbE	ALUT_005491	MFS transporter	*Aspergillus fumigatiaffinis* (KAF4221576.1)	93.59/99

Table S5. ^{1}H (600 MHz) and ^{13}C (150 MHz) NMR data for luteodienoside A (**1**) and luteodienoside B (**3**) in DMSO-d_6

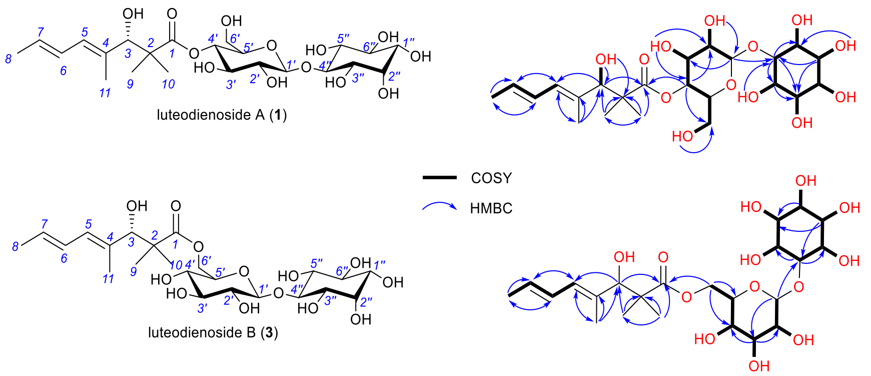

Pos.	Luteodienoside A (1)		Luteodienoside B (3)	
	δ_C, type	δ_H, mult. (J in Hz)	δ_C, type	δ_H, mult. (J in Hz)
1	175.7, C		176.1, C	
2	48.0, C		48.1, C	
3	79.6, CH	4.21, d (4.5)	79.1, CH	4.21, br s
4	134.6, C		135.4, C	
5	127.5, CH	5.93, dq (10.9, 1.7)	126.9, CH	5.91, dq (10.9, 1.7)
6	127.4, CH	6.26, ddd (14.5, 10.9, 1.7)	127.7, CH	6.24, ddd (14.3, 10.9, 1.7)
7	128.8, CH	5.67, dq (14.5, 6.8)	128.6, CH	5.65, dq (14.3, 6.7)
8	18.2, CH_3	1.75, dd (6.8, 1.7)	18.3, CH_3	1.74, dd (6.7, 1.7)
9	23.0, CH_3	0.99, s	21.1, CH_3	0.99, s
10	19.4, CH_3	1.04, s	21.3, CH_3	1.07, s
11	14.3, CH_3	1.68, s	14.1, CH_3	1.65, s
3-OH		5.05, d (4.5)		
**D-Glucose**				
1′	103.9, CH	4.41, d (7.9)	103.6, CH	4.36, d (7.9)
2′	73.7, CH	3.16[a]	73.5, CH	3.07[d]
3′	74.4, CH	3.46[b]	75.9, CH	3.20, dd (8.9, 8.9)
4′	71.4, CH	4.52, t (9.5)	70.1, CH	3.09[d]
5′	73.6, CH	3.46[b]	74.1, CH	3.43[e]
6′	60.7, CH_2	3.28, ddd (11.1, 6.9, 3.2)	63.5, CH_2	3.86, dd (11.8, 7.2)
		3.47[b]		4.42, dd (11.8, 2.1)
2′-OH		5.44, d (3.7)		
3′-OH		5.15, d (5.6)		
6′-OH		4.61, d (5.5)		
***myo*-Inositol**				
1″	71.4, CH	3.16[a]	71.6, CH	3.16, dd (9.6, 2.6)
2″	71.9, CH	3.76, dd (2.9, 2.9)	71.9, CH	3.75, dd (2.6, 2.6)
3″	70.6, CH	3.39[c]	70.5, CH	3.39[e]
4″	85.3, CH	3.54, dd (9.3, 9.3)	84.7, CH	3.54, dd (9.4, 9.4)
5″	73.5, CH	3.09, ddd (8.9, 8.9, 2.9)	73.1, CH	3.08[d]
6″	72.6, CH	3.41[c]	72.7, CH	3.41[e]
1″-OH		4.45, d (5.6)		
2″-OH		4.68, d (3.7)		
3″-OH		4.59, d (4.0)		
5″-OH		4.35, d (2.9)		
6″-OH		4.65, d (4.6)		

a-e: overlapping signals.

Table S6. ^{1}H (600 MHz) and ^{13}C (150 MHz) NMR data for luteodiene A (**2**) in CD_3OD

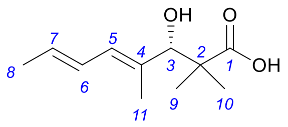

luteodiene A (**2**)

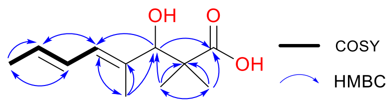

Pos.	δ_C, type	δ_H, mult. (J in Hz)
1	181.2, C	
2	48.3, C	
3	82.6, CH	4.22, s
4	135.3, C	
5	129.6, CH	5.98, dq (10.7, 1.2)
6	128.5, CH	6.29, ddd (14.0, 10.7, 1.7)
7	130.3, CH	5.68, dq (14.0, 6.8)
8	18.5, CH_3	1.77, dd (6.8, 1.7)
9	23.4, CH_3	1.12, s
10	21.7, CH_3	1.13, s
11	14.0, CH_3	1.73, d (1.2)

Table S7. ^{1}H (600 MHz), ^{13}C (150 MHz) and 2D NMR data for glucinol in D_2O.

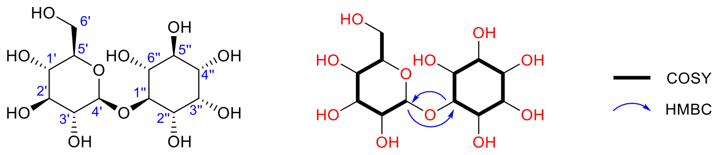

Pos.	δ_C, type	δ_H, mult. (J in Hz)	COSY	HMBC
1′	69.5, CH	3.37[c]	2′	6′
2′	75.6, CH	3.48[b]	1′, 3′	1′, 3′
3′	73.5, CH	3.31, m	2′, 4′	2′, 4′
4′	102.9, CH	4.66, d (7.9)	3′	1″
5′	75.9, CH	3.43, m	6′a, 6′b	1′
6′a	60.7, CH_2	3.87, dt (4.1, 2.9)	5′, 6′b	1′
6′b		3.69[a]	5’, 6’a	5’
1″	82.1, CH	3.77, t (9.6)	2″, 6″	4′, 2″, 6″
2″	70.8, CH	3.69[a]	1″, 3″	1″
3″	72.1, CH	4.02, m	2″, 4″	1″, 2″, 5″
4″	71.0, CH	3.49[b]	3″	5″
5″	72.3, CH	3.61, t (9.6)	6″	4″, 6″
6″	72.6, CH	3.35[c]	1″, 5″	1″, 5″

a-c: overlapping signals. Note: glycerol impurity present in the NMR sample.

Table S8. Crystal data and structure refinement for luteodienoside A (**1**)

Identification code	amp17pt2_s1582_2
Empirical formula	$C_{23}H_{46}O_{17}$
Formula weight	594.60
Temperature/K	100(2)
Crystal system	orthorhombic
Space group	$P2_12_12_1$
a/Å	6.60006(18)
b/Å	7.7434(3)
c/Å	55.3300(13)
α/°	90
β/°	90
γ/°	90
Volume/Å^3	2827.75(16)
Z	4
ρ_{calc}g/cm^3	1.397
μ/mm^{-1}	1.028
F(000)	1280.0
Crystal size/mm^3	0.244 × 0.043 × 0.028
Radiation	Cu Kα (λ = 1.54184)
2Θ range for data collection/°	9.59 to 155.984
Index ranges	$-7 \le h \le 8$, $-9 \le k \le 9$, $-69 \le l \le 69$
Reflections collected	53625
Independent reflections	5946 [R_{int} = 0.0667, R_{sigma} = 0.0303]
Data/restraints/parameters	5946/70/416
Goodness-of-fit on F^2	1.146
Final R indexes [I>=2σ (I)]	R_1 = 0.0528, wR_2 = 0.0992
Final R indexes [all data]	R_1 = 0.0562, wR_2 = 0.1013
Largest diff. peak/hole / e Å^{-3}	0.27/−0.30
Flack parameter	−0.04(7)

Table S9. Fractional atomic coordinates (×10[4]) and equivalent isotropic displacement parameters (Å[2]×10[3]) for luteodienoside A (**1**). U_{eq} is defined as 1/3 of the trace of the orthogonalised U_{IJ} tensor.

**Atom**	***x***	***y***	***z***	**U(eq)**
O1	2640(4)	8471(3)	6489.4(4)	12.4(5)
C1	630(5)	8488(5)	6545.5(6)	13.3(7)
O2	−684(4)	8145(4)	6404.3(5)	23.0(6)
C2	303(6)	8868(5)	6814.8(6)	15.8(7)
C3	1716(6)	10360(5)	6893.1(6)	14.1(7)
O3	1206(4)	11870(4)	6755.4(5)	17.5(5)
C4	1674(6)	10765(5)	7162.3(6)	18.0(8)
C5	3068(6)	10030(5)	7304.4(7)	18.0(8)
C6	3234(6)	10158(5)	7566.3(7)	21.0(8)
C7	4577(7)	9234(6)	7695.4(7)	25.1(9)
C8	4682(7)	9172(6)	7963.8(7)	30.2(10)
C9	−1950(6)	9253(6)	6856.1(7)	21.9(9)
C10	895(7)	7197(5)	6949.2(7)	22.8(9)
C11	78(7)	11975(6)	7255.6(7)	27.7(10)
C1″	11090(5)	3512(5)	5291.8(6)	11.6(7)
O1″	12728(4)	2428(4)	5223.0(5)	17.1(6)
O2″	12563(4)	3921(3)	5676.6(4)	14.2(5)
C2″	11778(5)	4857(5)	5474.4(6)	11.5(7)
O3″	10692(4)	7252(3)	5713.8(5)	15.4(5)
C3″	10001(5)	5993(4)	5544.7(6)	9.9(7)
C4″	8294(5)	4903(4)	5652.0(6)	9.4(7)
O4″	6226(4)	2333(3)	5593.1(5)	13.4(5)
C5″	7625(5)	3474(4)	5479.3(6)	9.5(7)
O5″	8826(4)	1179(3)	5216.1(5)	13.7(5)
C6″	9419(5)	2386(4)	5398.2(6)	10.1(7)
O1′	7185(4)	9498(3)	5824.7(4)	13.8(5)
C1′	6308(5)	6654(5)	5928.8(6)	10.8(7)
O2′	3669(4)	10770(3)	6089.1(5)	16.9(5)
C2′	5517(5)	8494(4)	5905.6(6)	11.3(7)
C3′	4660(5)	9192(5)	6143.7(6)	11.5(7)
O3′	4026(4)	3190(4)	6441.4(5)	16.6(5)
O4′	4849(4)	5555(3)	6040.1(4)	10.9(5)
C4′	3213(5)	7872(5)	6250.9(6)	13.1(7)
C5′	4293(5)	6136(5)	6277.8(6)	12.3(7)
O5′	6577(4)	6023(3)	5692.8(4)	10.3(5)
C6′	2966(6)	4754(5)	6385.7(6)	15.4(8)
O1W	8027(5)	3427(5)	6490.2(6)	32.9(7)
O2W	9570(5)	1839(4)	6094.4(6)	29.4(7)
O3W	6060(4)	−1524(4)	5269.7(5)	18.9(6)
O4W	7047(5)	−3688(4)	4907.6(5)	25.8(7)

Table S10. Anisotropic displacement parameters (Å^2×10^3) for luteodienoside A (**1**). The anisotropic displacement factor exponent takes the form: $-2\pi^2[h^2a^{*2}U_{11}+2hka^*b^*U_{12}+\ldots]$.

**Atom**	**U_{11}**	**U_{22}**	**U_{33}**	**U_{23}**	**U_{13}**	**U_{12}**
O1	10.6(11)	16.0(13)	10.8(11)	−3.2(9)	2.4(9)	−1.4(10)
C1	13.6(17)	7.8(17)	18.6(17)	0.3(13)	2.7(13)	−0.5(14)
O2	10.9(13)	37.3(18)	20.9(13)	−6.8(12)	1.3(10)	−1.4(12)
C2	15.4(17)	16.0(19)	16.1(16)	−0.3(14)	6.7(14)	−1.1(15)
C3	15.0(17)	12.5(18)	14.7(16)	0.7(14)	1.4(14)	3.8(15)
O3	19.4(14)	15.7(14)	17.4(12)	4.0(10)	2.0(10)	−0.1(12)
C4	19.5(18)	19(2)	15.1(17)	−0.5(15)	2.7(14)	−0.7(17)
C5	18.6(19)	15.6(19)	19.7(18)	0.2(15)	2.1(15)	0.5(16)
C6	20.2(19)	22(2)	21.0(19)	1.1(16)	0.9(15)	0.1(17)
C7	25(2)	26(2)	24(2)	1.2(17)	−1.2(17)	0.6(19)
C8	30(2)	34(3)	27(2)	3.1(19)	−6.2(18)	−2(2)
C9	16.0(19)	23(2)	26(2)	−4.9(16)	9.5(15)	−3.5(17)
C10	33(2)	17(2)	18.3(18)	1.2(15)	6.5(16)	−3.2(18)
C11	27(2)	36(3)	19.8(19)	−9.9(18)	0.2(16)	11(2)
C1″	12.3(16)	9.1(17)	13.3(15)	−0.9(13)	2.4(13)	0.7(14)
O1″	14.2(13)	18.3(14)	18.8(13)	−1.6(11)	6.6(10)	4.6(11)
O2″	12.9(12)	10.7(13)	19.2(12)	−1.7(10)	−6.0(10)	1.7(10)
C2″	10.0(15)	6.2(16)	18.2(17)	0.8(13)	0.3(13)	−0.5(13)
O3″	11.0(13)	10.3(13)	24.9(13)	−6.8(10)	−0.7(10)	−0.4(10)
C3″	10.1(16)	5.0(16)	14.6(15)	−2.7(12)	−0.1(12)	−0.6(13)
C4″	6.3(15)	9.2(17)	12.8(16)	0.1(12)	0.4(12)	2.3(13)
O4″	6.0(11)	11.7(13)	22.5(12)	2.1(10)	3.1(10)	−0.5(10)
C5″	10.9(16)	6.1(17)	11.4(15)	0.7(12)	0.1(13)	−0.5(13)
O5″	14.5(12)	8.5(12)	18.1(12)	−6.4(10)	1.0(10)	−2.3(10)
C6″	10.3(16)	7.2(16)	12.7(15)	−4.2(12)	−1.3(12)	−2.9(13)
O1′	12.9(13)	9.6(13)	19.1(12)	2.5(10)	3.4(10)	−1.1(10)
C1′	10.8(16)	12.2(17)	9.3(14)	−0.8(13)	2.0(12)	−1.3(14)
O2′	21.4(14)	8.4(13)	20.8(13)	−1.6(10)	2.3(11)	6.1(11)
C2′	12.3(16)	9.1(17)	12.4(15)	−1.1(13)	2.2(13)	1.9(14)
C3′	10.9(15)	9.8(17)	13.7(15)	−1.3(13)	0.5(13)	−1.5(14)
O3′	15.7(12)	12.7(13)	21.5(13)	2.4(10)	1.3(11)	−1.3(12)
O4′	12.1(12)	9.6(12)	10.9(11)	−1.8(9)	3.2(9)	−2.7(10)
C4′	12.6(16)	17.1(19)	9.6(15)	−1.1(13)	2.0(13)	−0.1(15)
C5′	13.6(17)	15.3(18)	8.1(14)	−0.6(13)	1.1(12)	−3.6(15)
O5′	9.8(11)	11.3(12)	9.8(11)	−2.7(9)	1.4(9)	1.2(10)
C6′	16.6(19)	13.4(19)	16.1(17)	1.0(14)	4.8(14)	−1.0(15)
O1W	23.3(16)	37(2)	38.5(18)	0.4(15)	0.1(14)	0.3(15)
O2W	22.9(15)	28.1(17)	37.1(17)	−1.7(14)	−5.1(13)	−5.6(14)
O3W	22.9(14)	16.4(14)	17.4(12)	−1.2(10)	−0.2(11)	−1.0(12)
O4W	28.1(16)	17.1(16)	32.1(16)	−10.0(12)	5.4(13)	−5.5(13)

Table S11. Biological testing of compounds **1**–**3**.

Compound	Minimum inhibitory concentration (μg/mL)								
	**Bs 24 h**	**Bs 48 h**	**Sa 24 h**	**Sa 48 h**	**Ca 24 h**	**Ca 48 h**	**Sc 24 h**	**Sc 48 h**	**Teff 72 h**
Luteodienoside A (**1**)	>100	>100	>100	>100	>200	>200	>200	>200	>100
Luteodiene A (**2**)	>100	>100	>100	>100	>200	>200	>200	>200	>100
Luteodienoside B (**3**)	>100	>100	>100	>100	>200	>200	>200	>200	>100

Bacillus subtilis (Bs), *Staphylococcus aureus* (Sa), *Candida albicans* (Ca), *Saccharomyces cerevisiae* (Sc), and *Eragrostis tef* (Teff).

Supplementary Figures

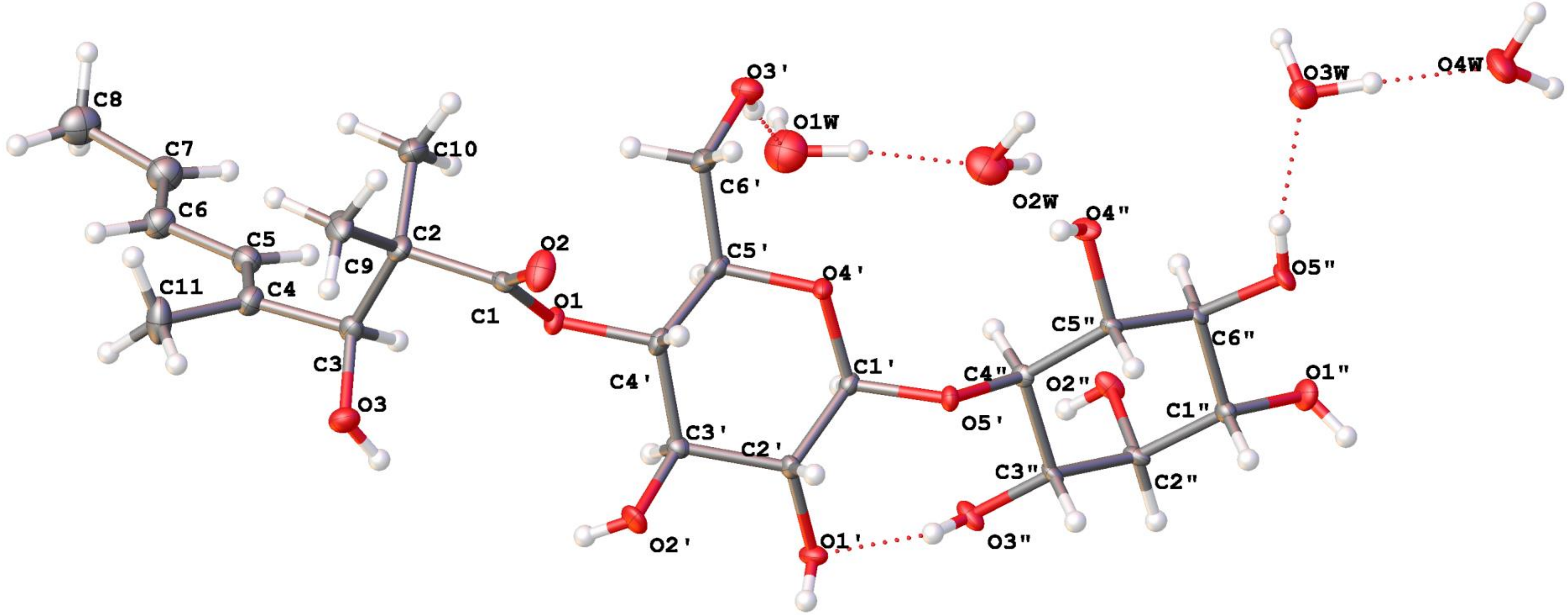

Figure S1. Olex2 depiction of luteodienoside A (**1**) with 50% displacement ellipsoids.

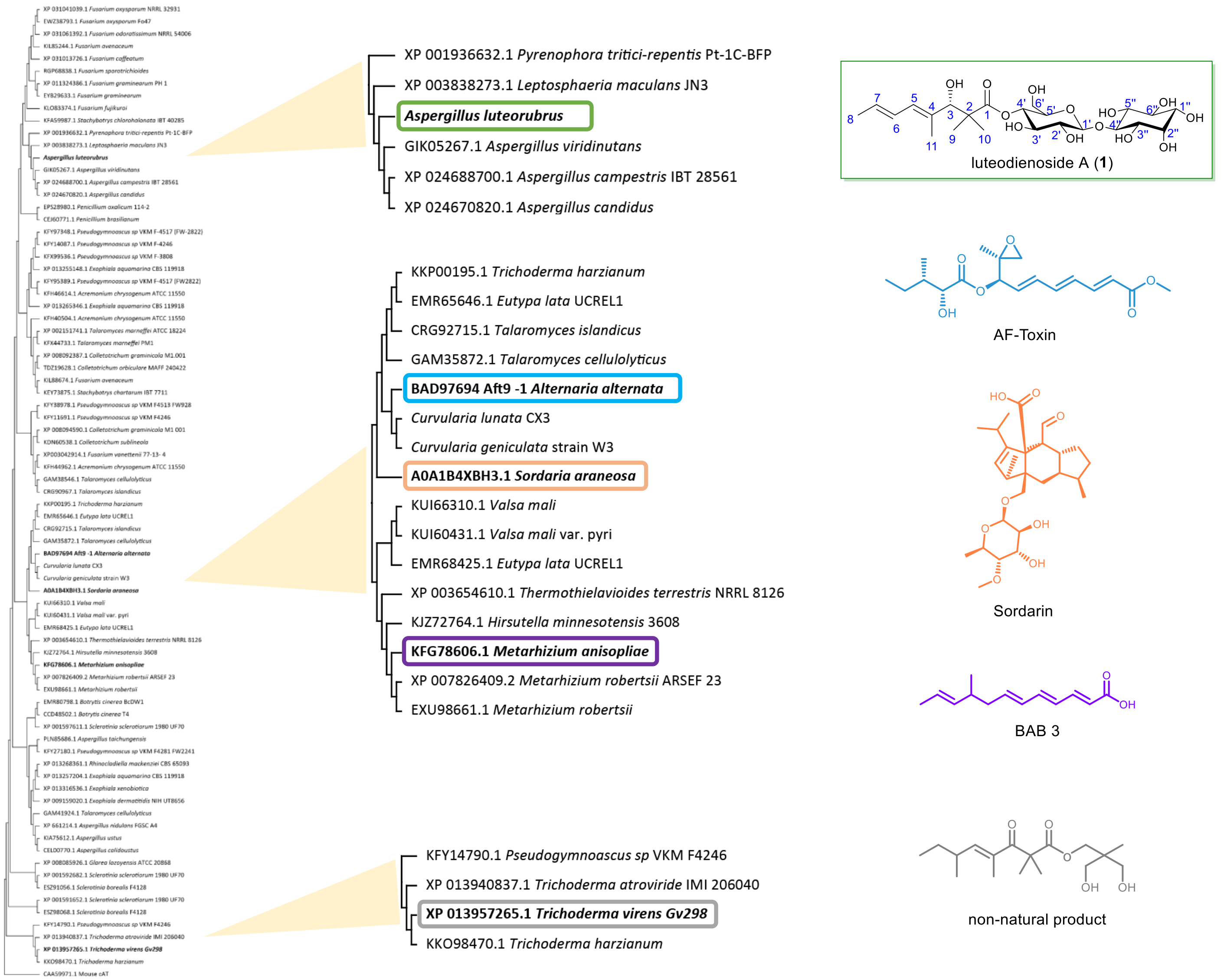

Figure S2. Phylogenetic tree for the cAT domains and the products of the corresponding HR-PKSs. The tree was constructed using Neighbour Joining method and 1000 bootstrap in MEGA-11 . The cAT domain of mouse was used as an outgroup.

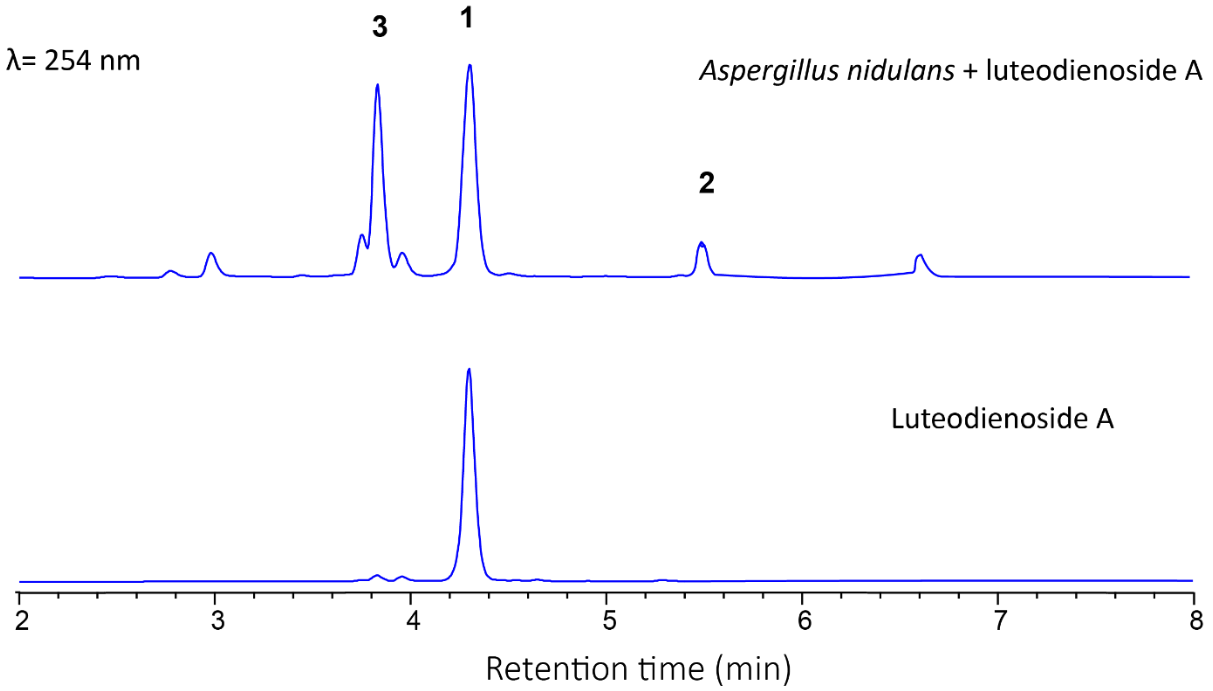

Figure S3. LC-DAD-MS analysis of the *n*-butanol extract of *A. nidulans* LO8030 fed with 10 mg/L of luteodienoside A (**1**).

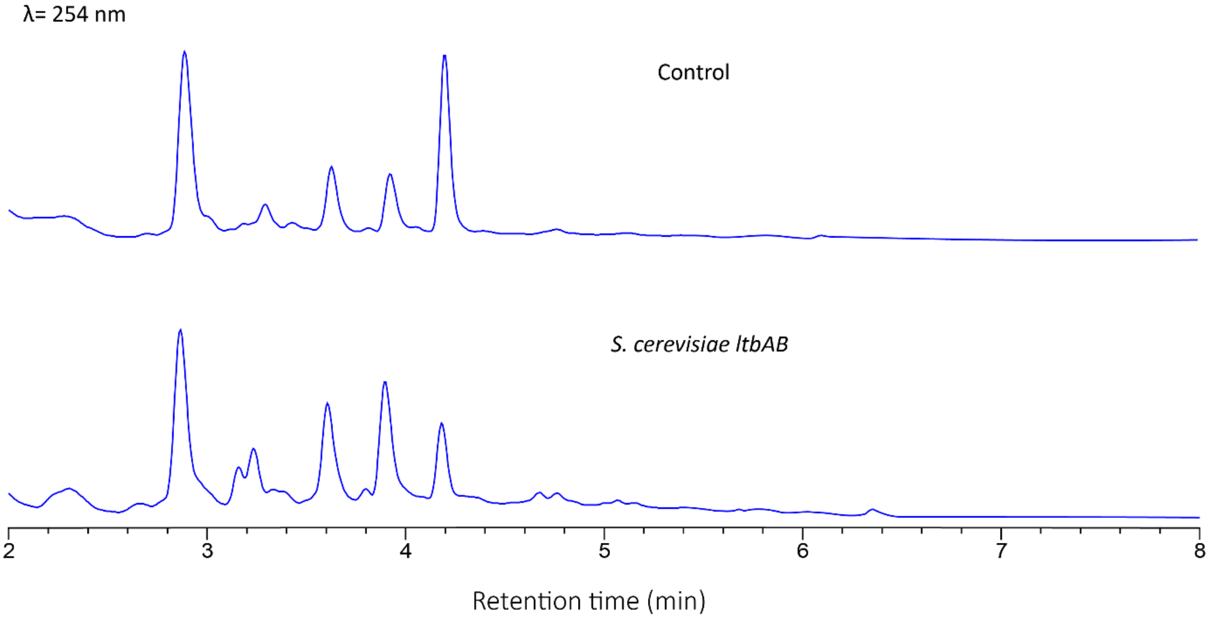

Figure S4. LC-DAD-MS analysis of the *n*-butanol extract of *S. cerevisiae* BJ5464-NpgA transformed with *ltbAB* genes.

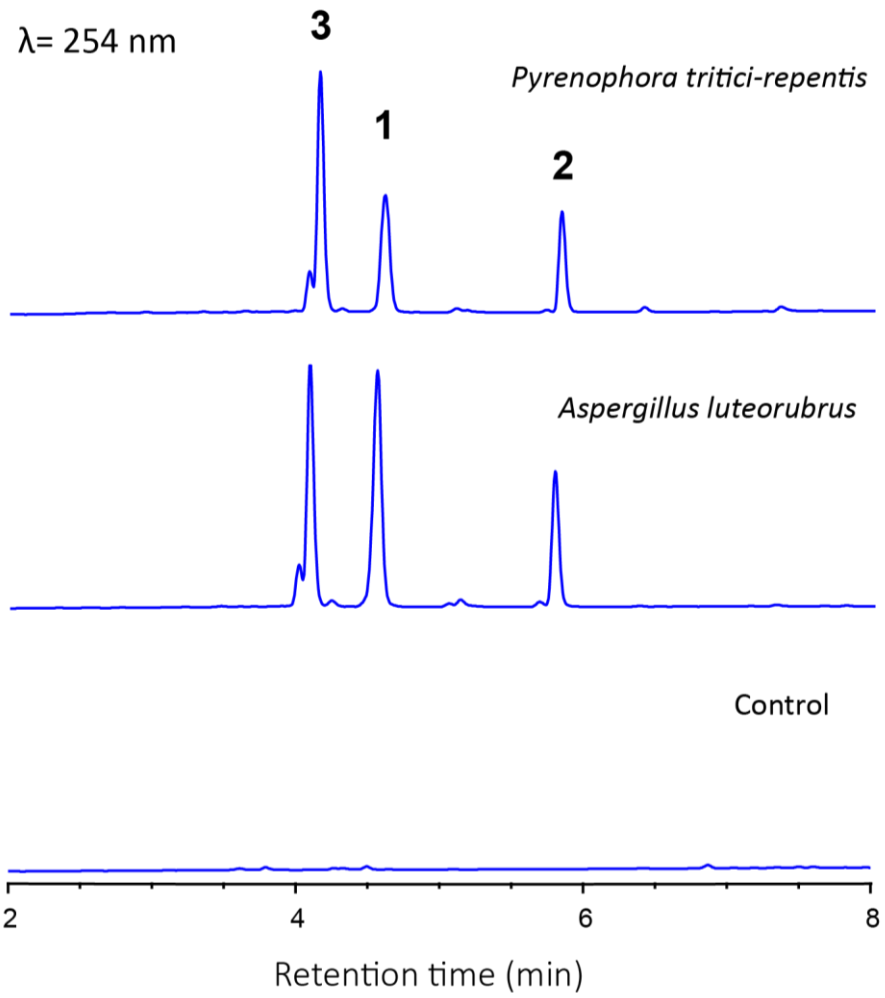

Figure S5. LC-DAD-MS analysis of the *n*-butanol extract of *A. nidulans* expressing ltb cluster from *A. luteorubrus* and *Pyrenophora tritici-repentis*.

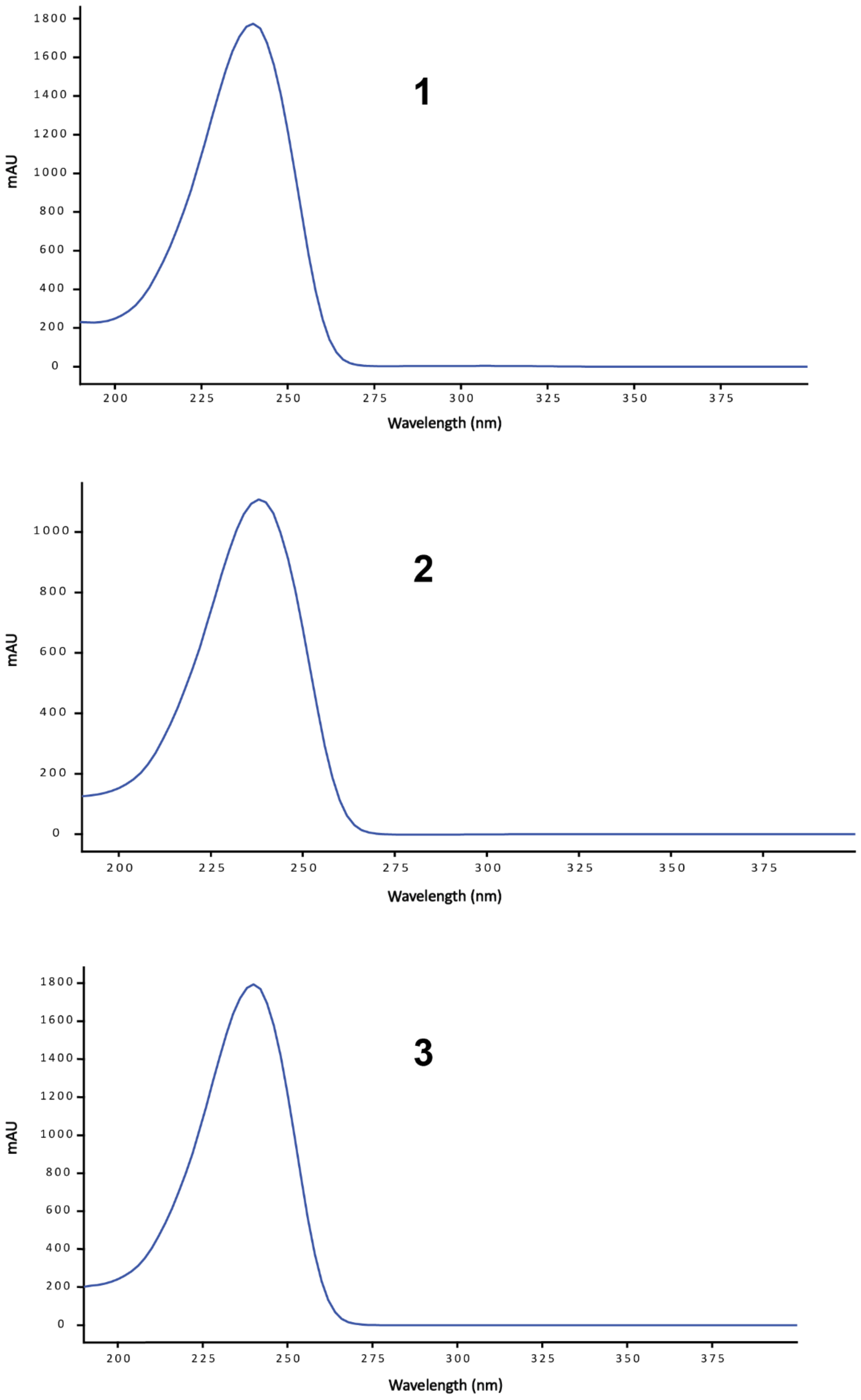

Figure S6. UV-vis spectra of **1**–**3** obtained from LC−DAD analysis (5–95% MeCN/H_2O + 0.1% formic acid)

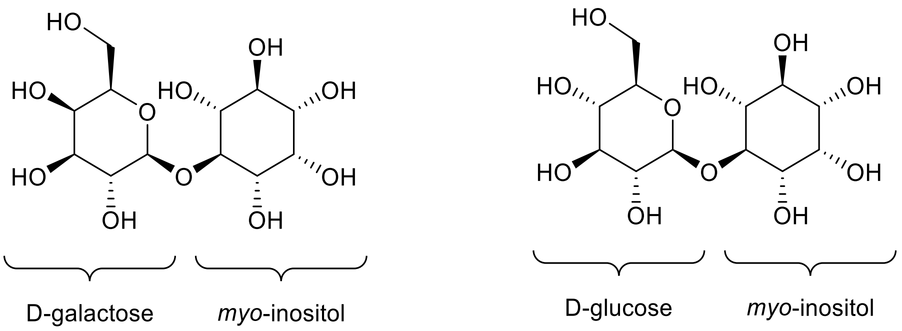

Galactinol Glucinol

Figure S7. Structures of the sugar substrates used for *in vitro* experiments

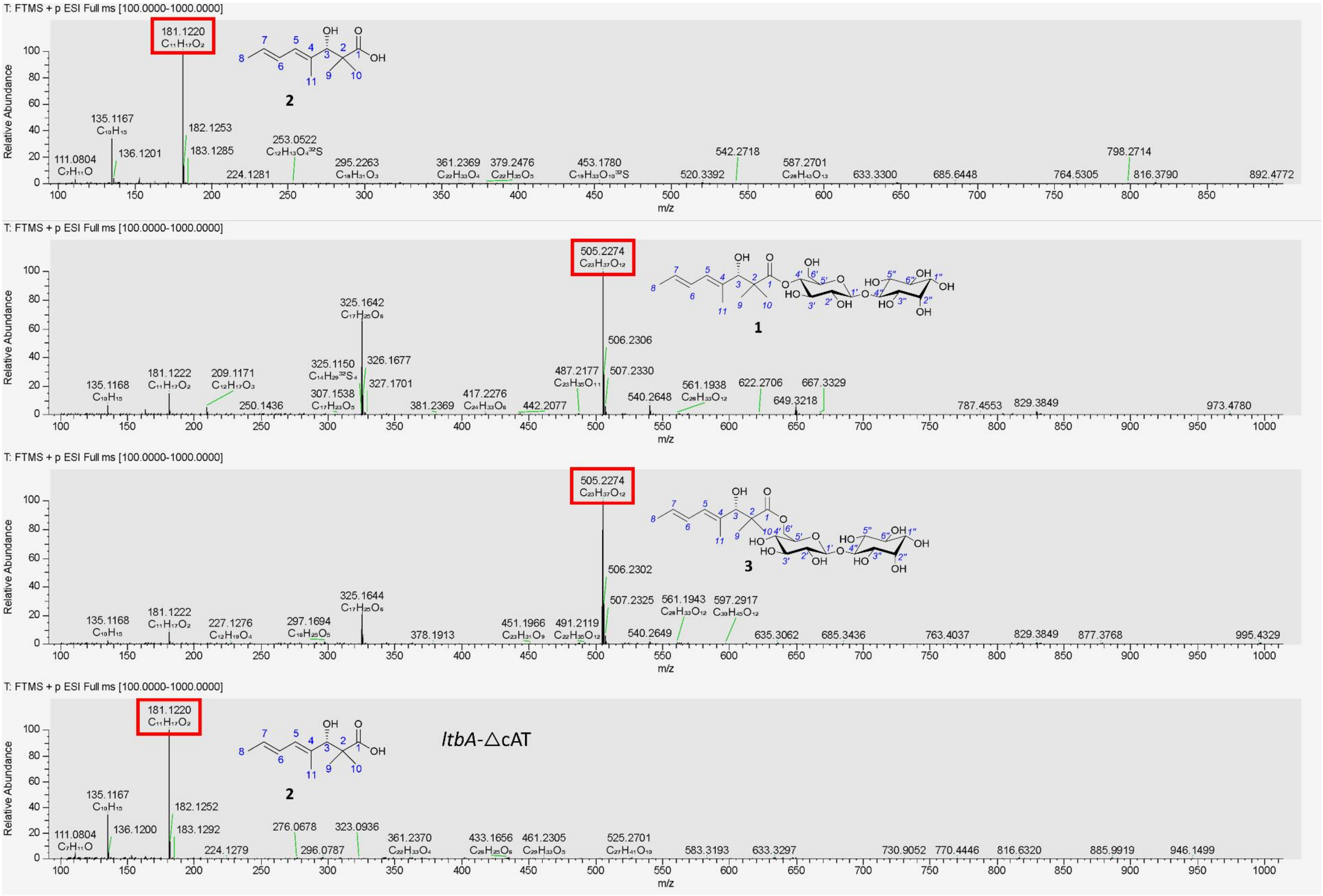

Figure S8. High resolution mass spectrometry analysis (LC−OrbiTrap) of **1**–**3** (+p ESI) of the molecules purified from *A. nidulans*.

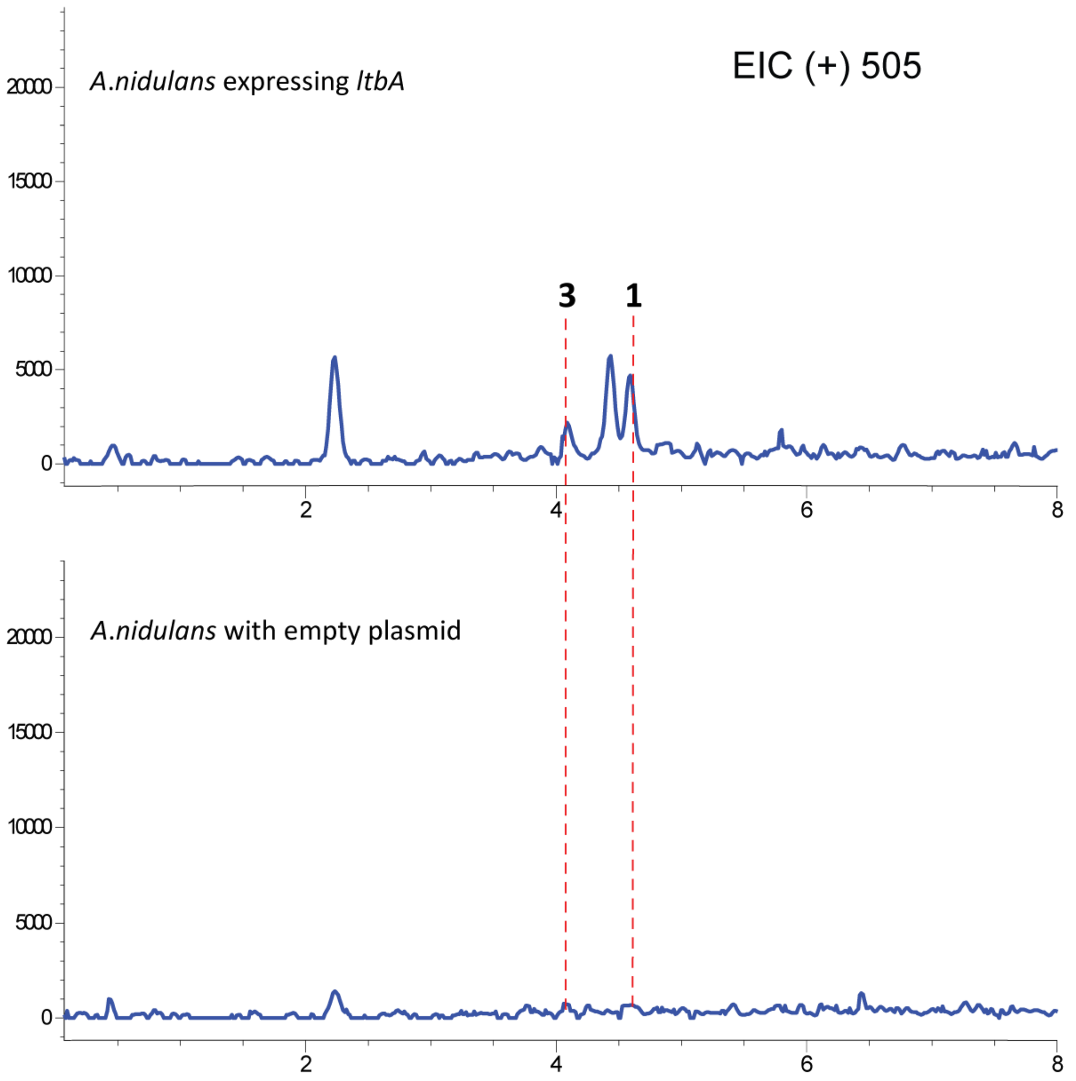

Figure S9. LCMS EIC(+) *m/z* 505 analysis of *A. nidulans* expressing *ltbA* showing the presence of traces for luteodienoside A (**1**) and luteodienoside B (**3**).

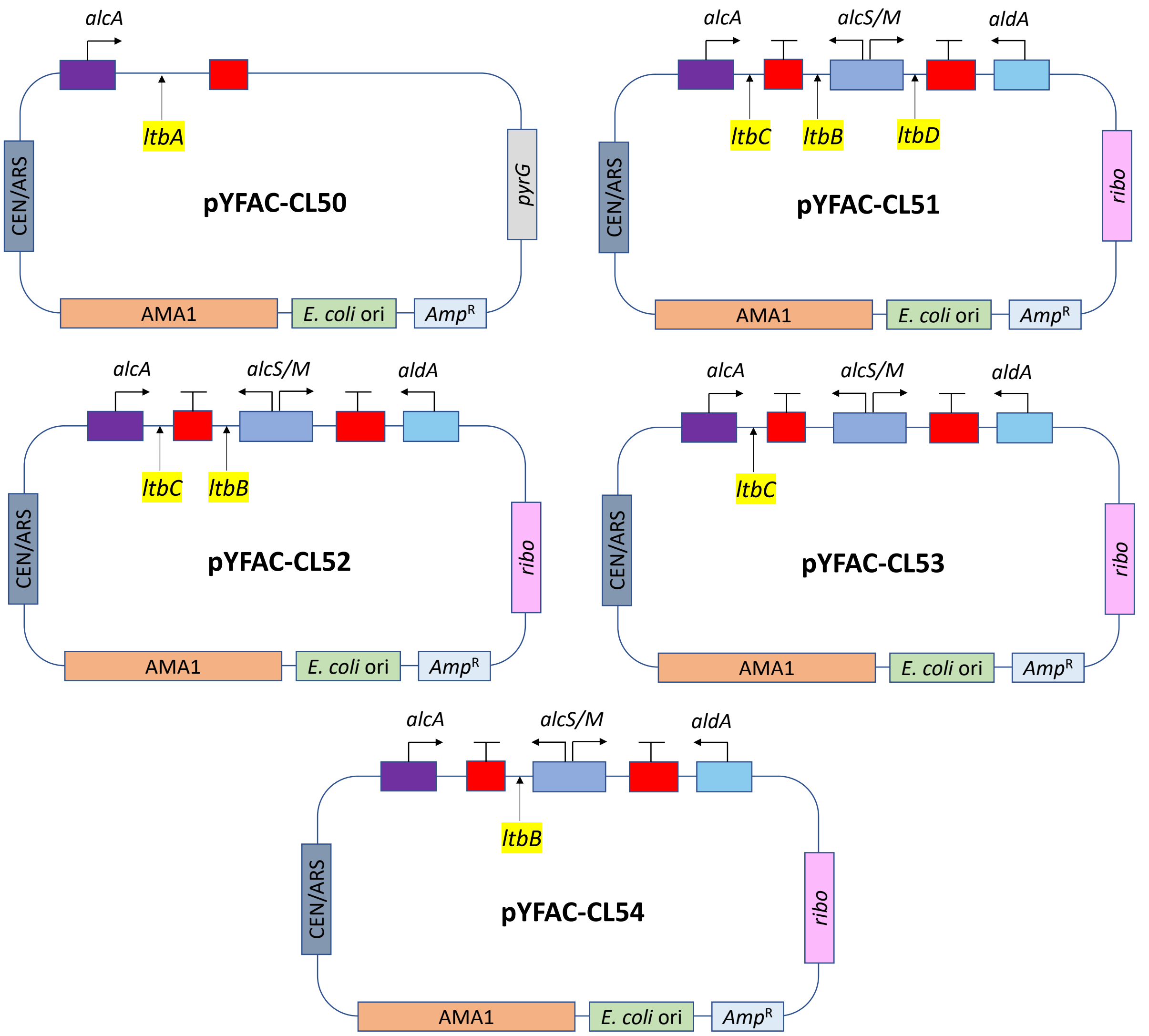

Figure S10. Schematic diagrams of plasmids constructed for the induced overexpression of *ltb* genes in the *A. nidulans* heterologous host

>LtbA cAT domain

```
LPRHPLVDLDEAIHDLLNSIGHFAHTQEEYAELRRKAHALTAPGSLGRRLYSQLRAKADDPSVESWIA
GPLLKALHLKRRYPLVPFSSFLGTHFDSAVPHSQAQRAAALTRALCEFKHDLDNRKLKPDFLGERPNC
GHSLTWLFNALREPNVGCDKMVRYPGVEHVAVLRRGHLFRVPLREGNDIVSYQKLKATYQAILNLDLE
EKLWTGILTTDNRDSWATNRQTLLALDERNTAYIKTLEESVVVMCLDDNSPVTREERVRFGYLGDSFN
RWHDKTIQLVVTANGRSGTIFEHSMIDFMTTSQISQRLQAAIDTLDPENDNHGQDVEAVVDPASLKEI
ALVATTTEIEARMIMLRDKYAATTGRKIYTPHLIPSFGKAVLLAHAVPIKATVDLTIQLASRLYFGYL
PASWETVSTAHFHLGRPEIVQVVLKSVVDFCDAALDSSVPRAEARVQLLRAAREMNAQIVKGGEGRNY
FRLMDVLEVMSHEAVDEGAADAVPELFADPVWQRSYPRLIMQTMIERKLAQDPGYTMEDPENVWMNYT
VNEDSLEVCYVSPRTGAERFKAALDRATDII
```

Figure S11. Protein sequence of the cAT.

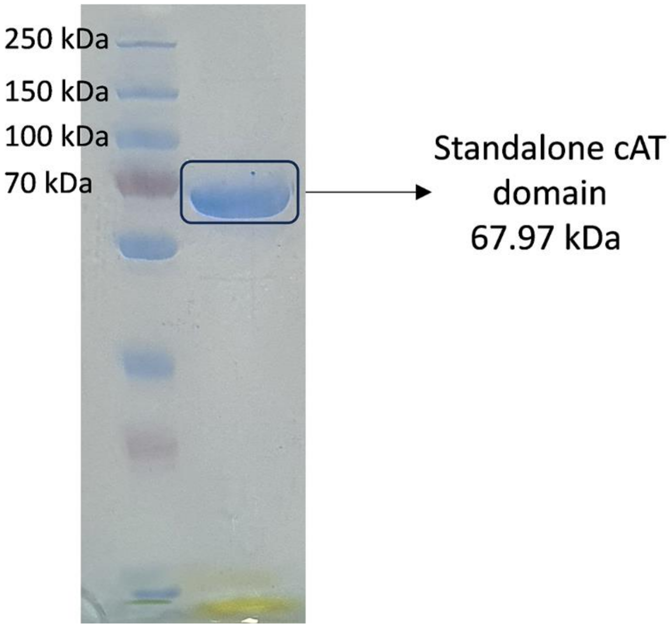

Figure S12. SDS−PAGE (10%) of the semi-purified standalone cAT domain.

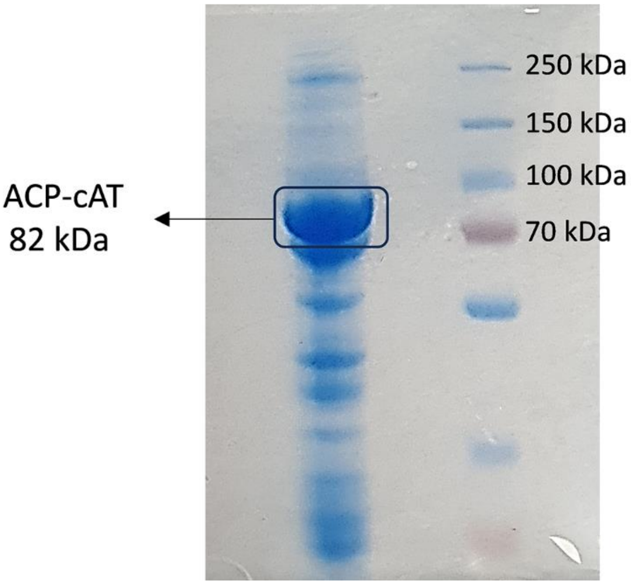

Figure S13. SDS−PAGE (10%) of the semi-purified ACP−cAT.

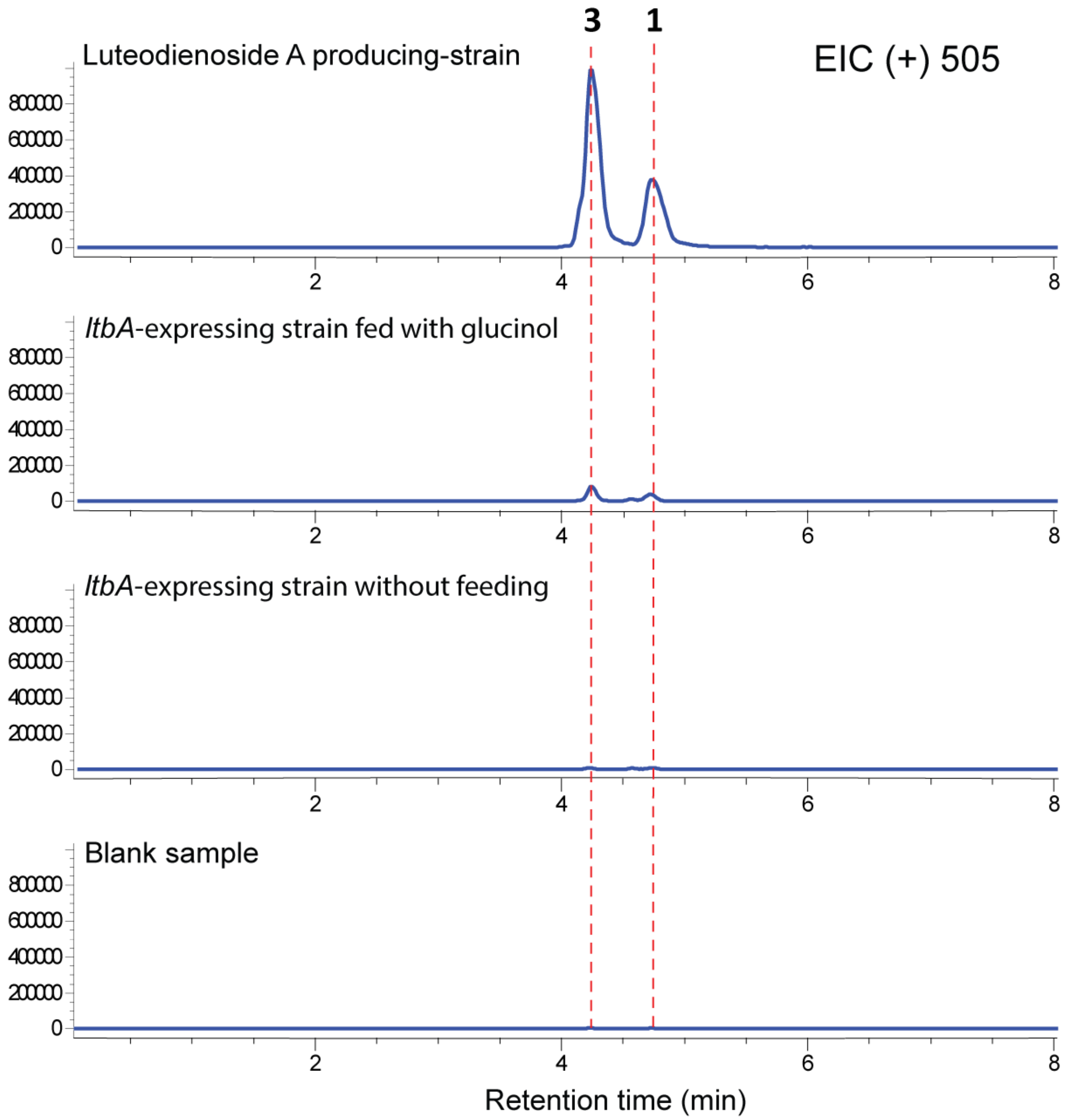

Figure S14. LCMS EIC(+) *m/z* 505 analysis of *A. nidulans* expressing *ltbA* fed with glucinol showing the presence of luteodienoside A (**1**) and luteodienoside B (**3**).

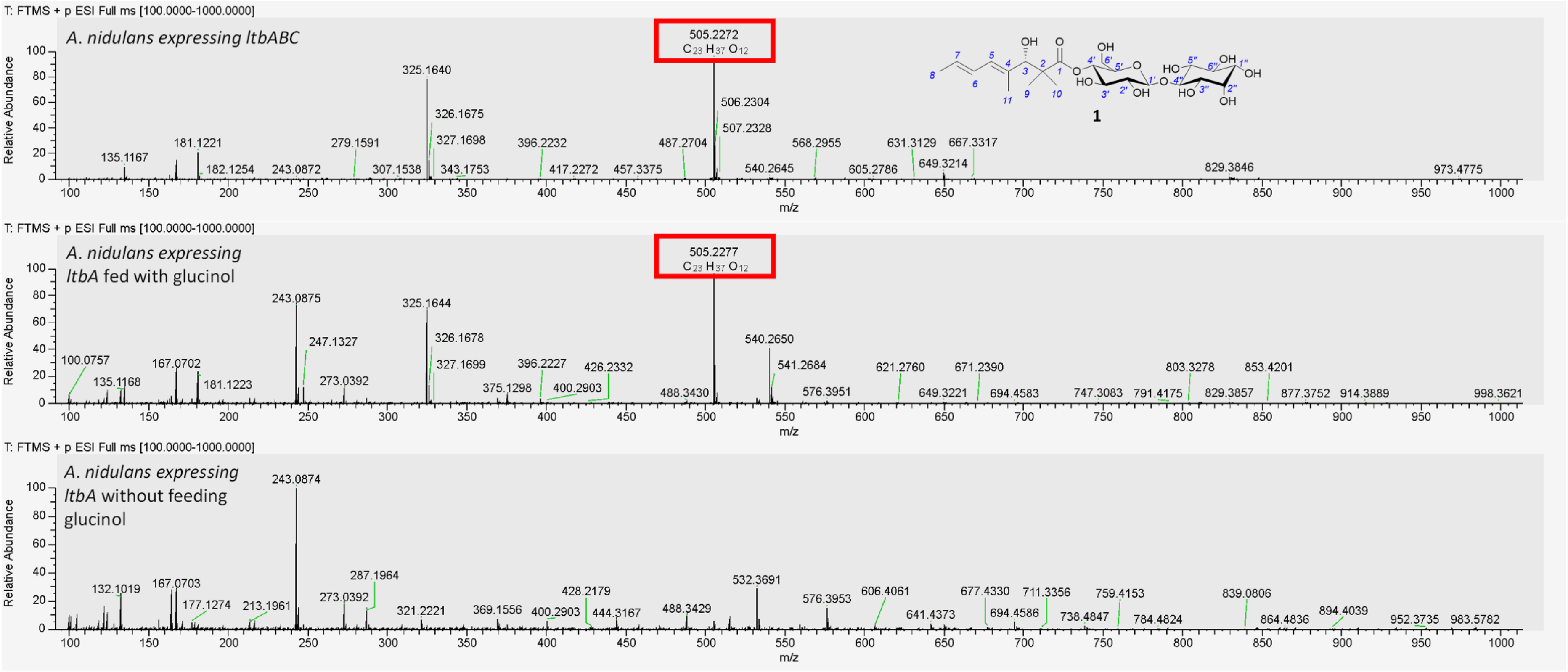

Figure S15. High resolution mass spectrometry analysis (LC−OrbiTrap) of *A. nidulans* strain expressing *ltbA* fed with glucinol showing the mass ($^{+}$p ESI) and the calculated molecular formulae of **1**.

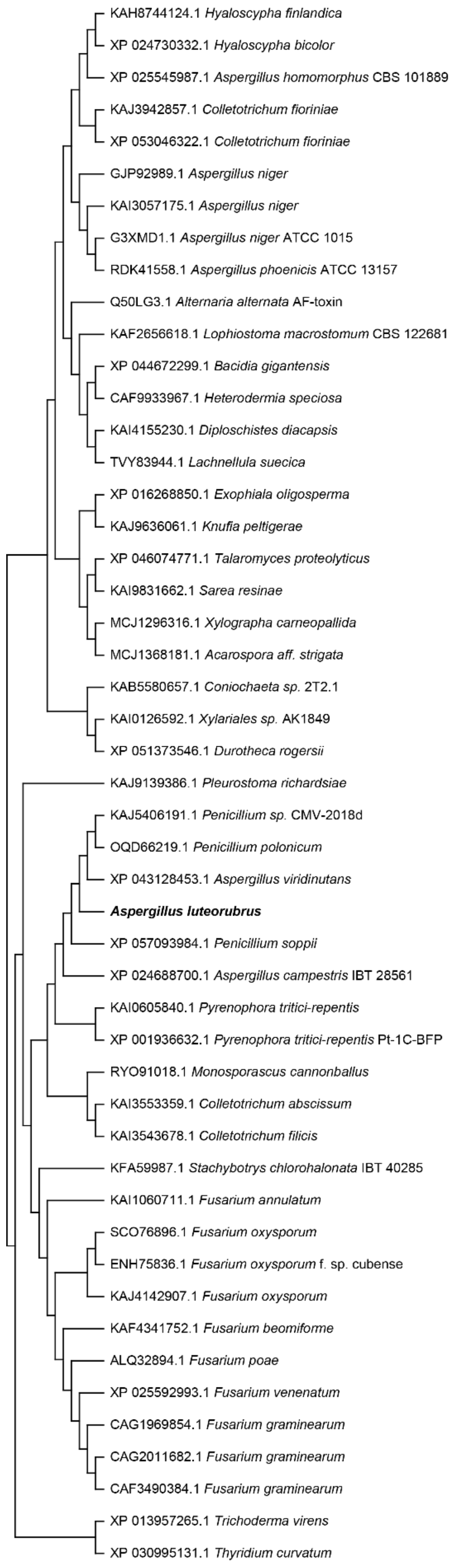

Figure S16. Phylogenetic tree for the MT domains extracted from various HR-PKSs of fungi. The tree was constructed using Neighbour Joining method and 1000 bootstrap in MEGA-11.

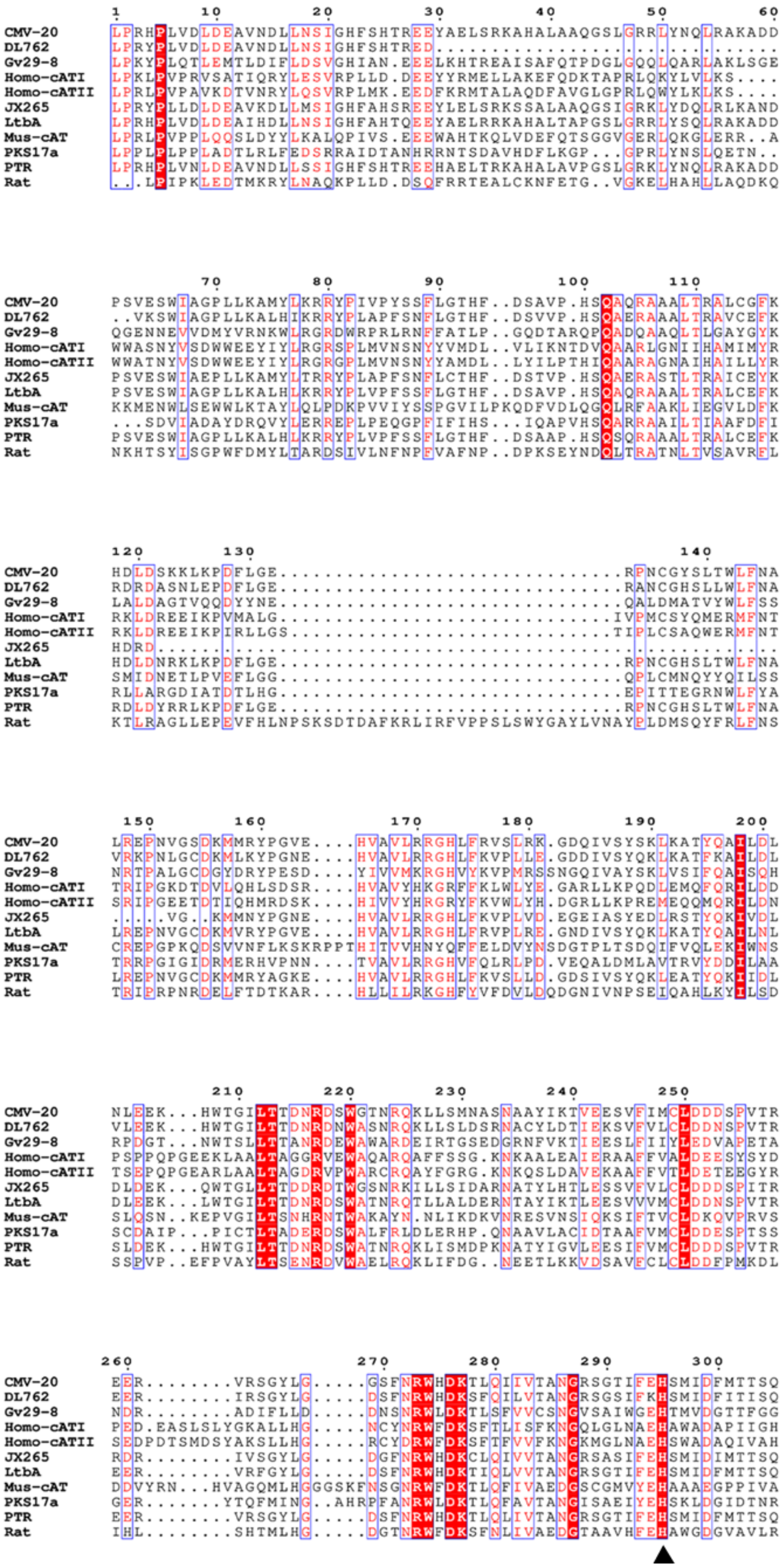

Figure continued on next page…

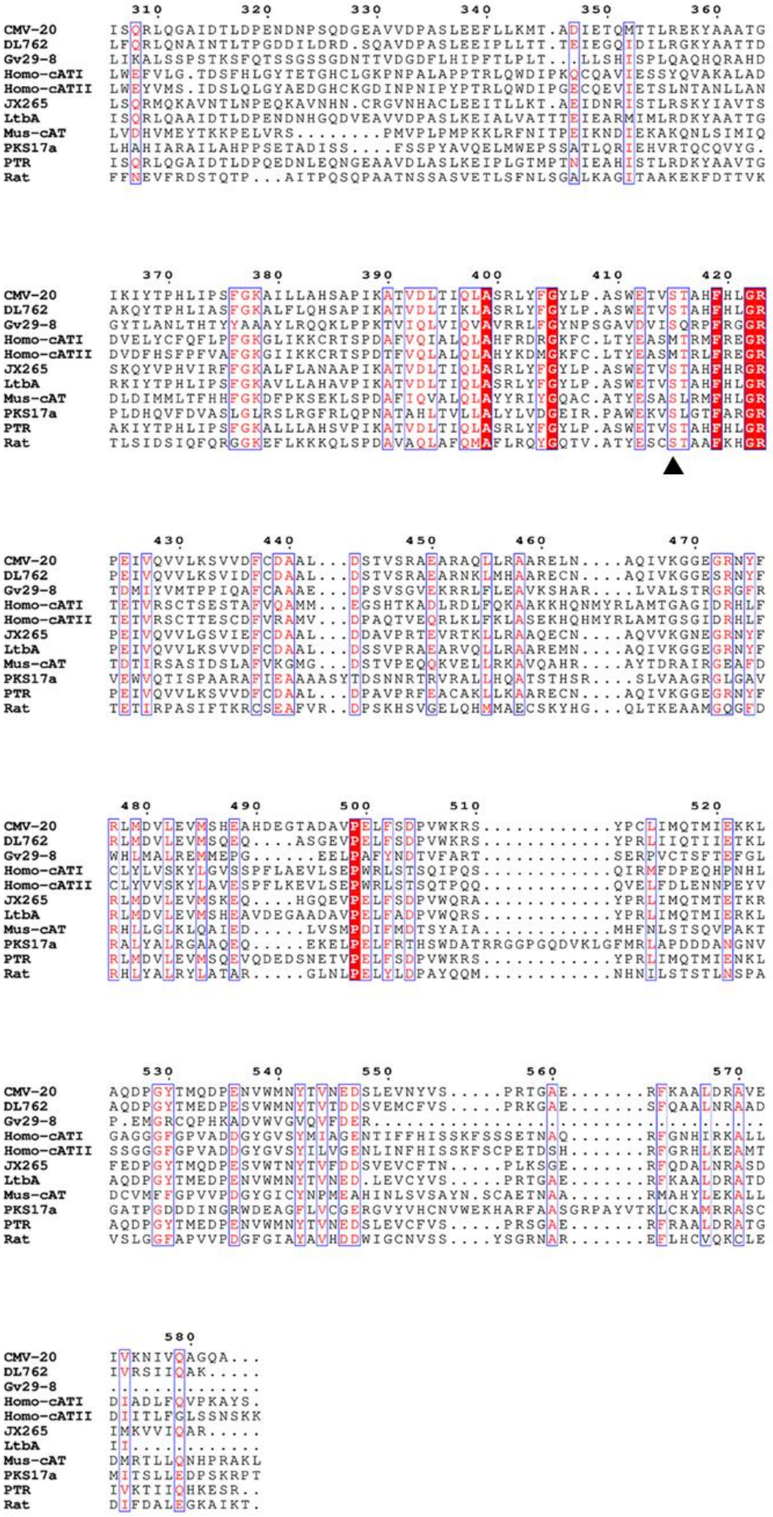

Figure S17. Amino acid sequence alignment of the LtbA cAT domain with other fungi. Abbreviations: CMV-20 is cAT domain from *Penicillium* sp. CMV-2018d (KAJ5406191.1), DL762 is cAT domain from *Monosporascus cannonballus* (RYO87181.1), Gv29-8 is the cAT domain from *Trichoderma virens* Gv29-8 (XP013957265.1), Homo-cATI is the cAT domain from *Homo sapiens* carnitine *O*-palmitoyltransferase (NP001138609), Homo-cATII is the cAT domain from human Liver-type carnitine *O*-palmitoyltransferase 1 (AAC41748.1), JX265 is the cAT domain from *Neoarthrinium*

moseri (KAI1853860.1), LtbA is the cAT domain from *Aspergillus luteorubrus*; Mus-cAT is the cAT domain from *Mus musculus* (CAA59971.1), PKS17a is the cAT domain from *Metarhizium anisopliae* KFG78606.1, PTR is cAT domain from *Pyrenophora tritici-repentis* strain M4 KAF7565337.1, Rat is the carnitine palmitoyltransferase 2 from *Rattus norvegicus* (EDL90417.1).The conserved catalytic residue His of the canonical carnitine *O*-acyltransferase is highlighted with black arrow. The serine proposed by Hang et al. to coordinate water molecule in the active site is also highlighted with black arrow.[12] The alignment was performed using ClustalW alignment tool, which is built-in MEGA-11 software,[15] and visualised by ESPript.[18]

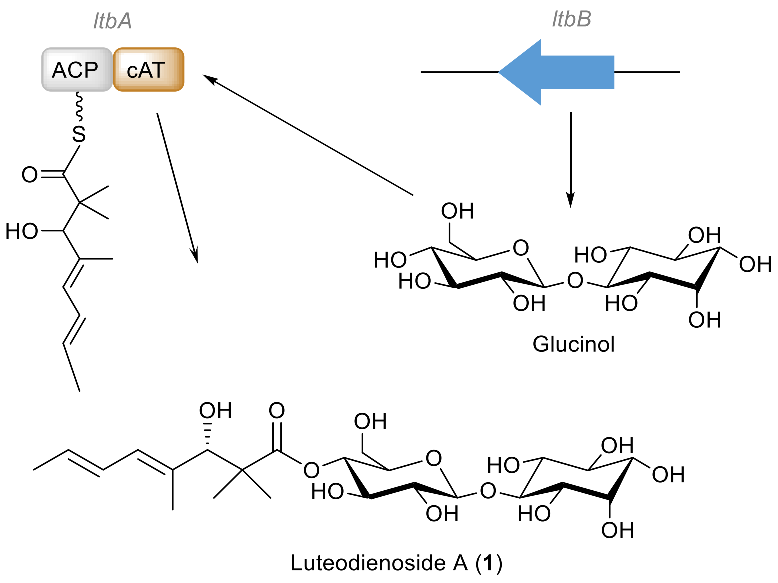

Figure S18. Proposed product offloading mechanism catalysed by the LtbA-cAT domain.

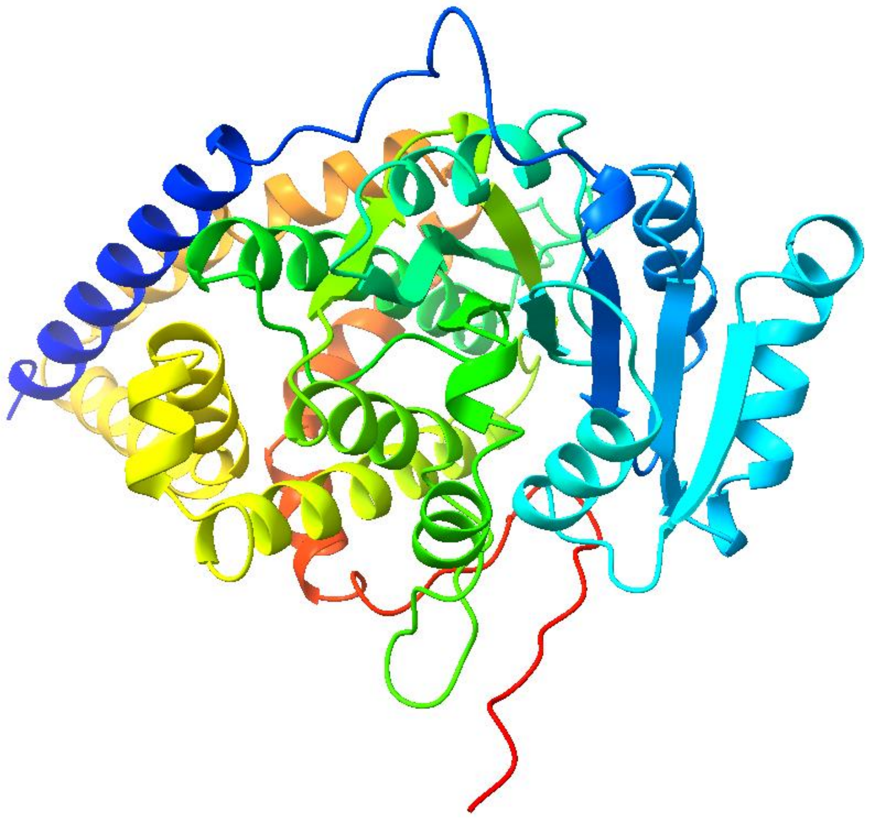

Figure S19. 3D structural modelling of LtbB using AlphaFold.[19]

Top model

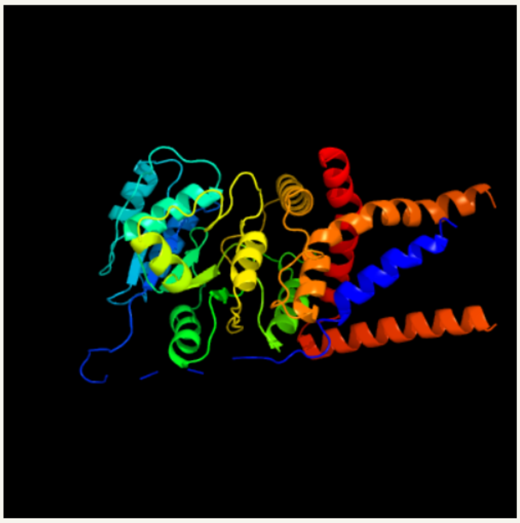

Image coloured by rainbow N → C terminus

Model dimensions (Å): **X**:51.519 **Y**:58.216 **Z**:79.390

Model (left) based on template c8dqkA

Top template information

PDB header: transferase
Chain: A: **PDB Molecule:** cellulose synthase-like cslf6;
PDBTitle: intermediate resolution structure of barley (1,3;1,4)-beta-glucan2 synthase cslf6.
PDB Entry: PDBe RCSB PDBj
-

Confidence and coverage

Confidence:	100.0%	Coverage:	93%

379 residues (93% of your sequence) have been modelled with 100.0% confidence by the single highest scoring template.

3D viewing

Interactive 3D view in JSmol

For other options to view your downloaded structure offline see the FAQ

#	Template	Alignment Coverage	3D Model	Confidence	% i.d.	Template Information
1	c8dqkA	Alignment	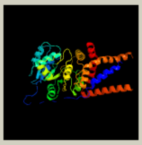	100.0	16	**PDB header:** transferase **Chain:** A: **PDB Molecule:** cellulose synthase-like cslf6; **PDBTitle:** intermediate resolution structure of barley (1,3;1,4)-beta-glucan2 synthase cslf6. **PDB Entry:** PDBe RCSB PDBj Run Investigator
2	c7xs7A	Alignment	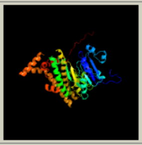	100.0	16	**PDB header:** membrane protein **Chain:** A: **PDB Molecule:** chitin synthase 1; **PDBTitle:** structure of a membrane-integrated glycosyltransferase **PDB Entry:** PDBe RCSB PDBj Run Investigator
3	c7stmA	Alignment	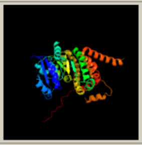	100.0	16	**PDB header:** transferase **Chain:** A: **PDB Molecule:** chitin synthase; **PDBTitle:** chitin synthase 2 from candida albicans bound to udp-glcnac **PDB Entry:** PDBe RCSB PDBj Run Investigator
4	c7stlB	Alignment	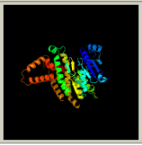	100.0	16	**PDB header:** membrane protein **Chain:** B: **PDB Molecule:** chitin synthase; **PDBTitle:** chitin synthase 2 from candida albicans at the apo state **PDB Entry:** PDBe RCSB PDBj Run Investigator

Transmembrane helices have been predicted in your sequence to adopt the topology shown below

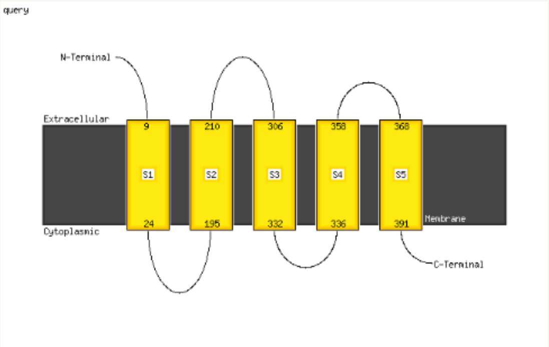

Figure S20. 3D structure of LtbB by Phyre2 modelling and the top 4 homologous template proteins. [20]

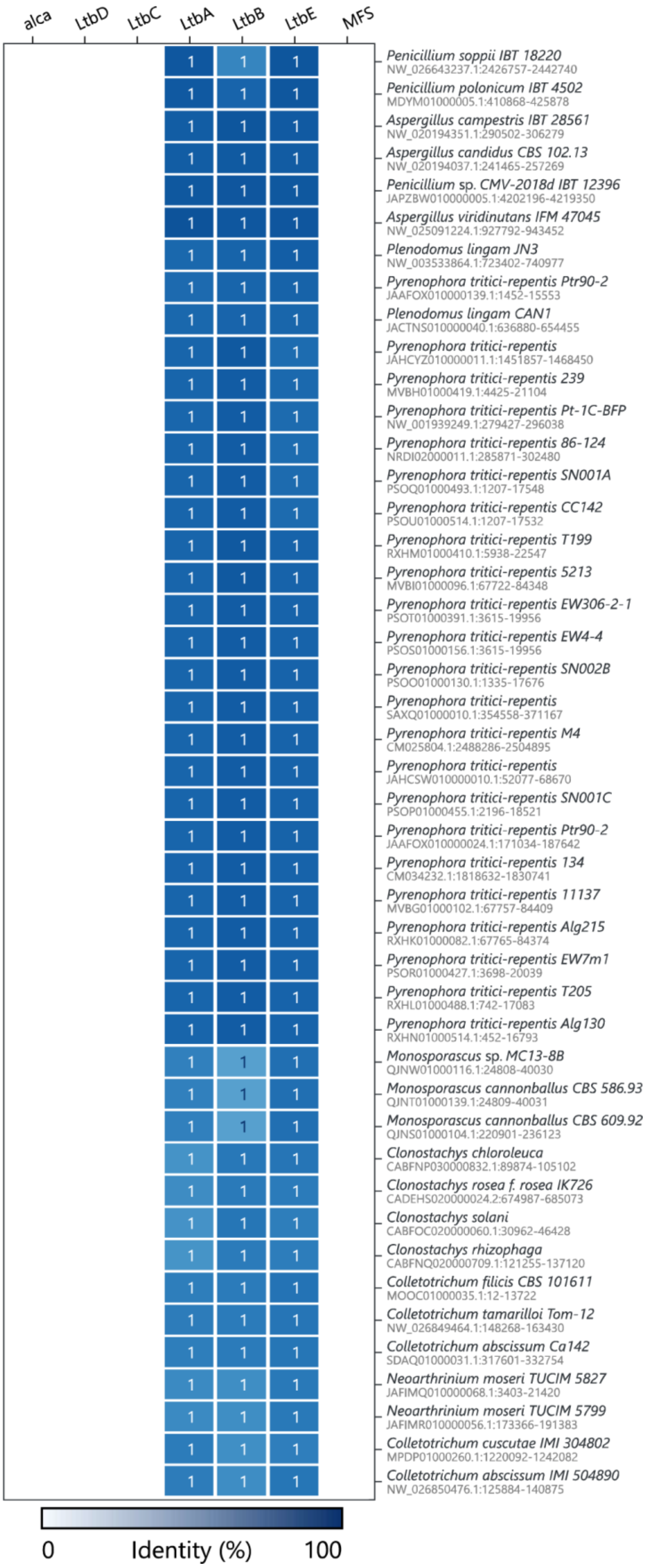

Figure S21. Homologous gene clusters identified in public genome databases via cblaster search showing the upstream and the downstream boundary of the *ltb* cluster homologous in other fungi.

```
Feature 1              #######                           ##             ###           #
query       44 ILDVGCGPGTITVG.[7].GRT     IGIDI.[ 24].IIFEEGNI.[10].FDIVFCA.[13].KALTEMR.[6].GI 149
2DPM_A      39 YFEPFVGGGALFFD.[3].KDA     VINDF.[107].LEIKVGDF.[ 9].GDFVYFD.[26].RLRDAFK.[6].AY 235 Streptococcus pneumoniae
1EJ0_A      26 VVDLGAAPGGWSQY.[8].GRI     IACDL.[  8].VDFLQGDF.[17].VQVVMSD.[21].LALEMCR.[6].GS 131 Escherichia coli
1P91_B      89 VLDIGCGEGYYTHA.[7].ITT     FGLDV.[ 14].VTFCVASS.[ 9].XDAIIRI.[ 3].CKAEELA.[6].GW 173 Escherichia coli
1SQG_A     250 ILDLCAAPGGKTTH.[7].AQV     VAVDI.[ 18].ATVKQGDG.[11].FDRILLD.[32].EILDAIW.[6].GT 369 Escherichia coli
2P7H_A      46 LLELGSFKGDFTSR.[4].FND.[1].TCVEA.[ 15].ITYIHSRF.[ 8].YDNIVLT.[10].ALLKRIN.[7].GR 136 Pectobacterium atrosept...
CAC11769    24 IVDLGCGPGFFTLP.[6].GKV     YAVDA.[ 17].IIPVKARA.[ 9].VDLVFIA.[10].AVLGEIY.[6].GY 117 Thermoplasma acidophilum
NP_249080   18 VLDLGCGDGELLAW.[6].VSG     YGLEI.[ 12].VNVIEQDL.[11].FDVVVMT.[10].KILEEML.[6].II 108 Pseudomonas aeruginosa ...
CAD21381    95 VLDIGTGTGTWAID.[7].AEI     IGTDL.[ 11].VRFFVEDS.[10].FDYIHTR.[11].QIIQRAF.[6].GW 185 Neurospora crassa

Feature 1
query      150 L 150
2DPM_A     236 V 236 Streptococcus pneumoniae
1EJ0_A     132 F 132 Escherichia coli
1P91_B     174 V 174 Escherichia coli
1SQG_A     370 L 370 Escherichia coli
2P7H_A     137 L 137 Pectobacterium atrosepticum
CAC11769   118 L 118 Thermoplasma acidophilum
NP_249080  109 T 109 Pseudomonas aeruginosa PAO1
CAD21381   186 L 186 Neurospora crassa
```

Figure S22. Conserved domain search for LtbC showing the catalytic residues of the enzyme.

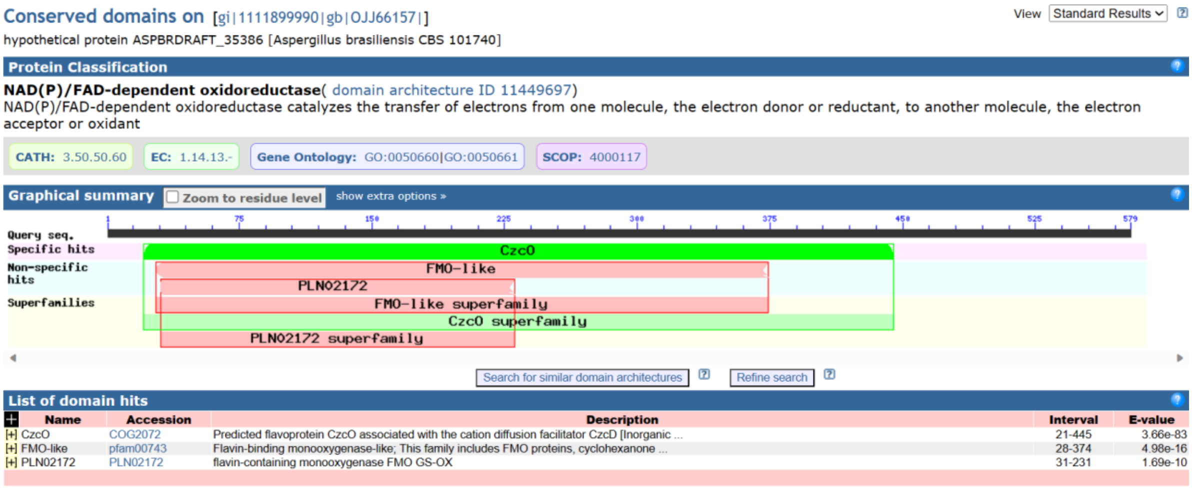

Figure S23. Conserved domain analysis of the LtbD homologue in *A. brasiliensis* CBS 101740 (OJJ66157.1) showing the predicted function of the enzyme to be an NAD(P)/FAD-dependent oxidoreductase.

```
Consensus                            MTISHKNDTSNGADQPKCRESPIHANRKMRVIVIGAGASGIYMAYKLKYSFTDVVLDIYE   60
Aspergillus brasiliensis (OJJ66157.1) MTISHKNDTSNGADQPKCRESPIHANRKMRVIVIGAGASGIYMAYKLKYSFTDVVLDIYE   60
ltbD                                 ------------------------------------------------------------

Consensus                            KNSDIGGTWFENRYPGCACDVPAHNYTYSFEPKTDWSANYASSREIFTYFNNFLDKYDLR  120
Aspergillus brasiliensis (OJJ66157.1) KNSDIGGTWFENRYPGCACDVPAHNYTYSFEPKTDWSANYASSREIFTYFNNFLDKYDLR  120
ltbD                                 ------------------------------------------------------------

Consensus                            GYISLRHEVIGAHWEEDPGEWVVQVRKQNRSIFEQRCDFVINAAGILNAWRWPPIPGLQS  180
Aspergillus brasiliensis (OJJ66157.1) GYISLRHEVIGAHWEEDPGEWVVQVRKQNRSIFEQRCDFVINAAGILNAWRWPPIPGLQS  180
ltbD                                 ------------------------------------------------------------

Consensus                            FKGTLLHSAAWDESIDLIGKRVGLIGNGSSGIQILPQVQKVAKHVTTFIRAPTWVSPTLG  240
Aspergillus brasiliensis (OJJ66157.1) FKGTLLHSAAWDESIDLIGKRVGLIGNGSSGIQILPQVQKVAKHVTTFIRAPTWVSPTLG  240
ltbD                                 ------------------------------------------------------------

Consensus                            MEPREYSEEEKKTFKEQPGVLLEMRKATERAMGAGFPLMLQGSETQIQTAAYMREQMIKK  300
Aspergillus brasiliensis (OJJ66157.1) MEPREYSEEEKKTFKEQPGVLLEMRKATERAMGAGFPLMLQGSETQIQTAAYMREQMIKK  300
ltbD                                 ------------------------------------------------------------

Consensus                            INNDELASKLIPDFALGCRRLTPGVNYLESLTLPNVTPIYANITKVTPTSCVTDNGVETD  360
Aspergillus brasiliensis (OJJ66157.1) INNDELASKLIPDFALGCRRLTPGVNYLESLTLPNVTPIYANITKVTPTSCVTDNGVETD  360
ltbD                                 ------------------------------------------------------------

Consensus                            LDVLICATGFDTTFRPRFPVIGRDGRNLQTEWKDEPRSYLGLAASGFPNYFMFLGPNXPI  420
Aspergillus brasiliensis (OJJ66157.1) LDVLICATGFDTTFRPRFPVIGRDGRNLQTEWKDEPRSYLGLAASGFPNYFMFLGPNCPI  420
ltbD                                 ---------------------------------------------------MFLGPNGPI    9

Consensus                            GNGPIIFSIXLQGSYFAZFLNRWQXEDIXAFDXKXDAVBDFMEQKDXFMZXTVWNTXCQS  480
Aspergillus brasiliensis (OJJ66157.1) GNGPIIFSIELQGSYFAEFLNRWQKEDIKAFDAKIDAVDDFMEQKDRFMQKTVWNTNCQS  480
ltbD                                 GNGPIIFSIKLQGSYFAQFLNRWQNEDIQAFDPKVDAVNDFMEQKDLFMETTVWNTHCQS   69

Consensus                            WYKXXQTGKITALWPGSTLHYMETLAKPRYDDFXXTYASKNRFAYLGNGFSQXEXNPXAD  540
Aspergillus brasiliensis (OJJ66157.1) WYKNPQTGKITALWPGSTLHYMETLAKPRYDDFHVTYASKNRFAYLGNGFSQHEMNPKAD  540
ltbD                                 WYKSRQTGKITALWPGSTLHYMETLAKPRYDDFDITYASKNRFAYLGNGFSQDELNPQAD  129

Consensus                            JXYYIRXXDDGXSVFGNLFSTYNAKDIGDKMTAXADRXI                      579
Aspergillus brasiliensis (OJJ66157.1) LAYYIREQDDGSSVFGNLFSTYNAKDIGDKMTAVADRGI                      579
ltbD                                 ITYYIRDKDDGLSVFGNLFSTYNAKDIGDKMTALADRAI                      168
```

Figure S24. Pairwise protein sequence alignment of LtbD and the homologue in *A. brasiliensis* CBS 101740 (OJJ66157.1).

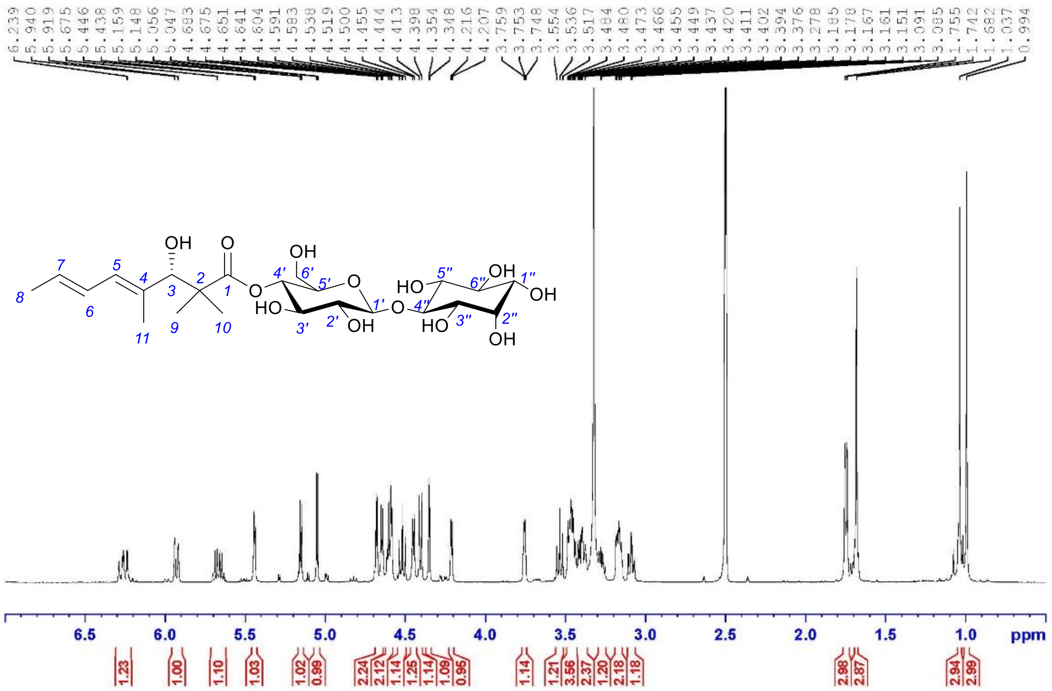

Figure S25. ^{1}H NMR (600 MHz, DMSO-d_6) spectrum of luteodienoside A (**1**)

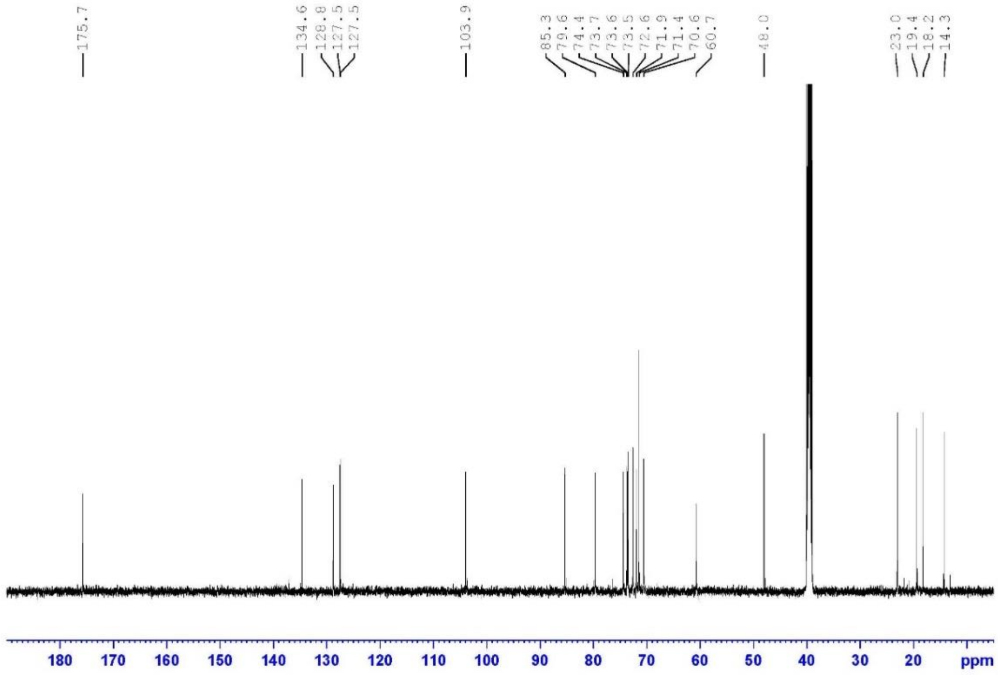

Figure S26. ^{13}C NMR (150 MHz**,** DMSO-d_6) spectrum of luteodienoside A (**1**)

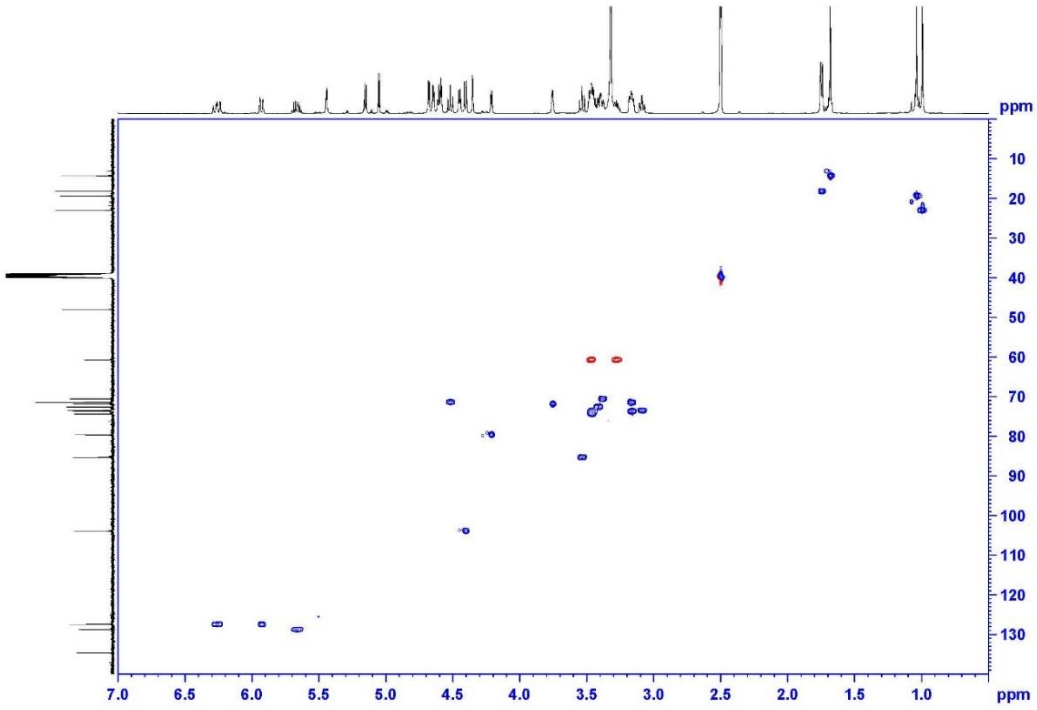

Figure S27. HSQC NMR (600 MHz, DMSO-d_6) spectrum of luteodienoside A (**1**)

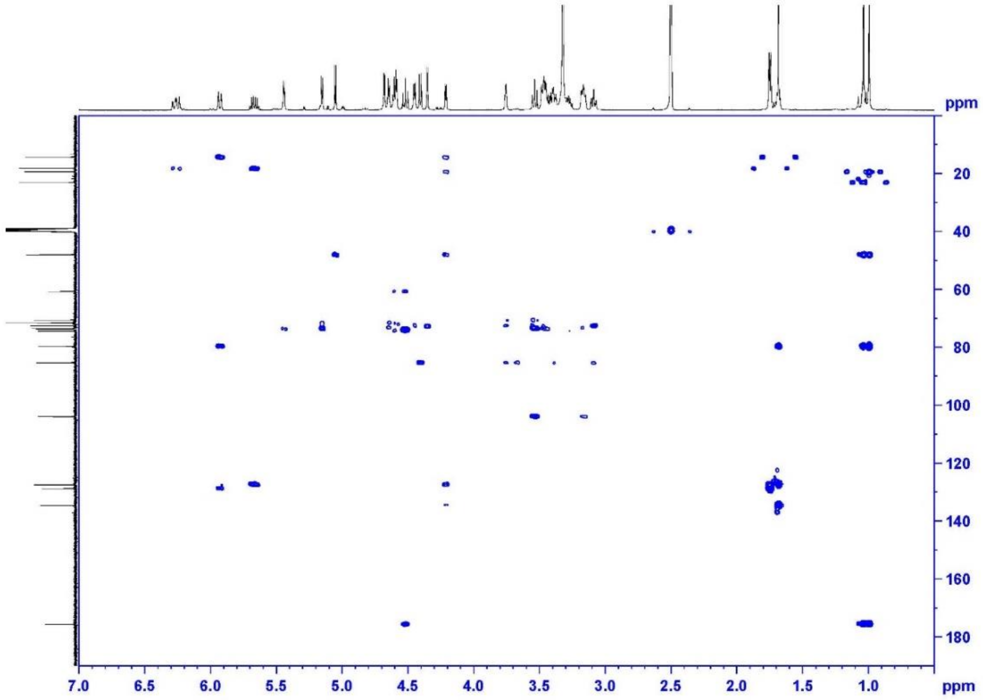

Figure S28. HMBC NMR (600 MHz, DMSO-d_6) spectrum of luteodienoside A (**1**)

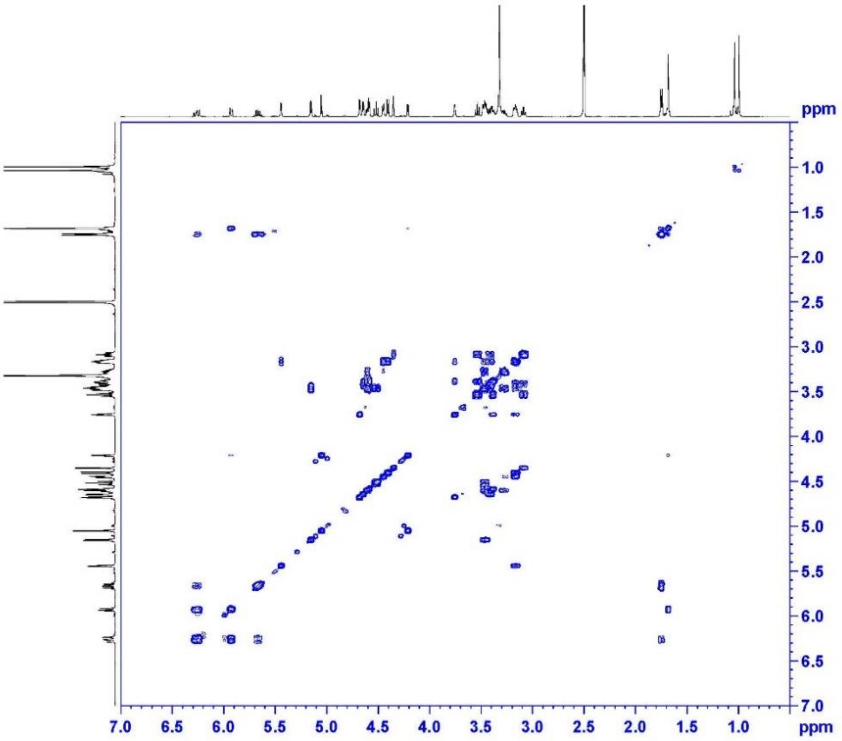

Figure S29. ^{1}H-^{1}H COSY NMR (600 MHz, DMSO-d_6) spectrum of luteodienoside A (**1**)

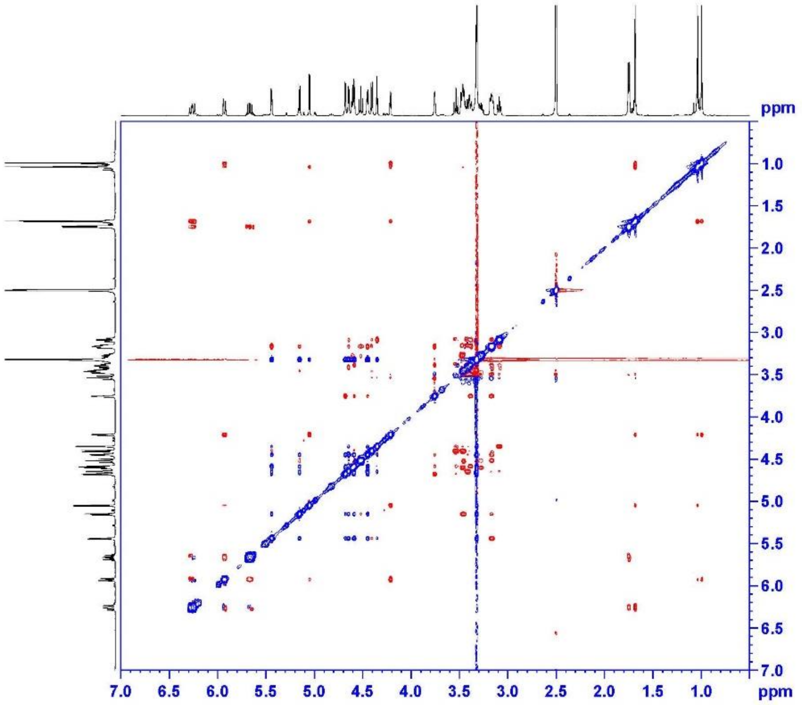

Figure S30. ^{1}H-^{1}H ROESY NMR (600 MHz, DMSO-d_6) spectrum of luteodienoside A (**1**)

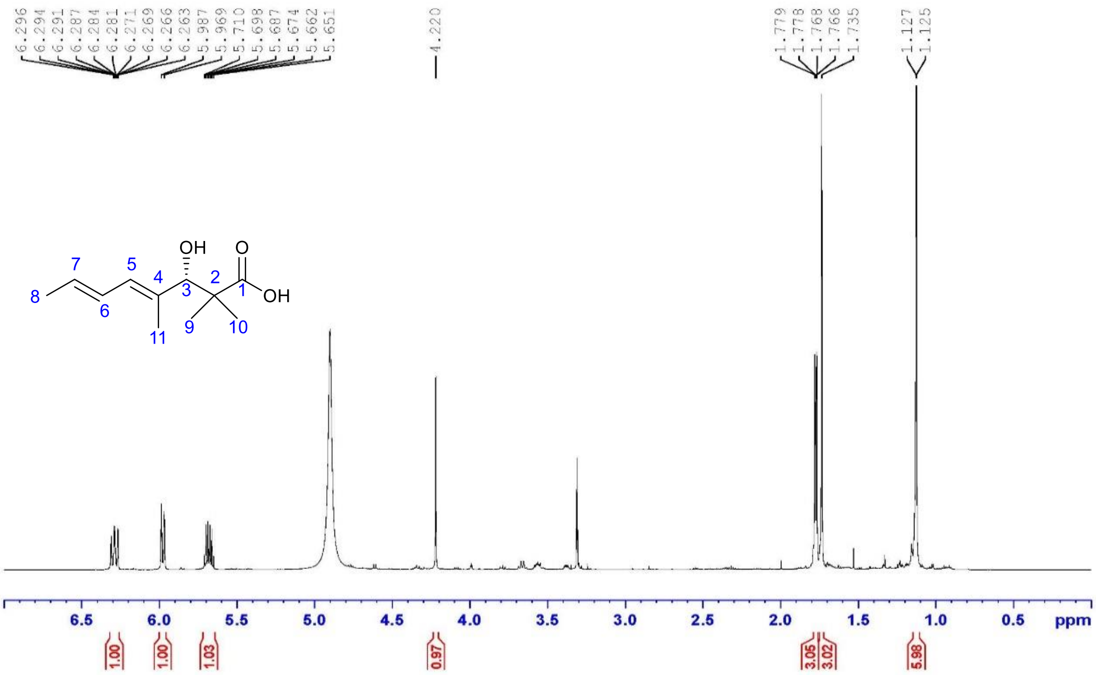

Figure S31. ^{1}H NMR (600 MHz, CD_3OD) spectrum of luteodiene A (**2**)

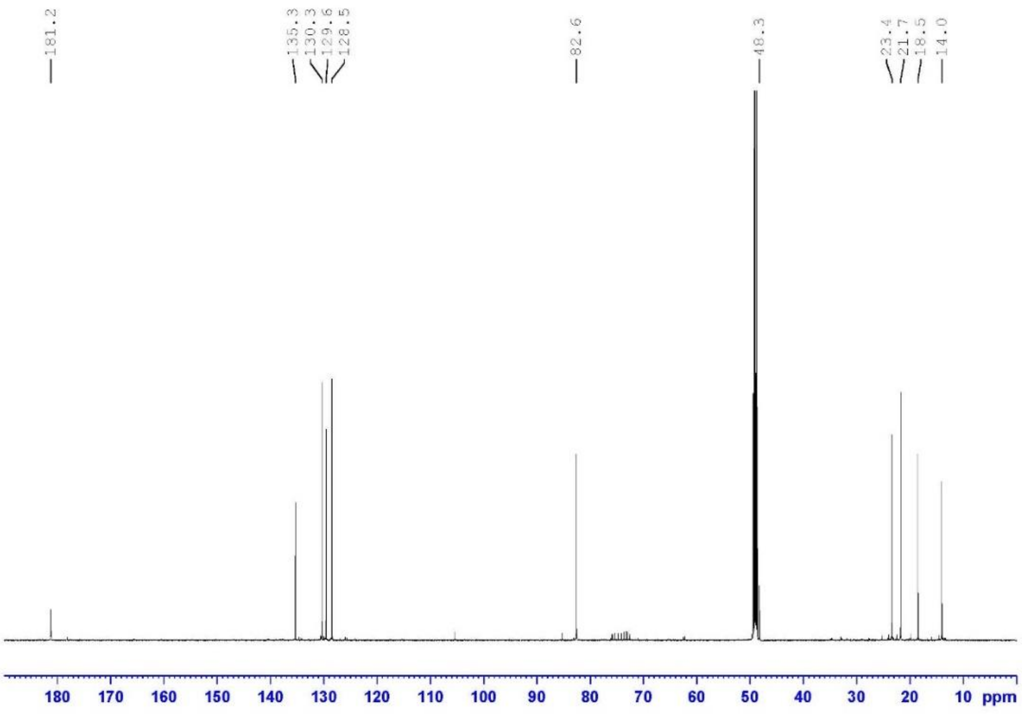

Figure S32. ^{13}C NMR (150 MHz, CD_3OD) spectrum of luteodiene A (**2**)

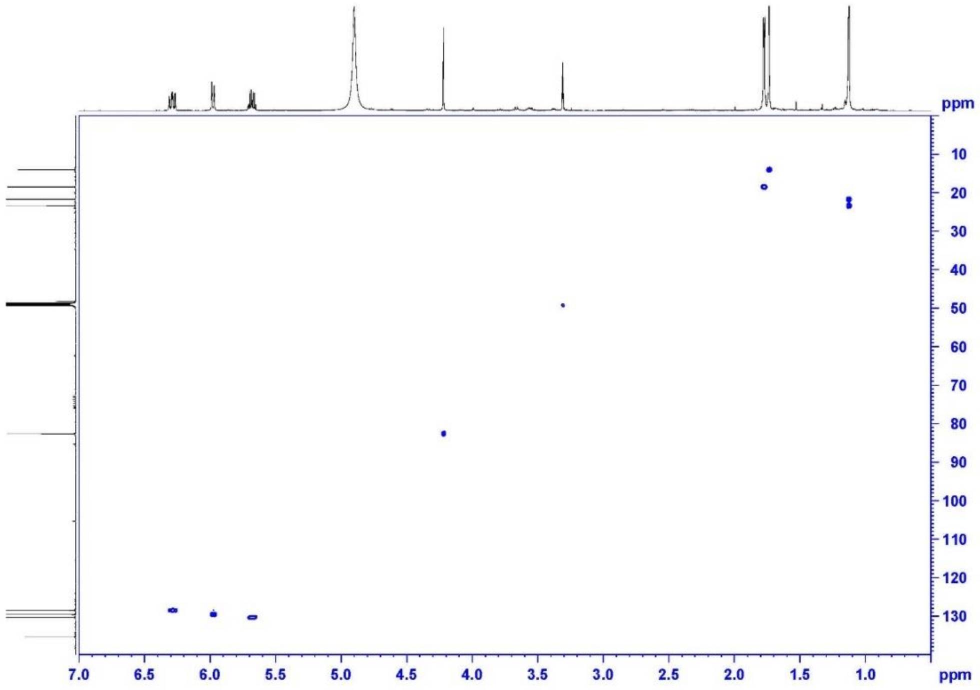

Figure S33. HSQC NMR (600 MHz, CD_3OD) spectrum of luteodiene A (**2**)

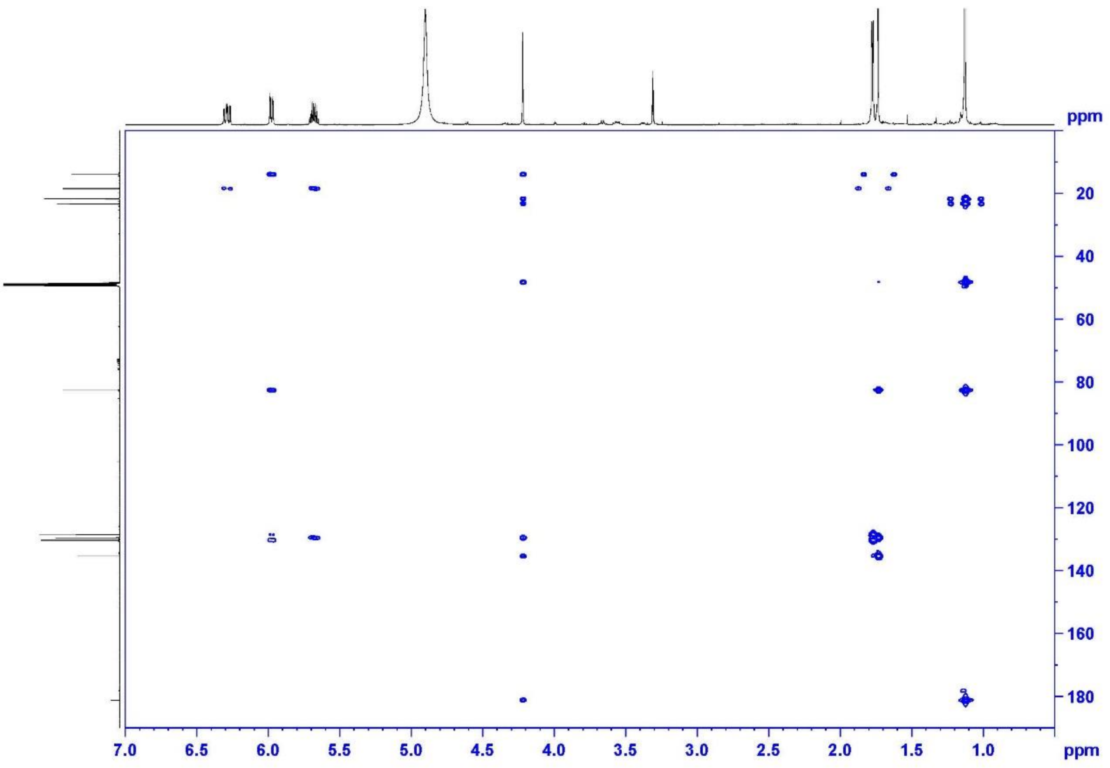

Figure S34. HMBC NMR (600 MHz, CD_3OD) spectrum of luteodiene A (**2**)

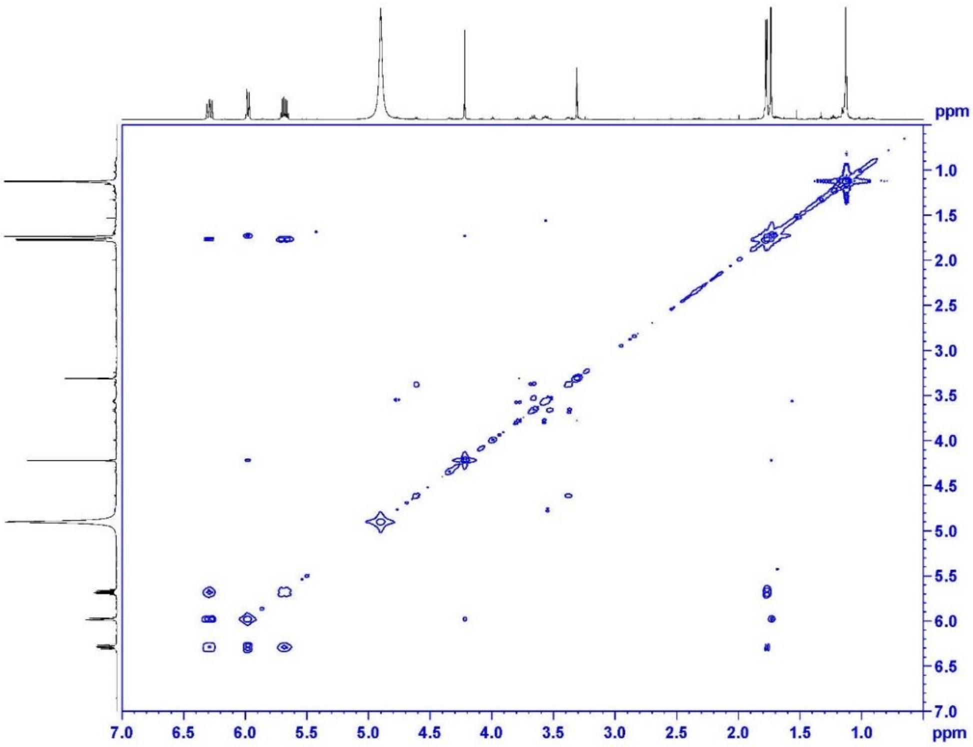

Figure S35. ^1H-^1H COSY NMR (600 MHz, CD_3OD) spectrum of luteodiene A (**2**)

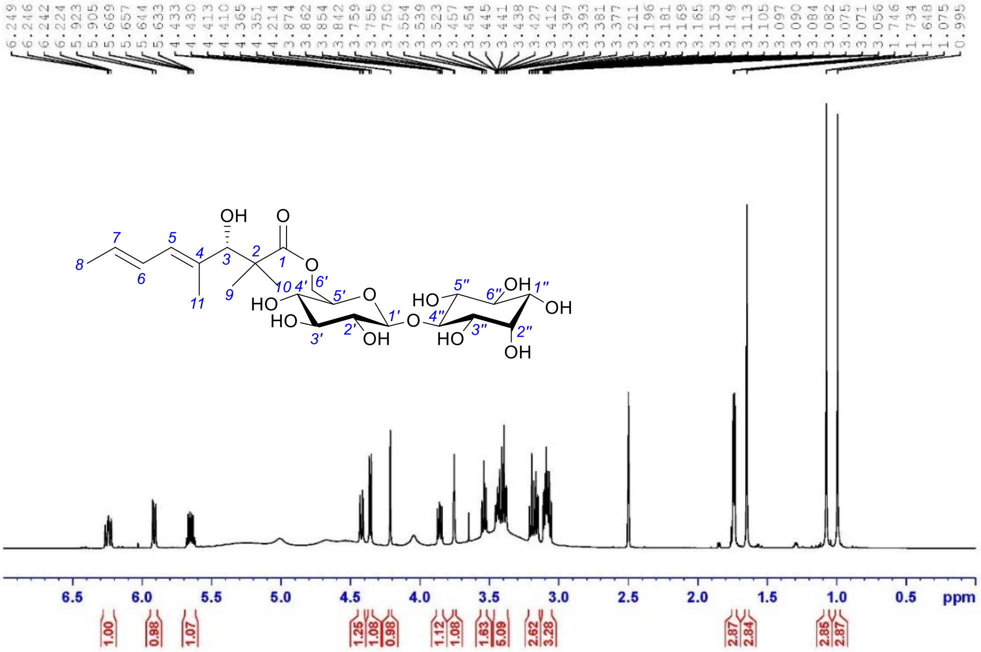

Figure S36. ^1H NMR (600 MHz, DMSO-d_6) spectrum of luteodienoside B (**3**)

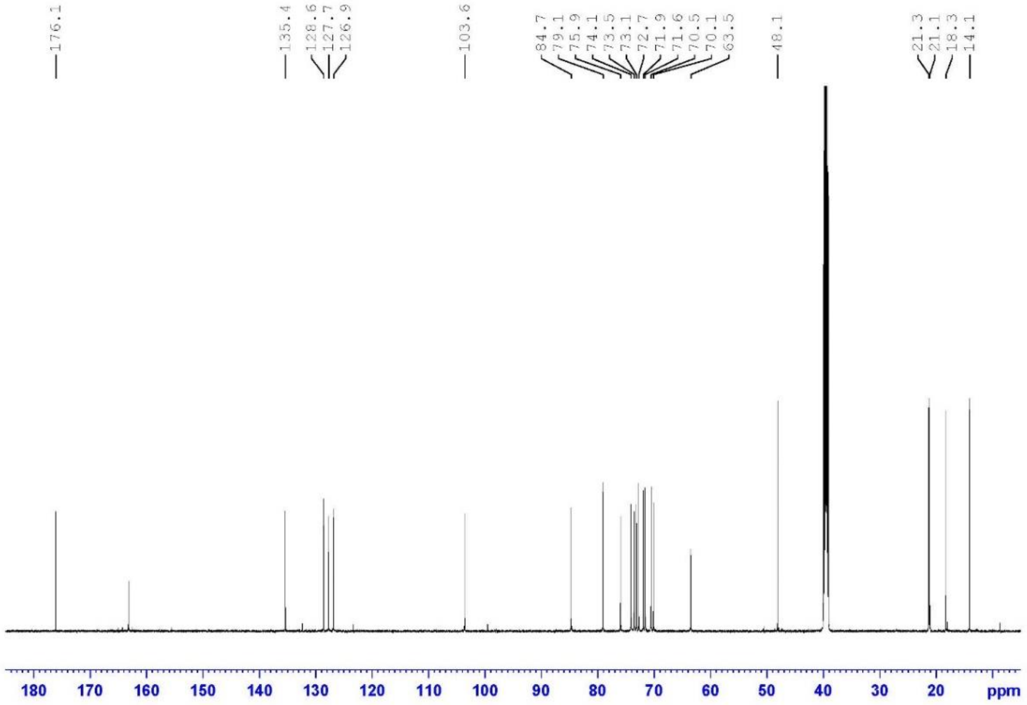

Figure S37. ^{13}C NMR (150 MHz, DMSO-d_6) spectrum of luteodienoside B (**3**)

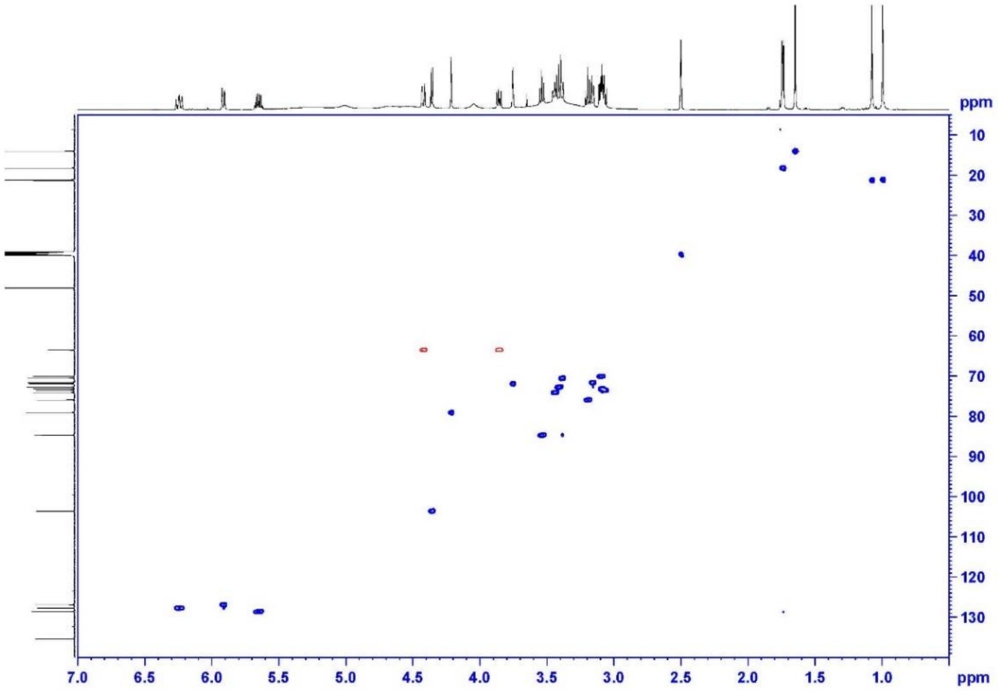

Figure S38. HSQC NMR (600 MHz, DMSO-d_6) spectrum of luteodienoside B (**3**)

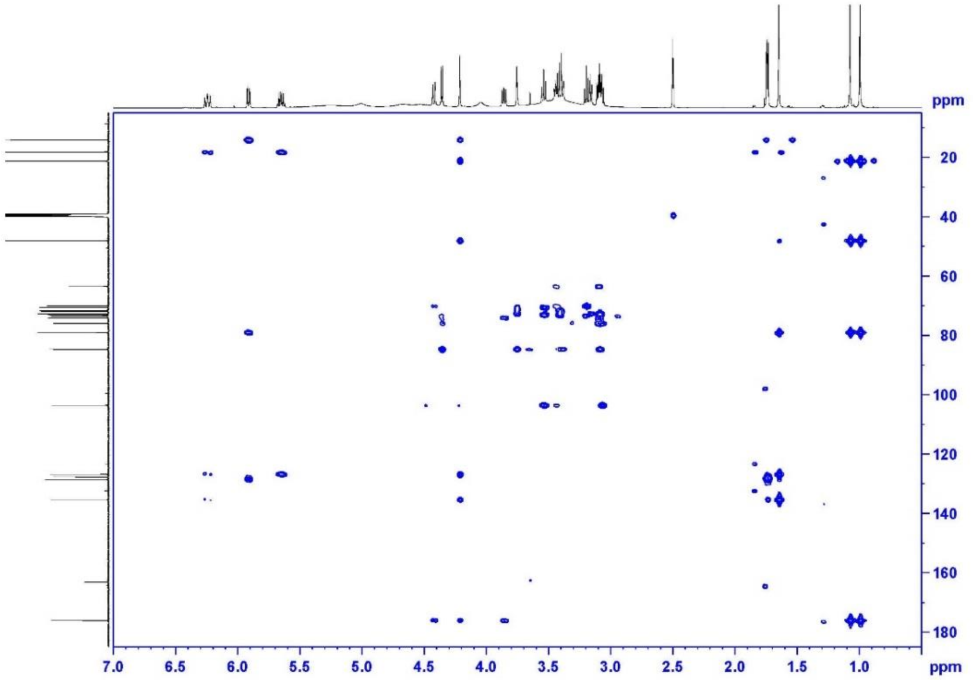

Figure S39. HMBC NMR (600 MHz, DMSO-d_6) spectrum of luteodienoside B (**3**)

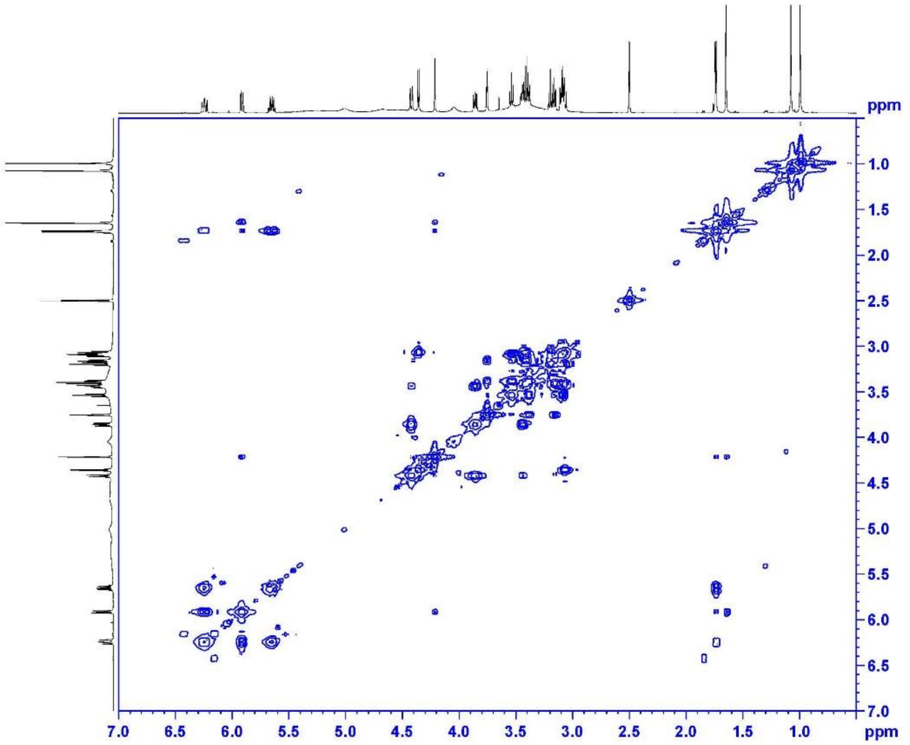

Figure S40. ^{1}H-^{1}H COSY NMR (600 MHz, DMSO-d_6) spectrum of luteodienoside B (**3**)

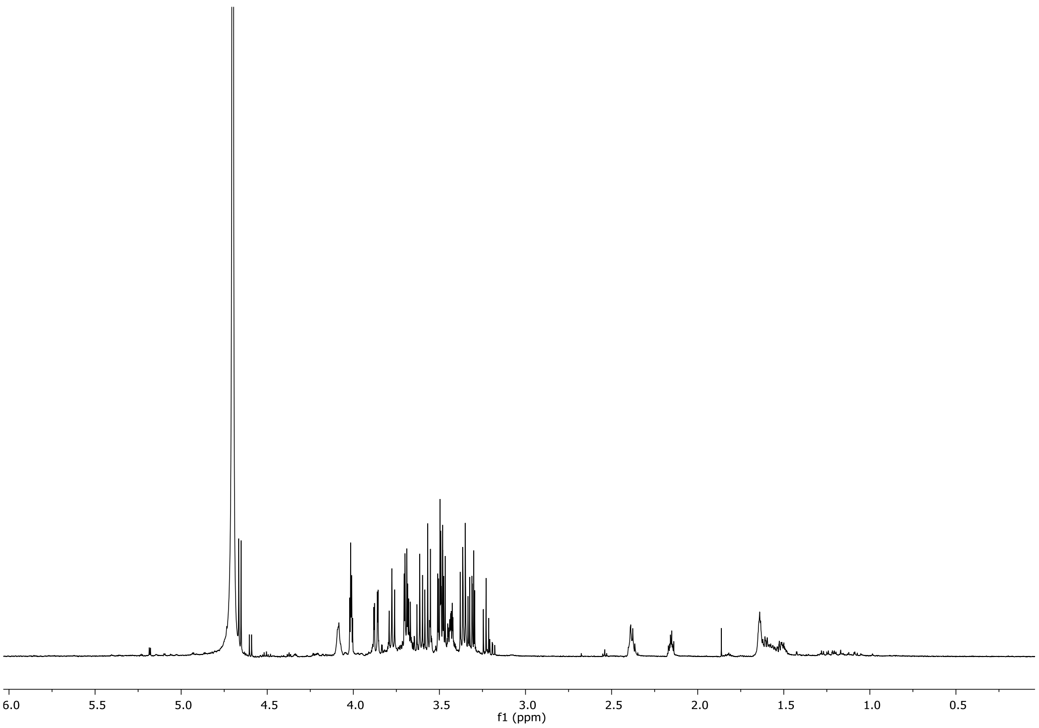

Figure S41. ^{1}H NMR spectrum of glucinol in D_2O.

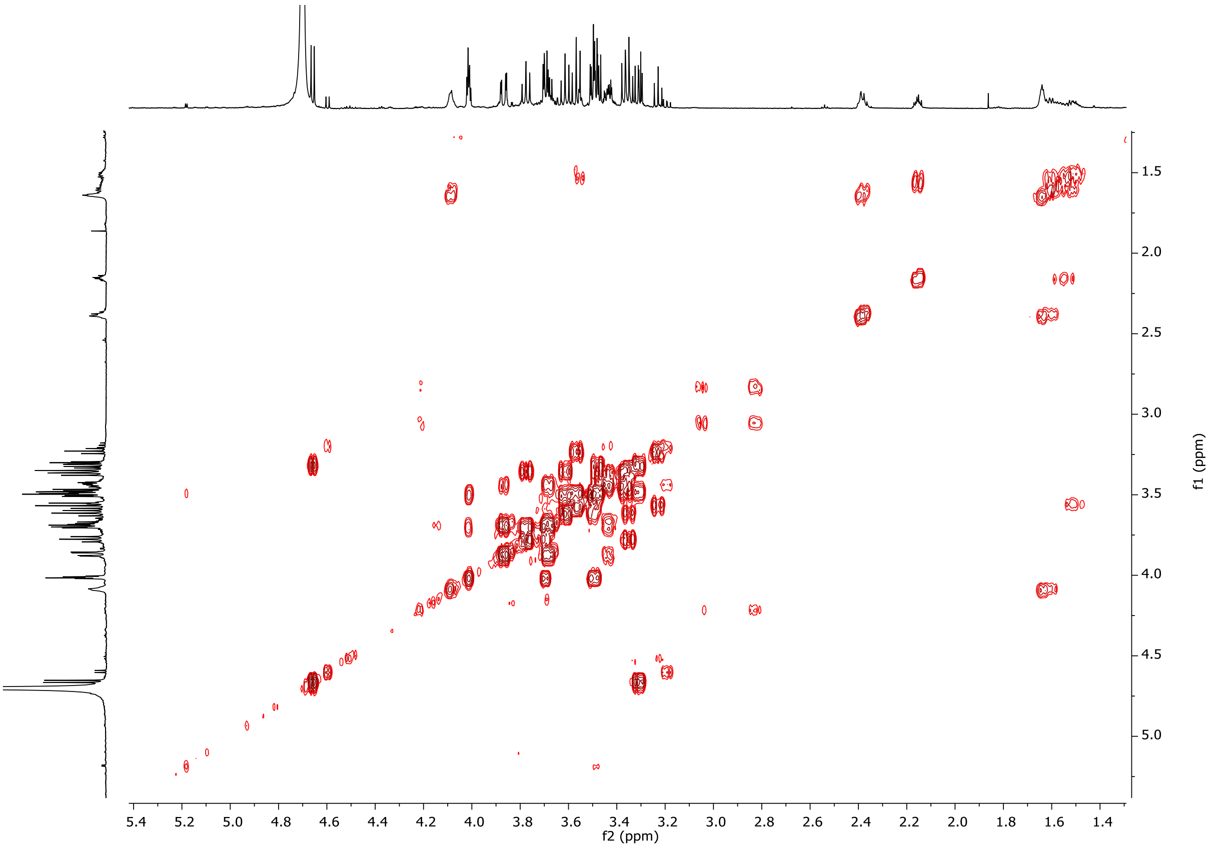

Figure S42. ^{1}H-^{1}H COSY NMR spectrum of glucinol in D_2O.

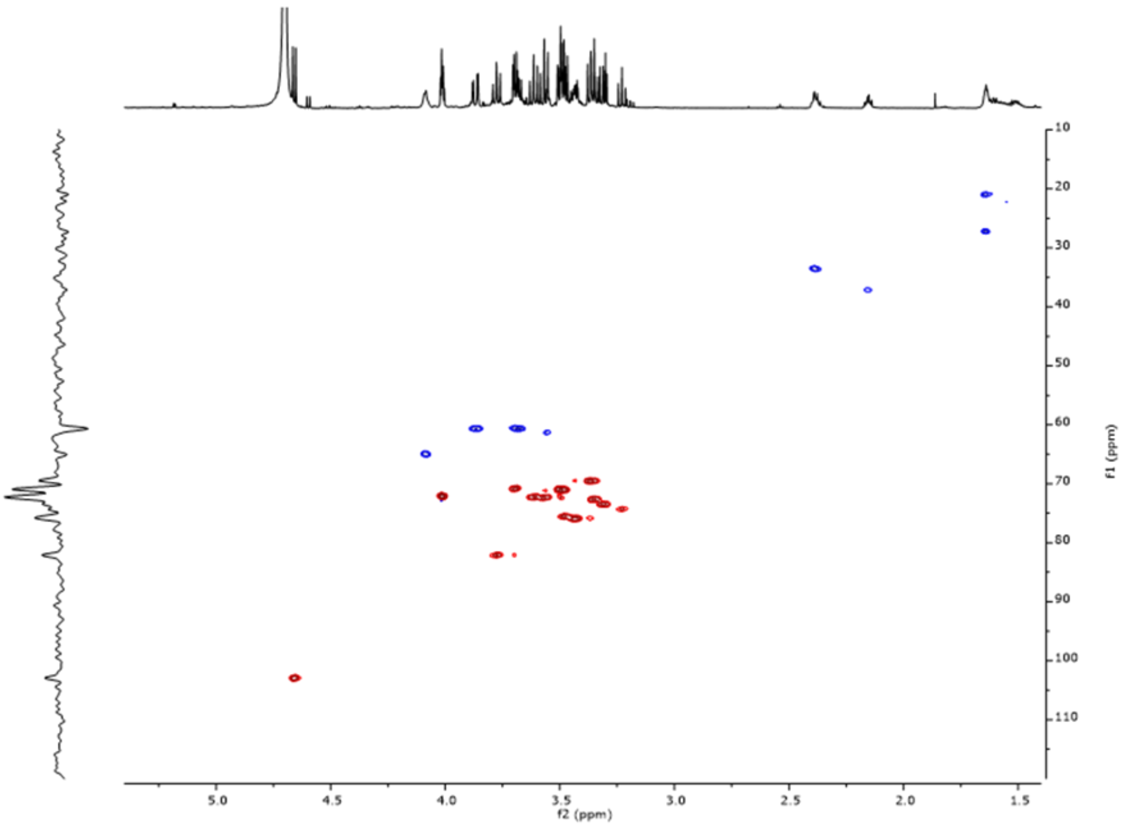

Figure S43. HSQC NMR spectrum of glucinol in D_2O.

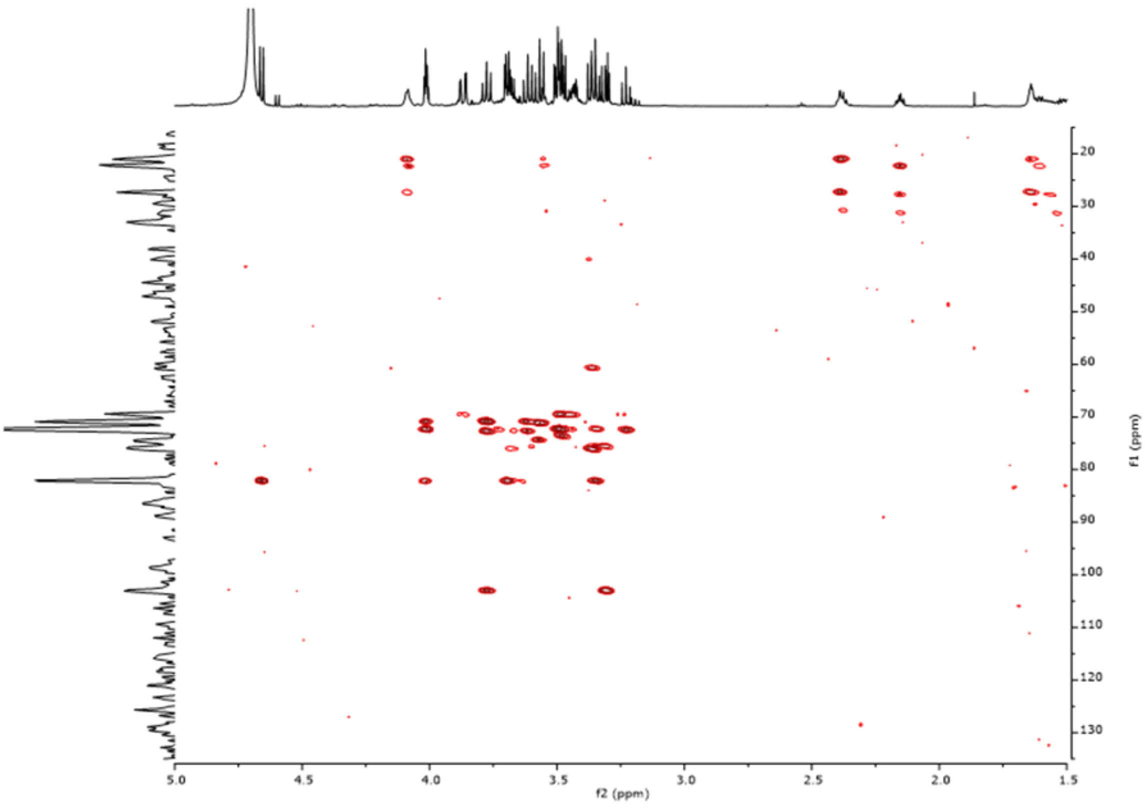

Figure S44. HMBC NMR spectrum of glucinol in D_2O.

References

1 P. W. Crous, M. J. Wingfield, Y.-H. Chooi, C. L. M. Gilchrist, E. Lacey, J. I. Pitt, F. Roets, W. J. Swart, J. F. Cano-Lira and N. Valenzuela-Lopez, *Persoonia*: *Molecular Phylogeny and Evolution of Fungi*, 2020, 44, 301.

2 Y. Chiang, M. Ahuja, C. E. Oakley, R. Entwistle, A. Asokan, C. Zutz, C. C. C. Wang and B. R. Oakley, *Angew. Chem. Int. Ed.*, 2016, **55**, 1662–1665.

3 S. M. Ma, J. W. Li, J. W. Choi, H. Zhou, K. K. Lee, V. A. Moorthie, X. Xie, J. T. Kealey, N. A. Da Silva and J. C. Vederas and Y. Tang, *Science*, 2009, 326, 589–592.

4 C. J. B. Harvey, M. Tang, U. Schlecht, J. Horecka, C. R. Fischer, H. C. Lin, J. Li, B. Naughton, J. Cherry, M. Miranda, Y. F. Li, A. M. Chu, J. R. Hennessy, G. A. Vandova, D. Inglis, R. S. Aiyar, L. M. Steinmetz, R. W. Davis, M. H. Medema, E. Sattely, C. Khosla, R. P. St Onge, Y. Tang and M. E. Hillenmeyer, *Sci. Adv.*, 2018, 4, e5459

5 I. Roux and Y.-H. Chooi, in *Engineering Natural Product Biosynthesis*, Springer, 2022, vol. 2489, pp. 75–92.

6 F. Y. Lim, J. F. Sanchez, C. C. C. Wang and N. P. Keller, in *Methods enzymol.*, Elsevier, 2012, vol. 517, pp. 303–324.

7 O. V. Dolomanov, L. J. Bourhis, R. J. Gildea, J. A. K. Howard and H. Puschmann, *J. Appl. Crystallogr.*, 2009, **42**, 339–341.

8 L. J. Bourhis, O. V. Dolomanov, R. J. Gildea, J. A. K. Howard and H. Puschmann, *Acta Crystallogr., Sect. A: Found. Adv.*, 2015, **71**, 59–75.

9 G. M. Sheldrick, *Acta Crystallogr., Sect. C: Struct. Chem.*, 2015, **71**, 3–8.

10 C. L. M. Gilchrist, T. J. Booth, B. van Wersch, L. van Grieken, M. H. Medema and Y.-H. Chooi, *Bioinf. Adv.*, 2021, 1, vbab016.

11 C. L. M. Gilchrist and Y.-H. Chooi, *Bioinformatics*, 2021, **37**, 2473–2475.

12 L. Hang, M.-C. Tang, C. J. B. Harvey, C. G. Page, J. Li, Y. -S. Hung, N. Liu, M. E. Hillenmeyer and Y. Tang, *Angew. Chem., Int. Ed.*, 2017, **56**, 9556–9560.

13 S. F. Altschul, W. Gish, W. Miller, E. W. Myers and D. J. Lipman, *J. Mol. Biol.*, 1990, 215, 403–410.

14 J. Wang, F. Chitsaz, M. K. Derbyshire, N. R. Gonzales, M. Gwadz, S. Lu, G. H. Marchler, J. S. Song, N. Thanki and R. A. Yamashita, *Nucleic Acids Res.*, 2023, **51**, D384–D388.

15 S. Kumar, G. Stecher, M. Li, C. Knyaz and K. Tamura, *Mol. Biol. Evol.*, 2018, 35, 1547– 1549.

16 H. Li, S. Shu, J. A. Kalaitzis, Z. Shang, D. Vuong, A. Crombie, E. Lacey, A. M. Piggott and Y.-H. Chooi, *Org. Lett.*, 2021, 23, 8789–8793.

17 H. Li, C. L. M. Gilchrist, C.-S. Phan, H. J. Lacey, D. Vuong, S. A. Moggach, E. Lacey, A. M. Piggott and Y.-H. Chooi, *J. Am. Chem. Soc.*, 2020, **142**, 7145–7152.

18 X. Robert and P. Gouet, *Nucleic Acids Res.*, 2014, **42**, W320-W324.

19 J. Jumper, R. Evans, A. Pritzel, T. Green, M. Figurnov, O. Ronneberger, K. Tunyasuvunakool, R. Bates, A. Žídek and A. Potapenko, *Nature*, 2021, **596**, 583–589.

20 L. A. Kelley, S. Mezulis, C. M. Yates, M. N. Wass and M. J. E. Sternberg, *Nat. Protoc.*, 2015, **10**, 845–858
